# Selective Thermal Deprotection of *N*-Boc Protected Amines in Continuous Flow

**DOI:** 10.1021/acs.oprd.3c00498

**Published:** 2024-04-25

**Authors:** Michelle-Rose Ryan, Denis Lynch, Stuart G. Collins, Anita R. Maguire

**Affiliations:** † School of Chemistry, Analytical and Biological Chemistry Research Facility, University College Cork, Cork T12 YN60, Ireland; ‡ School of Chemistry, Analytical and Biological Chemistry Research Facility, SSPC, The SFI Research Centre for Pharmaceuticals, University College Cork, Cork T12 YN60, Ireland; § School of Chemistry and School of Pharmacy, Analytical and Biological Chemistry Research Facility, SSPC, The SFI Research Centre for Pharmaceuticals, University College Cork, Cork T12 YN60, Ireland

**Keywords:** continuous flow deprotection, selective deprotection, amine, Boc deprotection, thermal deprotection

## Abstract

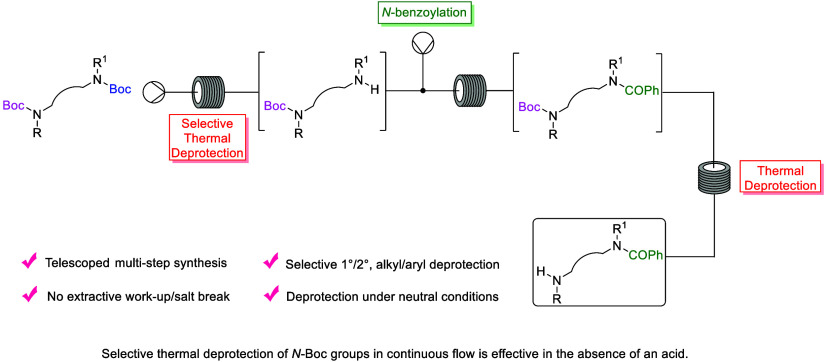

Thermal *N*-Boc deprotection of a range of amines is readily effected in continuous flow, in the absence of an acid catalyst. While the optimum results were obtained in methanol or trifluoroethanol, deprotection can be effected in a range of solvents of different polarities. Sequential selective deprotection of *N*-Boc groups has been demonstrated through temperature control, as exemplified by effective removal of an aryl *N*-Boc group in the presence of an alkyl *N*-Boc group. As a proof of principle, a telescoped sequence involving selective deprotection of an aryl *N*-Boc group from **9h** followed by benzoylation and deprotection of the remaining alkyl *N*-Boc group to form amide **13** proved successful.

## Introduction

The synthesis of complex organic compounds is often impeded by the presence of multiple reactive functionalities on a molecule that can undergo unwanted side reactions. In such instances, the employment of a selective protection/deprotection methodology is therefore critical in achieving chemoselectivity.^1−4^ Amines are inherently reactive species that may require temporary protection in multistep synthesis.^5^ Among the vast number of protecting groups available, *tert*-butyloxycarbonyl (Boc) protection has remained a consistently favored approach as the stable *N*-Boc product is resistant to nucleophilic attack, with Boc anhydride being the reagent frequently used for attachment of the Boc group.^5−8^ The subsequent removal of the *N*-Boc group is typically carried out by acidic hydrolysis using strong acids such as HCl,^9^ TFA,^10^ or phosphoric acid.^11^ The removal of the Boc protecting group is one of the most frequently encountered transformations within the chemical and pharmaceutical community.^6−8^ However, the aforementioned deprotection methodology, utilizing acids, suffers from several limitations—selectivity/compatibility,^12,13^ excess amounts of reagent,^14,15^ aqueous workup,^15,16^ slurry formation due to vigorous off-gassing,^17,18^ and “foaming-out”.^13^ Consequently, the current methodology has been extended to include a variety of conditions, including use of Lewis acids,^19−21^ ionic liquids,^22^ deep eutectic liquid,^23^ Montmorillonite K10 clay,^24^ silica gel,^14^ base-mediated deprotection,^25,26^ and oxalyl chloride.^27^ Despite the range of conditions available for *N*-Boc deprotection many of these limitations persist.

Thermolytic *N*-Boc deprotections have been reported in batch using water^28,29^ and under microwave conditions in fluorinated solvents.^30^ However, temperatures greater than 100 °C are often required to achieve decarboxylation limiting the reaction solvent and setup. The use of continuous flow technology for performing organic transformations has achieved considerable attention in the last number of years with several reports focused on achieving thermolytic *N*-Boc deprotection on flow.^15,31,32^ Continuous flow technology offers several advantages for performing thermolytic reactions relative to batch conditions. The ability to heat a reaction above a solvent’s boiling point through integration of back-pressure regulators, superior heat transfer due to high surface area of continuous tubular reactors, and enhanced process control/safety through the incorporation of in-line reaction monitoring enables high-temperature chemistry to be achieved readily on a continuous flow system in comparison to batch reactors.

**Scheme 1:**
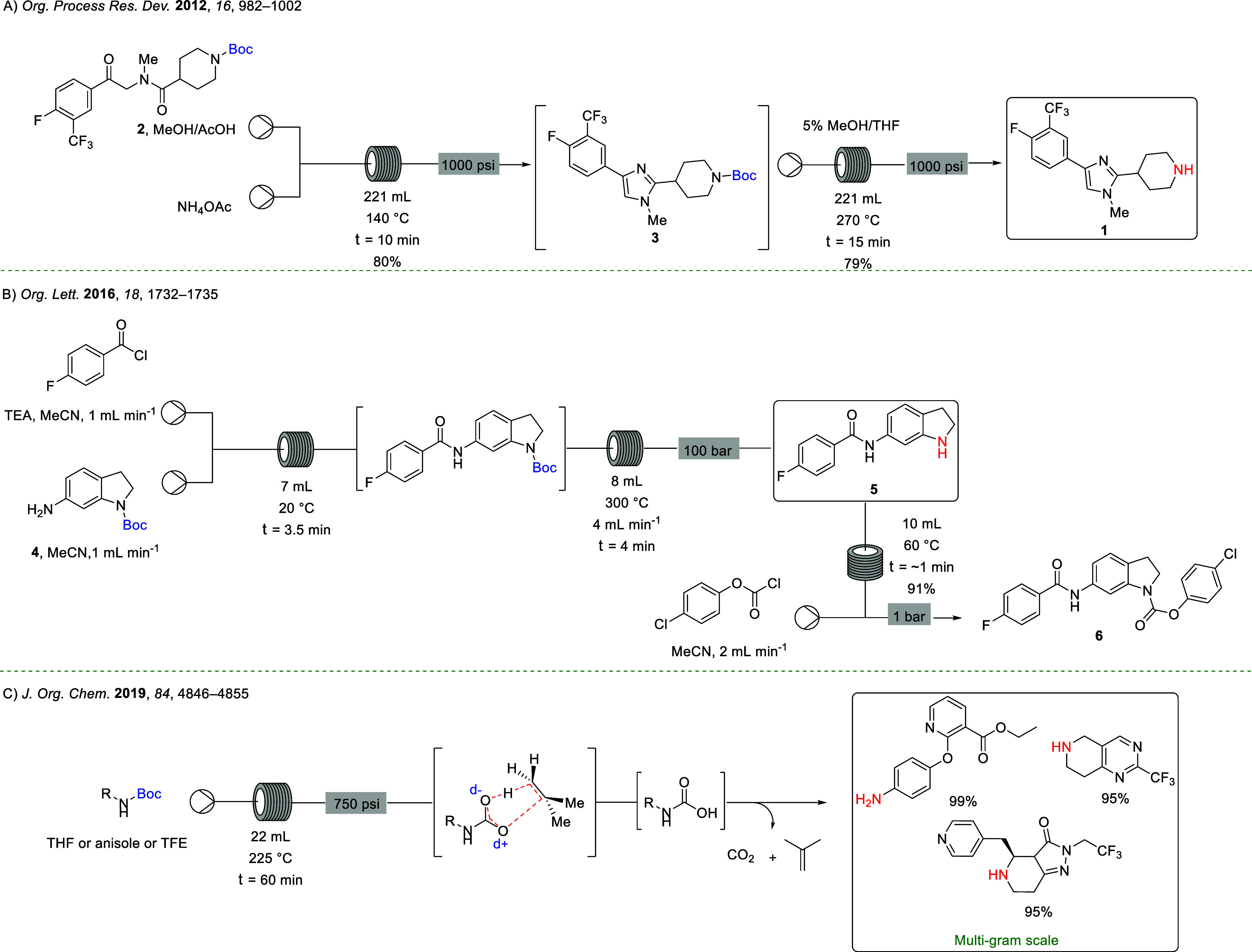
Thermal *N*-Boc Deprotection in Continuous Flow

May and co-workers have reported a second generation process for the synthesis of 1*H*-4-substituted imidazole **1** utilizing a plug flow reactor (PFR).^15^ The authors noted that while removal of the *N*-Boc group was achieved using 4 equiv of HCl in the first generation route they deemed the process “inefficient and wasteful”. The second generation route involved thermal cyclization of **2** to form intermediate **3** in 80% yield, followed by thermal deprotection of the *N*-Boc group under supercritical fluid conditions at 270 °C (Scheme 1A) to give imidazole **1** in 79% yield. This new route generated 1 kg of material over both PFR steps, and the developed route was equipped with automated sampling, dilution, and analysis.

Bogdan and co-workers demonstrated the versatility of a thermal deprotection process in multistep reaction sequences.^31^ A coupling-deprotection-coupling sequence was demonstrated using a high-temperature plug flow reactor, without the need for in-line extractions or workups. The coupling of an acid chloride with *N*-Boc amine **4** was achieved at room temperature with the reaction stream then injected into a second reactor coil and heated to 300 °C for thermal removal of the *N*-Boc group to produce the deprotected intermediate **5** (Scheme 1B). The reaction solution was then carried on to the next step, without isolation or aqueous workup, in a coupling reaction with carbamoyl chloride solution to form the final carbamate product **6**.

More recently, Li and co-workers have also reported using continuous flow technology for thermolytic removal of *N*-Boc groups from a selected range of substrates from the Pfizer compound library (Scheme 1C).^32^ The method was found to have broad functional group tolerance with substrates bearing ketones, amides, aryl and alkyl halides, ketals, nitriles, and esters among the functional groups to withstand the high-temperature deprotection conditions. High conversion (≥90%) was obtained for 12 out of the 26 compounds studied, and three substrates (Scheme 1C) gave ≥95% isolated yield on multigram scale. Mechanistic insight was attained through statistical and kinetic analysis and computational modeling.

One of the key challenges with the acid-catalyzed deprotection of *N*-Boc groups is poor selectivity relative to other acid-sensitive functionalities. In particular, the ability to selectively deprotect *N*-Boc groups (for example, distinguishing primary and secondary *N*-Boc amines or selective deprotection of aryl vs alkyl *N*-Boc derivatives) has been synthetically difficult using the traditional strong-acid methodology.^12,33^ However, if feasible, this would be attractive in the synthesis and functionalization of amine derivatives and would reduce the need for multiple orthogonal protecting groups. While there are some reports of 2° vs 1° *N*-Boc deprotection being achieved, this selectivity is currently limited to Boc groups on highly activated amines–indoles, pyrroles, or nitrogen in conjugation with a carbonyl or aromatic group.^25,26,34−36^ These methods suffer from long reaction times, use of harmful reagents, and tedious workup. Taking advantage of the enhanced process control possible in continuous flow, we envisaged that by careful control of the reaction temperature selective removal of a more labile *N*-Boc group may be possible in the presence of a less-reactive *N*-Boc group. This route would offer operational advantages as it would eliminate the need for an orthogonal protection methodology and potentially enable sequential transformations of the two amines without isolation or extractive workups.

## Results and Discussion

Initially a series of *N*-Boc protected amines were synthesized for use in preliminary investigations to explore conditions suitable for thermal deprotection on continuous flow. *N*-Boc amines **8a**–**n** were synthesized from commercially available free amine precursors (see Supporting Information for details of their synthesis), including a range of primary and secondary aliphatic and aromatic amines, heteroaryl amines, and amino acid precursors, to explore the impact of structural variation on the ease of thermal deprotection. Leveraging the enhanced process control available with flow reactor technology relative to traditional batch reactions, thermolytic deprotections of *N*-Boc amines **8a**–**n** were investigated in continuous flow to establish the feasibility and synthetic utility of the method, obviating the need for acidic reagents and, furthermore, to explore if selective deprotection of *N*-Boc groups could be achieved. Thermolytic deprotection reactions were conducted on a piston pump flow reactor using a stainless steel coil reactor (SCR) to enable temperatures up to 250 °C (in comparison to standard PFA reactor coils which can only withstand temperatures up to 150 °C). A key advantage in conducting the reactions in flow is the ability to heat solvents above their boiling points through use of back-pressure regulators (BPRs).^37−39^

**Scheme 2:**
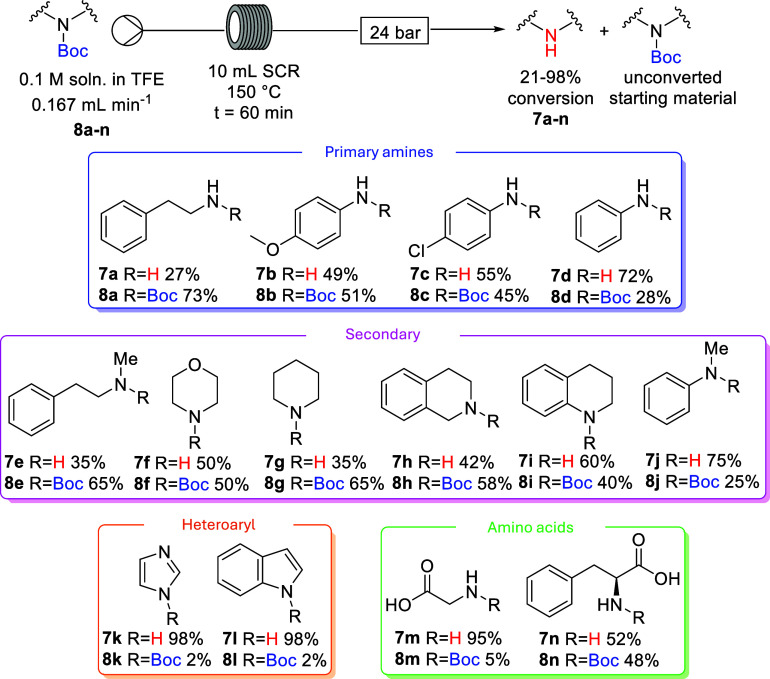
*N*-Boc Deprotection of Amine Precursors 8a–n Conversion determined by ^1^H NMR spectroscopy using CDCl_3_ as solvent.

As summarized in Scheme 2, the initial studies demonstrated that *N*-Boc deprotection proceeds well under thermolytic conditions using trifluoroethanol (TFE) as the reaction solvent at 150 °C and a 60 min residence time,^30−32^ with the outcome of the reaction monitored by ^1^H NMR spectroscopy. Excellent conversion of the most reactive derivatives, *N*-Boc imidazole **8k** and *N*-Boc indole **8l**, to the deprotected amines **7k** and **7l** was observed (98% in each case). As anticipated, the efficiency of deprotection of *N*-Boc aryl amines **8b**–**d** and **8i** and **8j** (49–72%) was greater than that of *N*-Boc alkyl amines **8a**, **8e**–**h** (27–50%). In general, deprotection of secondary *N*-Boc amines was more efficient than that of the comparable primary *N*-Boc amines; for example, deprotection of *N*-Boc methyl-phenethylamine **8e** is more efficient than deprotection of *N*-Boc phenethylamine **8a** (35% cf. 27%), while deprotection of the *N*-Boc derivatives of cyclic secondary amines **8f**–**h** (35–60%) was slightly more efficient than that of acyclic derivative **8e** (27%). The deprotection of secondary aryl *N*-Boc derivatives **8i** and **8j** (60% and 75%) is only marginally more efficient than that of primary aryl *N*-Boc derivatives **8b**–**d** (49–72%). Deprotected morpholine (**7f**) was obtained with higher conversion than that of the deprotection to obtain free piperidine (**7g**) (50% cf. 35%). The deprotection of *N*-Boc glycine **8m** (95%) was more efficient than the deprotection of alkyl primary *N*-Boc amines **8a** and **8e**–**2h** (27–50%), as anticipated, as the role of a free carboxylate has been shown to increase the efficiency of Boc deprotection for amino acids.^40^ Deprotection of *N*-Boc glycine **8m** was more efficient than that of *N*-Boc phenylalanine **8n** (95% cf. 52%). As anticipated based on the ease of deprotection under standard acidic conditions, the efficiencies of thermal deprotection in flow follow the sequence *N*-Boc heteroaryl > *N*-Boc aryl > *N*-Boc alkyl amines, in line with p*K*_a_ values for the conjugate acids.^21,41,42^ Differentiation between primary and secondary alkyl amines is less appreciable, but, nonetheless, these initial results have confirmed that there is a link between the amine structure and the ease of thermal deprotection on continuous flow, potentially leading to selective deprotection.

**Figure 1:**
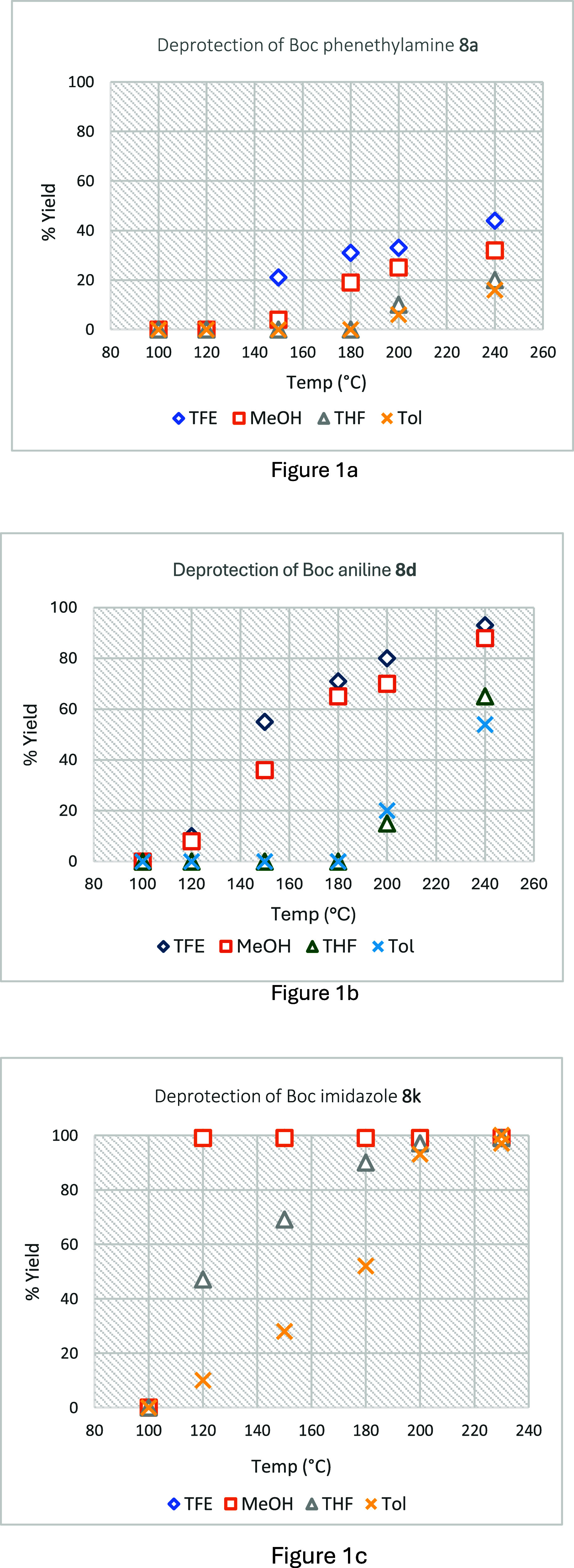
Impact of variation of temperature on the efficiency of the thermal deprotection of Boc derivatives **8a** (Figure 1a), **8d** (Figure 1b), and **8k** (Figure 1c).

The next step was to explore how variation in reaction conditions, such as the reaction solvent, temperature, and residence time, would affect the efficiency of the deprotection. *N*-Boc phenethylamine **8a**, *N*-Boc aniline **8d**, and *N*-Boc imidazole **8k** were selected to systematically study these effects in flow. Four reaction solvents were investigated, trifluoroethanol (TFE), methanol, tetrahydrofuran, and toluene, and temperatures ranging from 100–240 °C, with the results summarized in Figure 1a–c.

The deprotection reactions of *N*-Boc imidazole **8k** to free imidazole **7k** were highly efficient in TFE and MeOH, with 100% yield obtained at temperatures starting at 120 °C for a residence time of 30 min to enable comparison of efficiencies (Figure 1c). However, reactions were less efficient in THF (47%), and no deprotected product was obtained in toluene at the same temperature. To achieve comparable efficiencies in THF or toluene, to those in TFE or MeOH, the reaction temperature needed to be at 200 °C or higher (97% cf. 93% cf. 99% cf. 99% yields). Comparable thermal deprotection data were collected for *N*-Boc aniline **8d**; once again, using 30 min reaction times *N*-Boc deprotection was most efficient when performed in TFE and MeOH (Figure 1b). However, a temperature of 240 °C was needed to achieve efficient deprotection (93% in TFE, 88% in MeOH). At the same temperature, only 65% deprotected aniline was obtained in THF and 54% in toluene, following 30 min of residence time. The thermal deprotection reaction was least efficient for the deprotection of the alkyl amine, *N*-Boc phenethylamine **8a**. Even at a high temperature of 240 °C, the reaction efficiency was poor in each of the solvents studied, with highest yield of deprotected phenethylamine (**7a**) obtained in TFE with a 30 min residence time (44%, Figure 1a). Longer residence times were needed in each of the solvents to achieve good yields of deprotected phenethylamine (**7a**).

**Figure 2:**
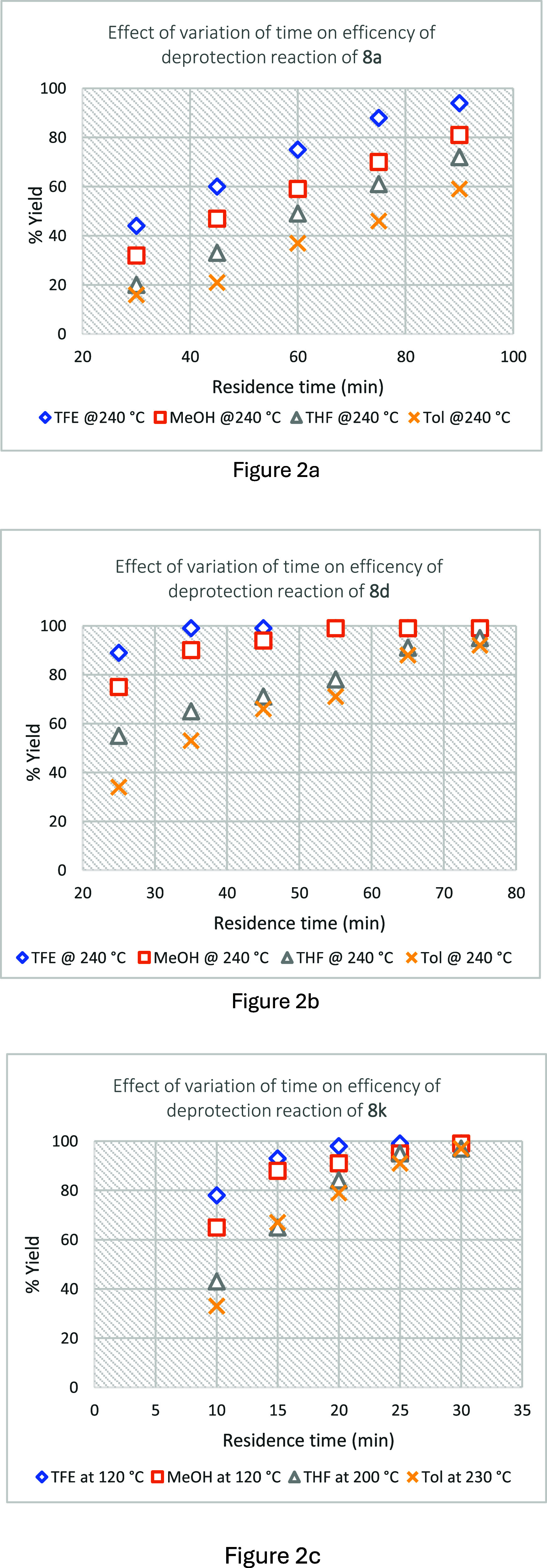
Impact of variation of residence time on the efficiency of the thermal deprotection of Boc derivatives **8a** (Figure 2a), **8d** (Figure 2b), and **8k** (Figure 2c).

The impact of variation of residence time on the efficiency of the thermal deprotection was investigated, as illustrated in Figure 2a–c. Deprotections of *N*-Boc imidazole **8k** in each solvent were conducted at the lowest temperature, which gave complete deprotection in 30 min as shown in Figure 2c. A 20 min residence time was required to achieve complete deprotection in TFE at 120 °C, while a 25 min residence time was required for complete reaction in methanol at 120 °C, and 30 min in THF at 200 °C and in toluene at 230 °C. Based on the results shown in Figure 1b, the investigation of the impact of variation of residence time on the deprotection efficiency of *N*-Boc aniline **8d** was undertaken at 240 °C (Figure 2b). A 35 min residence time was required to achieve complete deprotection in TFE and MeOH, whereas this residence time in THF and toluene resulted in only 71% and 66% deprotected aniline (**7d**), although essentially complete deprotection could be achieved by extended residence times (>70 min). A 90 min residence time led to 94% deprotected phenethylamine (**7a**) in TFE, 81% in MeOH, 72% in THF, and 59% in toluene, all conducted at 240 °C (Figure 2a).

Looking at the data overall, it is evident that by tuning the reaction variables of solvent, temperature, and residence time the yields of deprotected amines can be optimized. For example, when TFE was used as reaction solvent the conditions needed to obtain >94% of deprotected imidazole (**7k**), aniline (**7d**), and phenethylamine (**7a**) were 25 min at 120 °C, 35 min at 240 °C, and 90 min at 240 °C, respectively. One of the key advantages of conducting the reactions in flow is the ability to achieve reaction temperatures above the boiling point of the solvents; under standard batch conditions the efficiencies of the thermal deprotections at the boiling point of each solvent would not be synthetically feasible.

It is evident that the efficiency and rate of thermal deprotection in flow is greater in polar protic solvents such as TFE and MeOH than in polar aprotic THF and nonpolar aprotic toluene. The increased efficiency in TFE relative to MeOH correlates with the increased acidity of TFE (p*K*_a_ 12.46) relative to MeOH (p*K*_a_ 15.5).^43^

**Figure 3:**
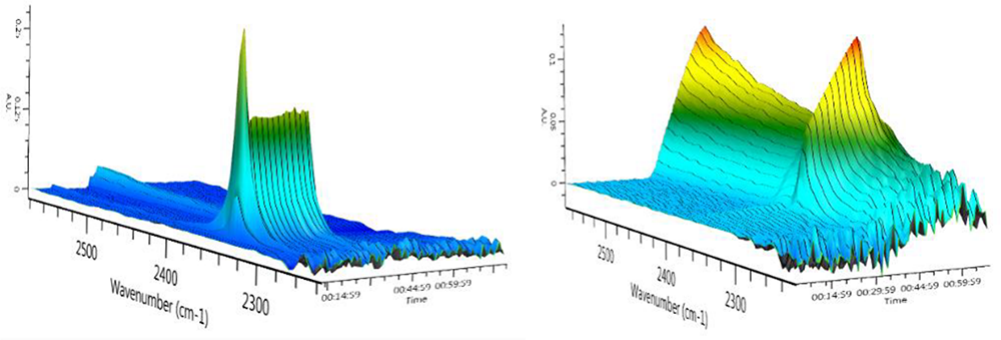
IR spectrum of reaction outflow from *N*-Boc imidazole **8k** deprotection in MeOH (left) and THF (right).

The progress of the thermolytic *N*-Boc deprotection is readily followed by FlowIR as illustrated in Figure 3, with very rapid evolution of CO_2_ from the reaction of **8k** at 150 °C in MeOH; interestingly, the rate of evolution of CO_2_ was slower when the reaction was conducted in THF. Use of real-time FlowIR monitoring offers a clear advantage in terms of controlling reaction time/temperature and moderating the evolution of CO_2_.

**Figure 4:**
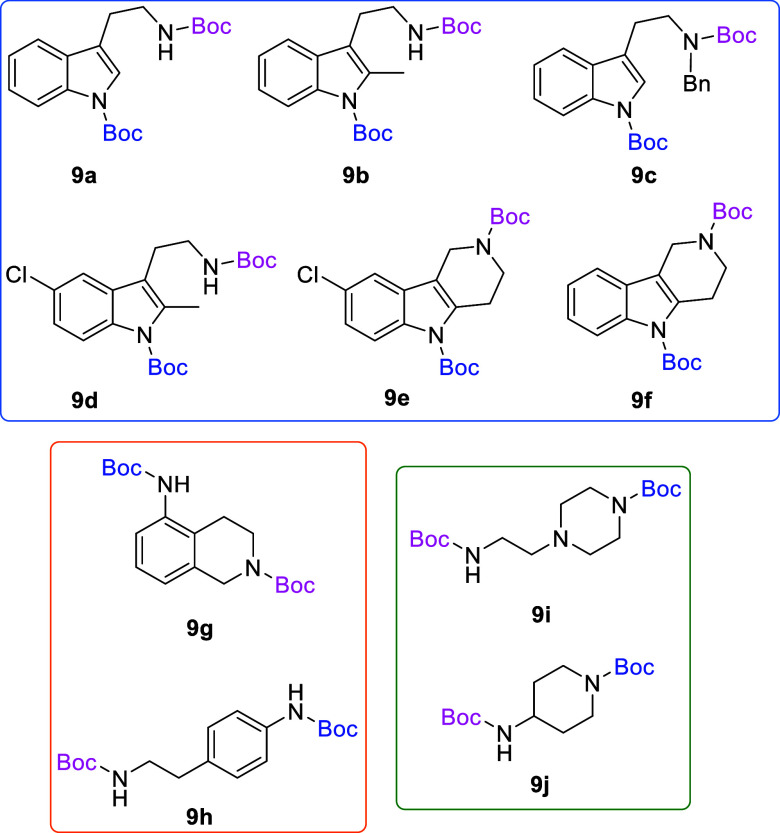
Substrates selected to explore selectivity of thermal *N*-Boc deprotection in flow.

Based on the results above, selective deprotection of doubly protected *N*-Boc diamines was explored to establish if one amine could be deprotected while leaving the second *N*-Boc group unaffected based on the difference in reactivity. The substrates shown in Figure 4 were chosen to investigate selective deprotection (see Supporting Information for details of their synthesis). For tryptamine and carboline derivatives **9a**–**f** selective removal of the aryl *N*-Boc group was anticipated, ideally leaving the alkyl *N*-Boc groups unaffected. Substrates **9g**–**h** were designed to investigate selective deprotection of aryl *N*-Boc groups in the presence of alkyl *N*-Boc groups, while *N*-Boc diamines **9i** and **9j** were chosen as model substrates to explore selective deprotection of a 2° aliphatic *N*-Boc amine in the presence of a 1° aliphatic *N*-Boc amine.

While both MeOH and TFE proved effective in the model *N*-Boc deprotections, for the selective deprotections investigations were conducted in MeOH, which is more attractive as a process solvent.^44^ Each of the bis-Boc tryptamines **9a**–**f** was dissolved in methanol, and this solution was pumped through a stainless steel coil reactor at 150 °C for a residence time of 30 min to explore selective deprotection (Table 1, Entries 1, 3, 5, 7, 9, 12). The reaction outflow was collected in each case and concentrated, and ^1^H NMR spectra were recorded to determine the extent of deprotection to the mono-Boc derivatives **10a**–**f** and to check for overdeprotection to **11a**–**f**. Excellent conversion to mono-Boc diamines **10a**–**f** (88–93%) was observed in the spectra of the crude reaction products, with no evidence for complete deprotection to the diamines **11a**–**f**. The only other significant component in the mixtures was residual starting bis-Boc diamines **9a**–**f** (<12%). After column chromatography, the isolated mono-Boc derivatives were obtained in pure form and excellent yields, and the spectral data agreed with the literature.

**Table 1:**
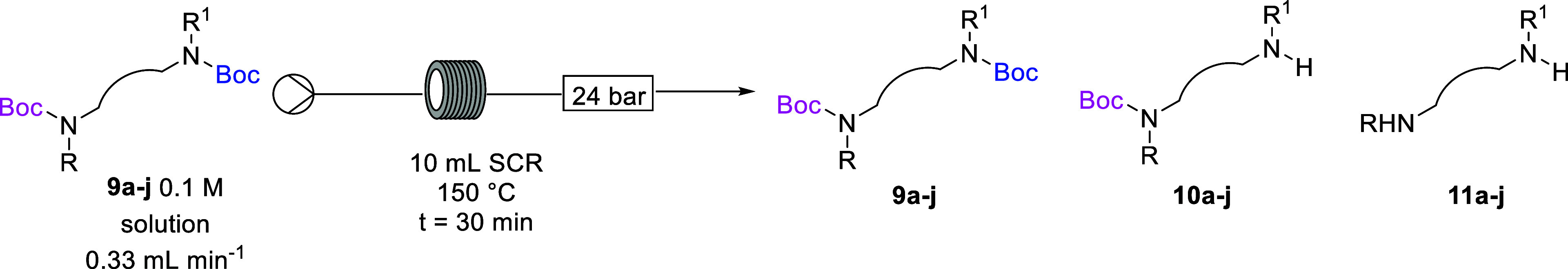
Continuous Flow Thermal Deprotection of bis-Boc Diamines **9a**–**j**

					**Crude product ratio**^a^**(isolated yields**^b^**)**		
**Entry**	**Compound**	**Solvent**	**Temp (°C)**	**Time (min)**	**Bis-Boc Diamine 9a-j**	**Mono-Boc Diamine 10a-j**	**Free Diamine 11a-j**
1	**9a**	MeOH	150	30	9	91 (90)	0
2^c^	**9a**	MeOH	230	45			95 (90)
3	**9b**	MeOH	150	30	7	93 (88)	0
4	**9b**	MeOH	230	45		6	88 (85)
5	**9c**	MeOH	150	30	8	92 (82)	0
6^c^	**9c**	MeOH	230	45		4	87 (82)
7	**9d**	MeOH	150	30	12	88 (83)	0
8^c^	**9d**	MeOH	230	45		5	85 (73)
9^c^	**9e**	MeOH	150	30	8	88 (80)	0
10^c^	**9e**	MeOH	230	45			60 (−)
11^c^	**9e**	TFE	180	50		8	67 (55)
12	**9f**	MeOH	150	30	9	91 (86)	0
13^c^	**9f**	MeOH	230	45			65(−)
14^c^	**9f**	TFE	180	50			80 (70)
15	**9g**	MeOH	170	90	35	57 (43)	8
16	**9g**	MeOH	240	45		10	81(69)
17	**9h**	MeOH	170	65	40	54(41)	6
18^c^	**9h**	MeOH	220	35		10	85(70)
19^c^	**9h**	MeCN	230	10	15	85(70)	0
20^c^	**9i**	MeOH	150	45	70	15(10)	10
21^c^	**9j**	MeOH	130	80	40	20(12)	40

a Determined by ^1^H NMR spectroscopic analysis.

b After column chromatography on silica gel.

c Unidentified components also observed in the ^1^H NMR spectra.

Having established that *N*-Boc diamines **9a**–**f** could be selectivity deprotected at 150 °C to remove the aryl *N*-Boc group, more forcing conditions (180–240 °C) were then employed with the objective of effecting complete deprotection to afford the free diamines **11a**–**d** (Table 1, Entries 2, 4, 6, 8). By heating the bis-Boc substrates **9a**–**d** in a methanol solution at 230 °C for a 45 min residence time, deprotected tryptamines **9a**–**d** were isolated after chromatography in good yields (73–90%). The ^1^H NMR spectra recorded for the crude reaction material in each case showed good conversions to the free diamine **11a**–**d**, containing small amounts of the bis-Boc starting material. Increasing the residence time resulted in a negligible increase to deprotected product but increased the presence of unidentified signals in the ^1^H NMR spectrum of the reaction mixture.

When bis-Boc carboline derivatives **9e** and **9f** were heated to 230 °C in MeOH, pressure increases and solid precipitate were observed in the flow system. As the free carbolines **11e** and **11f** had low solubility in MeOH at 0.1 M concentration the reactions were conducted instead at 0.05 M. The reaction proceeded at 230 °C, but there was significant impurity formation evident from the resulting ^1^H NMR spectra of the crude reaction material, suggesting the thermal degradation of one or more of the components in the reaction mixture. The free carbolines **11e** and **11f** had better solubility in TFE, which was subsequently used as solvent; furthermore, use of TFE enabled a lower reaction temperature of 180 °C for a 60 min residence time leading to the free diamines **11e** and **11f** with less degradation than seen in MeOH and in good isolated yields after chromatography (55% and 70%) (Table 1, Entries 11 and 14).

**Scheme 3:**
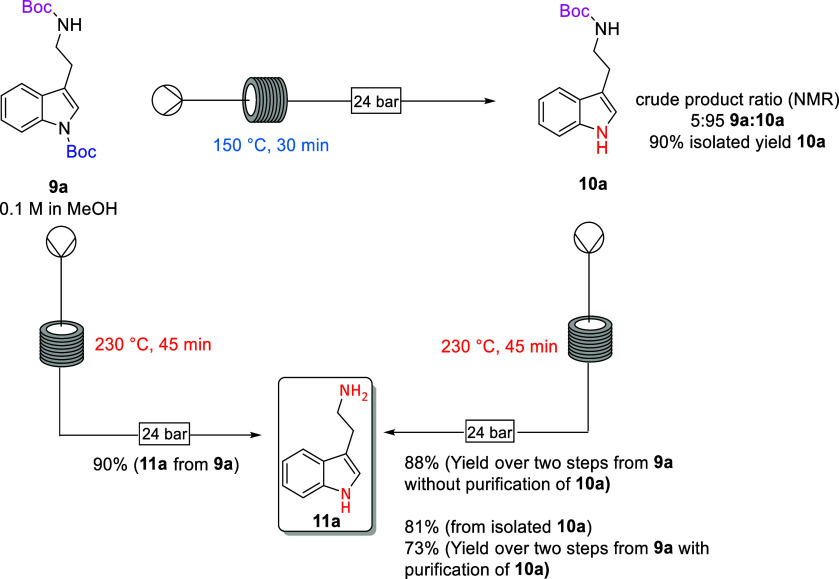
Continuous Flow Deprotection of *N*-Boc Tryptamine **9a**

Scheme 3 illustrates the versatility of the stepwise selective deprotection method on continuous flow. Deprotection of the aryl *N*-Boc group can be selectively achieved via control of reaction temperature and residence time to obtain mono-Boc tryptamine **10a** with 90% isolated yield with no evidence of formation of **11a**. The free tryptamine **11a** was then obtained in 81% yield (73% overall from **9a**) by heating the isolated mono-Boc tryptamine **10a** to a higher temperature of 230 °C and slightly longer residence time of 45 min. Alternatively, the removal of both Boc groups can be achieved in a single step in 90% yield by heating to 230 °C for 45 min.

While selective deprotection of the tryptamines was very effective under thermolytic conditions, the selectivity of deprotection of the other bis-Boc diamine derivatives **9g** and **9h** was less. While the major product was due to loss of the aryl *N*-Boc group, there was also some starting material **9g** and **9h** and fully deprotected product **11g** and **11h** present in the crude reaction mixtures from the lower-temperature deprotections at 170 °C (Table 1, Entries 15 and 17). Potentially the efficiency of these selective deprotections could be optimized through alteration of reaction temperature and residence time. Distinguishing between *N*-Boc groups on primary and secondary aliphatic amines was, unsurprisingly, even more challenging (Table 1, Entries 20 and 21).

**Scheme 4:**
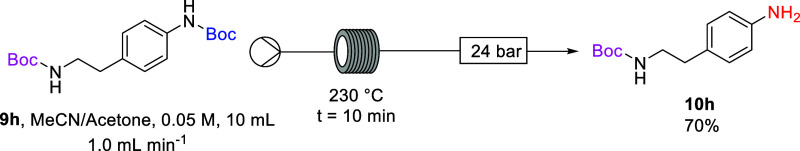
Selective Deprotection of bis-Boc Diamine 9h

Bis-Boc diamine **9h** was chosen as a model substrate to explore the thermal flow *N*-Boc deprotection and telescoped functionalization of the free amine. Benzoylation of the aryl amine was undertaken to exemplify the potential for selective reaction of one of the amine groups between the two Boc deprotections. First, the selective thermal deprotection of diamine **9h** was performed thermolytically on flow (Table 1, Entry 19) as illustrated in Scheme 4, successfully affording the deprotected mono-Boc amine **10h** with 85% conversion and in a 70% isolated yield following chromatography.

Having established that the model substrate **9h** could be selectively deprotected under thermal flow conditions exploration of its benzoylation was next undertaken, initially in batch. A pure sample of mono-Boc amine **10h** was initially benzoylated using 1 equiv of triethylamine as base and dropwise addition of 1 equiv benzoyl chloride in a 7:3 mixture of acetonitrile and acetone, to ensure the same solvent system used as the thermal flow deprotection step (Table 2, Entry 1). While the amide **12** was formed in quantitative yield of 99%, small amounts of precipitate were observed upon the addition of the benzoyl chloride (presumably the HCl salt of **10h**) that eventually dissolved after a few minutes of stirring. Increasing the equivalents of base avoided this precipitation (Table 2, Entry 3). Due to the potential advantages of an immobilized base in the telescoped process, use of Amberlyst A21 for the reaction was investigated, once again leading to quantitative yield of amide **12** (Table 2, Entry 4, 98%).

**Table 2:**
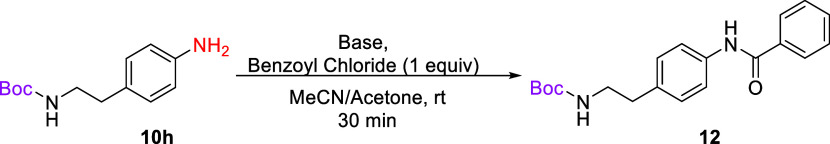
Reaction Conditions for Benzoylation of mono-Boc Amine **4h**

**Entry**	**Base**	**Base (equiv)**	**Yield (%)**
1	NEt_3_	1.0	99^a^
2	NEt_3_	1.5	100^a^
3	NEt_3_	2	100
4	Amberlyst A21 resin	1.5	98

a Precipitate observed formed on addition of benzoyl chloride.

Following the successful benzoylation of amine **10h** in batch, the next step was to transfer the benzoylation step to a continuous flow system. As previously mentioned, the precipitation of the HCl salt observed in the batch benzoylation reactions is not ideal for a continuous flow setup. As such, conditions described in Table 2, Entries 3 and 4, were employed for investigation in continuous flow as no precipitate was observed under these conditions.

**Scheme 5:**
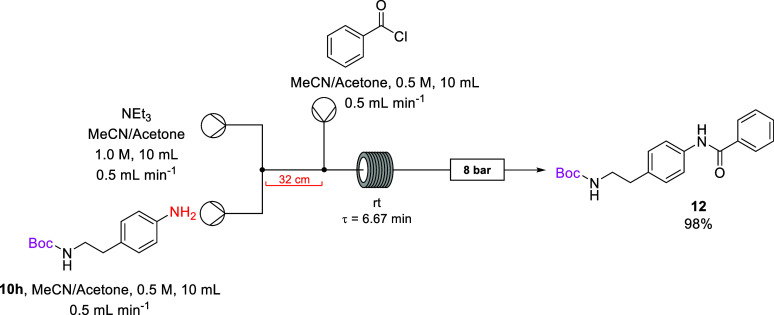
Continuous Flow Generation of Amide **12**

The flow setup consisted of three reaction solutions—a solution of triethylamine in 7:3 acetonitrile/acetone, a solution of mono-Boc **10h** in 7:3 acetonitrile/acetone, and a solution of benzoyl chloride in 7:3 acetonitrile/acetone. Under batch conditions mono-Boc amine **10h** was stirred with triethylamine as base before the addition of benzoyl chloride. This was replicated under continuous flow by having the mono-Boc amine **10h** solution and base solution meet at a T-piece and travel along a 32 cm piece of tubing before meeting the solution of benzoyl chloride at another T-piece. The premixing of the amine **10h** and the base was undertaken to prevent blockages within the reactor tubing. The combined reaction solution was then pumped into a PFA reactor coil for a residence time of 6.67 min before passing through an 8 bar BPR, and the reaction outflow was subsequently collected. There were no residues visible in the reactor lines indicating that the material had passed efficiently through the lines and into the reactor coil (Scheme 5).

Full conversion to the amide **12** was observed by ^1^H NMR spectroscopy of the crude reaction mixture after solvent evaporation and aqueous workup. The amide **12** was subsequently isolated in excellent 98% yield as a white solid that could be stored at room temperature without degradation. Use of Amberlyst 21 as an immobilized base proved less successful with blockages due to precipitation prior to contact between the solutions and the base.

**Scheme 6:**
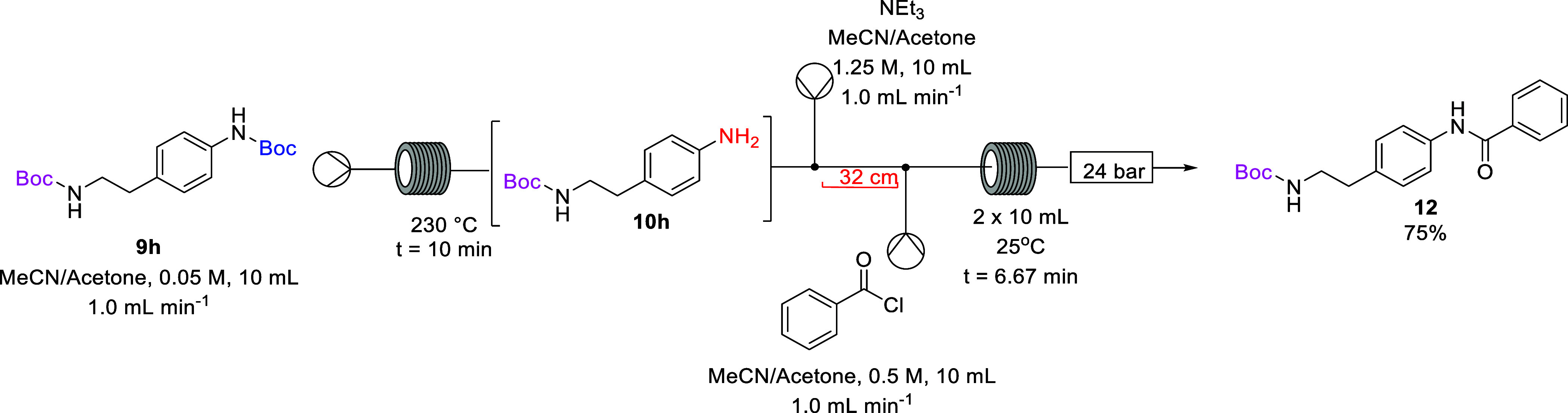
Telescoped Selective *N*-Boc Deprotection and Benzoylation of **9h**

Having demonstrated the successful synthesis of amide **12** on continuous flow, the next step was to telescope the selective thermal deprotection and the benzoylation (Scheme 6). The bis-Boc diamine **9h** in the acetonitrile/acetone solution (7:3) was pumped through a stainless steel coil reactor which was preheated to 230 °C for a residence time of 10 min. The reaction outflow was then pumped forward to a T-piece where it met a solution of triethylamine. The combined reaction stream was pumped through a 32 cm piece of PFA tubing before meeting the benzoyl chloride solution at a T-piece, and the combined reaction streams passed through 2 × 10 mL PFA reactors set at 25 °C for a residence time of 6.67 min and then a series of 3 × 8 bar back-pressure regulators.

**Scheme 7:**
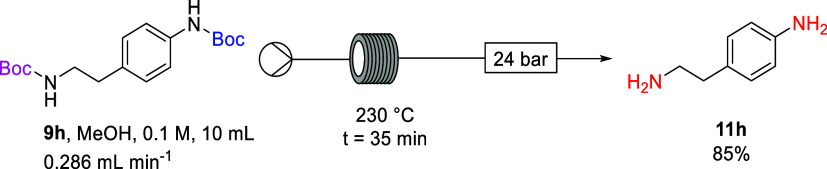
Deprotection of Alkyl and Aryl *N-*Boc Groups of **9h**

Having established selective thermal deprotection and benzoylation of **9h** to form amide **12**, attention was next focused on thermal deprotection of the less-reactive *N*-Boc group based on the original observation of complete thermal deprotection of **9h** to form **11h** (Scheme 7 and Table 1, Entry 18). Thus, deprotection of the alkyl *N*-Boc group on bis-Boc diamine **9h** can be achieved by heating a solution of the compound in MeOH under thermal continuous flow conditions. We anticipated that under similar thermal continuous flow conditions the alkyl *N*-Boc group on amine **9h** could be removed without affecting the amide functionality on amide **12**.

Initially a pure sample of amide **12** in MeOH (Table 3, Entry 8) at 230 °C was used to explore if similar temperature and residence time conditions employed for **9h** would be sufficient for alkyl *N*-Boc deprotection, leading to 70% isolated yield of **13**, while a 75% yield was obtained in 1:1 MeOH/acetone with improved solubility throughout the process. However, to telescope the second deprotection with the earlier sequence from **9h** to **10h** to **12**, use of MeCN/acetone would be required.

**Table 3:**
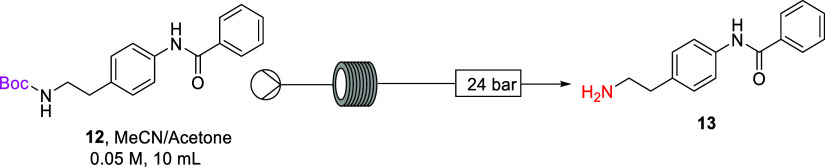
Thermal Continuous Flow *N*-Boc Deprotection Amide **12**

**Entry**	**Solvent**	**Temp (°C)**	**Residence time (min)**	**Yield**^a^^,^^b^**(%)**
1	7:3 MeCN/acetone	230	15	15
2	7:3 MeCN/acetone	230	30	39
3	7:3 MeCN/acetone	230	60	43
4	1:1 MeCN/acetone	230	30	45
5	1:1 MeCN/acetone	245	30	21^c^
6	1:1 MeCN/acetone	180	40	28
7	1:1 MeCN/acetone	180	60	34
8	MeOH	230	30	70^d^
9	1:1MeOH/acetone	230	30	77^d^

a Isolated yield after column chromatography on silica gel.

b The crude product material was mainly recovered starting material **12**, with other fractions that were isolated being unidentified from their ^1^H NMR spectra.

c Minor fraction of diamine **11h** was obtained.

d The crude product material was mainly amine **13**, with other fractions that were isolated being unidentified from their ^1^H NMR spectra.

In this solvent mixture when the same temperature and residence time were employed for the deprotection of the alkyl *N*-Boc group on amide **12**, the yield obtained of the free amine **13** was only 15% (Table 3, Entry 1), while increasing the residence time to 30 min led to an increased yield 39% (Entry 2). However, it should be noted that the reaction outflow was dark amber/brown in color and precipitation was observed in the reactor tubing. In addition, signals not attributed to starting material or product were observed in the ^1^H NMR spectra of the crude reaction material. Increasing the residence time to 60 min (Entry 3) only marginally increased the yield of free amine **13** (43%). The solubility of the free amine **13** was poor in the 7:3 MeCN/acetone solvent system, so a 1:1 MeCN/acetone solvent system was subsequently used (Table 3, Entries 4–7). The reactor temperature and residence time were varied in attempts to increase the yield of free amine **13**. Increasing the reactor temperature to 245 °C gave a 21% yield of **13**, but recovery of free diamine **11h** was also obtained in minor amounts (∼5%, Entry 5). Conducting the reaction at lower temperature of 180 °C and 40 min residence time gave moderate isolated yield and less impurities observed in the ^1^H NMR spectrum of the crude reaction material (Entry 6, 28%); however, increasing the residence time only gave a small increase in the free amine **13** (Entry 7, 34%).

**Scheme 8:**
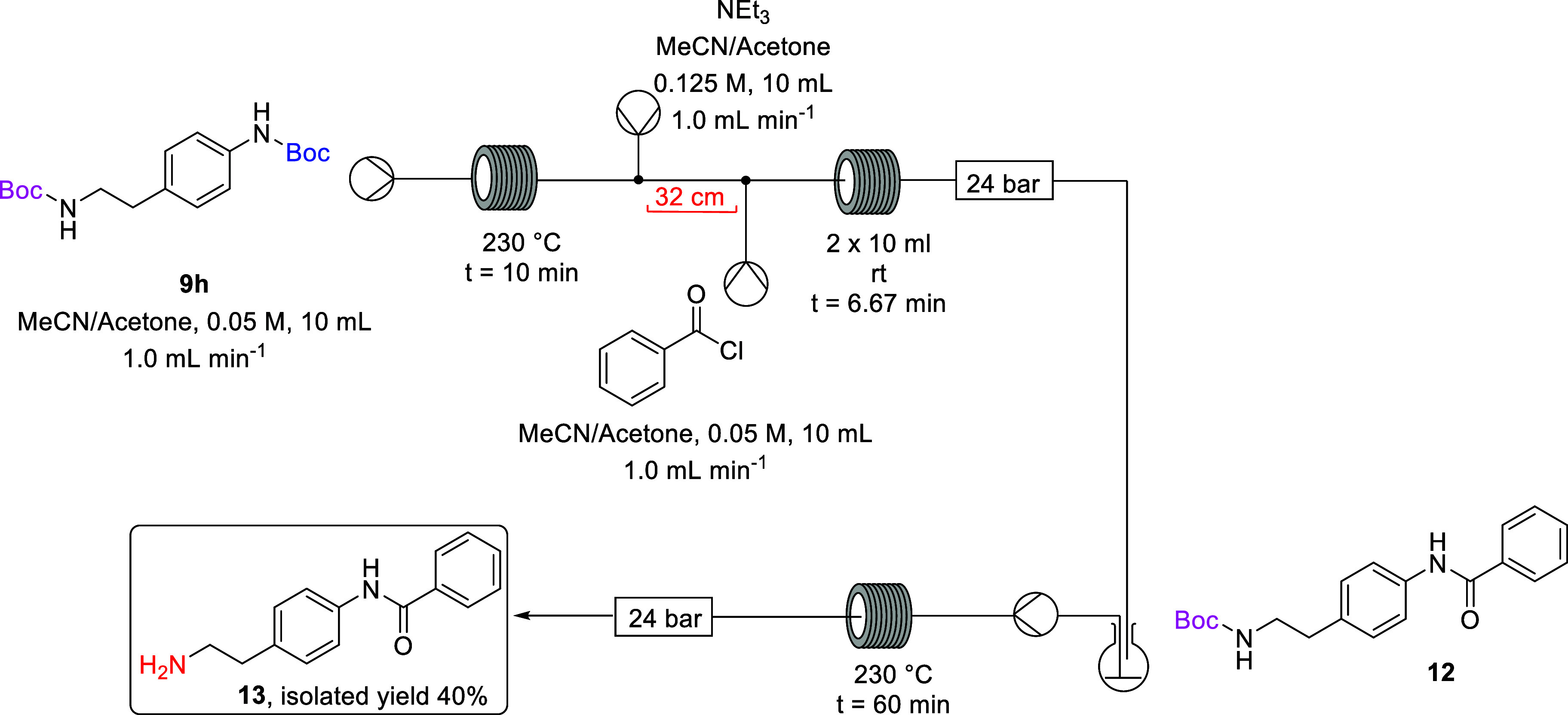
Telescoped Selective Deprotection and Benzoylation of **9h**

With these results in hand, telescoping the alkyl *N*-Boc deprotection step with the selective aryl *N*-Boc deprotection and aryl amine benzoylation steps was next explored. The selective thermal deprotection of bis-Boc diamine **9h** and benzoylation step were carried out as previously demonstrated in Scheme 4 and then telescoped with the conditions employed in Table 3, Entry 3 (Scheme 8). Access to only one stainless steel coil reactor meant that following the selective deprotection and benzoylation step the reaction outflow was temporarily collected in a round-bottom flask, without isolation or workup of the reaction material. The stainless steel coil reactor was flushed briefly with reaction solvent (7:3 MeCN/acetone) and reconfigured, and the collected reaction solution containing amide **12** was subsequently pumped through the preheated coil at 230 °C for a residence time of 60 min to afford free amine **13** in a 40% isolated yield overall from **9h**. However, operationally there were challenges due to precipitation throughout the final step.

**Scheme 9:**
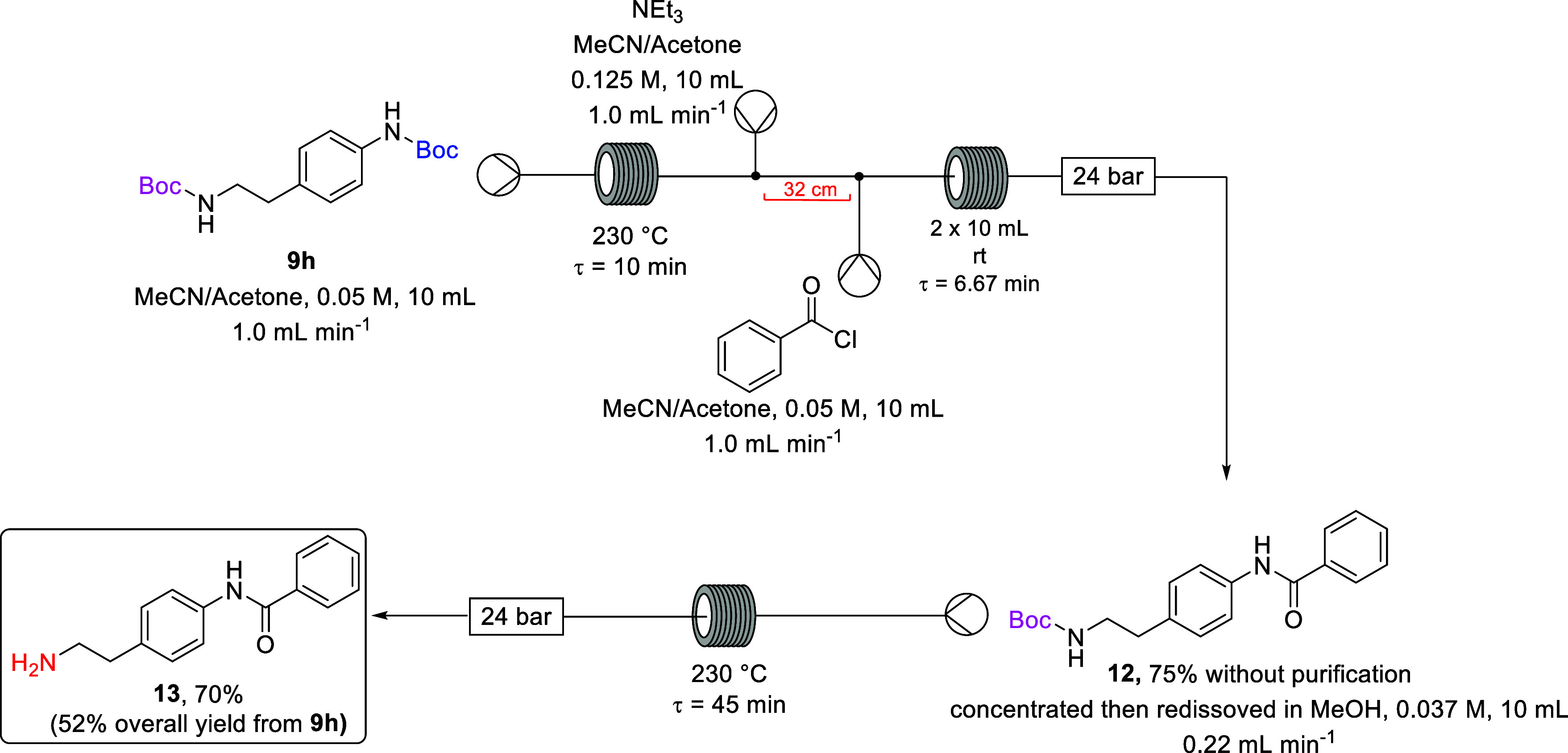
Telescoped Selective Deprotection and Benzoylation Utilizing Solvent Swap

As the deprotection of the alkyl *N*-Boc group on amide **12** was more efficient in MeOH or MeOH/acetone as a reaction solvent (Table 3, Entry 8 and 9), a solvent swap after the benzoylation step was embedded in the telescoped deprotection sequence (Scheme 9). After the selective deprotection and benzoylation step the reaction outflow was collected in a round-bottom flask and concentrated by rotary evaporation to yield the crude amide **12** (75% crude yield). No purification was undertaken, and the crude residue was dissolved in a 1:1 MeOH/acetone mixture. The stainless steel coil reactor was flushed with reaction solvent (1:1 MeOH/acetone mixture) and reconfigured, and the reaction solution containing amide **12** was subsequently pumped through the preheated coil at 230 °C for a residence time of 30 min to afford free primary amine **13** in a 70% isolated yield (52% overall from **9h**).

There are limited reports of selective *N*-Boc group deprotection under acidic conditions. To compare the synthetic utility of the thermal Boc deprotection in flow investigation of selective acid-mediated deprotection **9h** was undertaken using TFA. Despite variation of the number equivalents of TFA, clean deprotection to **10h** was not achieved, and reaction times were much longer.

In conclusion, thermal *N*-Boc deprotection of a range of amines is readily effected in continuous flow, in the absence of an acid catalyst. While the optimum results were obtained in methanol or trifluoroethanol, deprotection can be effected in a range of solvents of different polarities. Sequential selective deprotection of *N*-Boc groups has been demonstrated through temperature control, as exemplified by effective removal of an aryl *N*-Boc group in the presence of an alkyl *N*-Boc group. As a proof of principle, a telescoped sequence involving selective deprotection of an aryl *N*-Boc group from **9h** followed by benzoylation and deprotection of the remaining alkyl *N*-Boc group to form the amino amide **13** proved successful.

## Experimental Section

### Preparation of Bis-Boc Diamines **9c**–**g**

*General procedure A:* Di-*tert*-butyl-dicarbonate (1.1 equiv) was added under nitrogen to a stirring solution of appropriate diamine (1.0 equiv) and triethylamine (1.0 equiv) in acetonitrile at 0 °C. The reaction mixture was stirred for 1 h, with TLC monitoring, followed by a second addition of di-*tert*-butyl-dicarbonate (1.1 equiv) and DMAP (10 mol %) in acetonitrile. The reaction mixture was stirred under nitrogen for 12 h, after which the mixture was washed with aqueous HCl (2 M, 1 × 10 mL/mmol), water (1 × 10 mL/mmol), and brine (1 × 15 mL/mmol), dried with Na_2_SO_4_, filtered, and concentrated under reduced pressure to afford the crude *N,N*′-di-Boc-amine. Chromatographic purification in some instances was undertaken, as detailed below.^45^

### *tert*-Butyl-3-(2-(benzyl(*tert*-butoxycarbonyl)amino)ethyl)-1*H*-indole-1-carboxylate (**9c**)


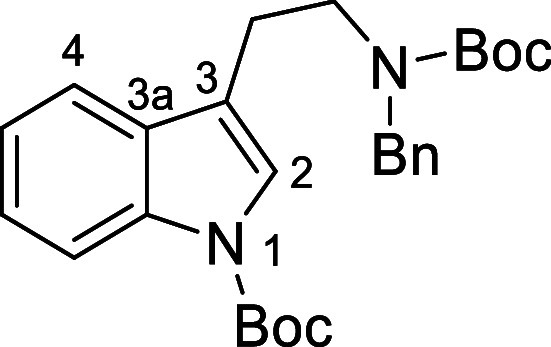
The title compound was prepared according to the *General Procedure A* using *N*-benzyl-2-(1*H*-indol-3-yl)ethan-1-amine (0.86 g, 3.45 mmol, 1.0 equiv), di-*tert*-butyl-dicarbonate (2 × 0.80 g, 7.36 mmol, 2.2 equiv), DMAP (0.08 g, 0.34 mmol, 0.1 equiv), and triethylamine (0.33 g, 3.45 mmol, 1.0 equiv) in acetonitrile (50 mL) at 0 °C. The crude product was purified by flash chromatography on silica gel using hexane/ethyl acetate (90:10) affording the pure bis-Boc compound **9c** as a pale yellow oil (1.55 g, 75%): ν_max_/cm^–1^ (ATR) 1694 (C=O), 1122 (C–O). δ_H_ (400 MHz, CDCl_3_): 1.46 [9H, s, NHCOC(C*H*_3_)_3_], 1.65 [9H, s, NCOC(C*H*_3_)_3_], 2.75–2.93 [2H, br m, C*H*_2_], 3.39–3.58 [2H, br m, NC*H*_2_], 4.30–4.53 [2H, br m, benzyl C*H*_2_], 7.12–7.51 [9H, br m, aromatic 9 × C*H*, overlapping with CHCl_3_ peak], 8.09–8.15 [1H, m, aromatic C*H*]. δ_c_ (100 MHz, CDCl_3_, evidence of 2 rotamers in approximately equal molar amounts for some signals): 23.7, 24.1 [CH_2_, *C*H_2_], 28.24, 28.45 [CH_3_, NHCOC(*C*H_3_)_3_ and NCOC(*C*H_3_)_3_], 46.6, 47.2 [CH_2_, N*C*H_2_], 50.3, 51.4 [CH_2_, benzyl *C*H_2_], 79.8 [C, *C*(CH_3_)_3_], 83.4 [C, *C*(CH_3_)_3_], 115.3 [CH, aromatic *C*H], 118.0 [C, aromatic *C*], 118.9 [CH, aromatic *C*H], 122.4 [CH, aromatic *C*H], 123.1 [CH, aromatic *C*H], 124.3 [CH, aromatic *C*H], 127.2 [CH, aromatic *C*H], 127.9 [CH, aromatic *C*H], 128.5 [CH, aromatic *C*H], 130.6 [C, aromatic *C*], 135.5 [C, aromatic *C*], 138.5 [C, aromatic *C*], 149.73 [C, *C*=O], 155.57, 155.97 [C, *C*=O]. HRMS (ESI+): Exact mass calculated for C_27_H_34_N_2_O_4_Na [M + Na]^+^ 473.2411. Found 473.2408.

### *tert*-Butyl 3-(2-((*tert*-butoxycarbonyl)amino)ethyl)-5-chloro-2-methyl-1*H*-indole-1-carboxylate (**9d**)


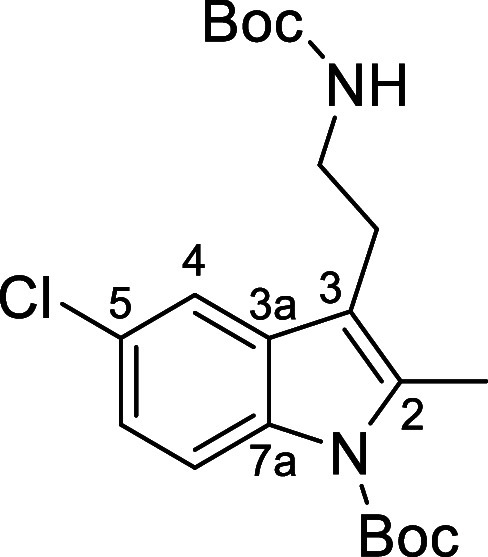
The title compound was prepared according to the *General Procedure A* using 2-(5-chloro-2-methyl-1*H*-indol-3-yl)ethan-1-amine (0.93 g, 4.50 mmol, 1.0 equiv), di-*tert*-butyl-dicarbonate (2 × 0.98 g, 9.0 mmol, 2.2 equiv), triethylamine (0.46 g, 4.50 mmol, 1.0 equiv), and DMAP (0.06 g, 0.45 mmol, 0.1 equiv) in acetonitrile (50 mL) at 0 °C. The crude product was purified by flash column chromatography using hexane/ethyl acetate (75:15) as eluent, which afforded the bis-Boc diamine **9d** as a yellow oil (1.28 g, 70%): ν_max_/cm^–1^ (ATR) 1698 (C=O), 1122 (C–O). δ_H_ (300 MHz, CDCl_3_): 1.44 [9H, s, NHCOC(C*H*_3_)_3_], 1.67 [9H, s, NCOC(C*H*_3_)_3_], 2.53 [3H, s, C*H*_3_], 2.82 [2H, t, *J* 6.7 Hz, C*H*_2_], 3.21–3.34 [2H, m, NHC*H*_2_], 4.59 [1H, br s, N*H*], 7.09–7.19 [1H, m, aromatic C*H*], 7.31–7.41 [1H, m, aromatic C*H*], 8.01 [1H, d, *J* 8.9 Hz, aromatic C*H*]. δ_C_ (75 MHz, CDCl_3_): 14.0 [CH_3_, *C*H_3_], 24.5 [CH_2_, *C*H_2_], 28.5 [NHCOC(*C*H_3_)_3_ and NCOC(*C*H_3_)_3_], 40.9 [CH_2_, *C*H_2_], 79.3 [C, *C*(CH_3_)_3_], 84.1 [C, *C*(CH_3_)_3_], 114.9 [C, aromatic *C*], 116.5 [CH, aromatic *C*H], 117.4 [CH, aromatic *C*H], 123.6 [CH, aromatic *C*H], 128.2 [C, aromatic *C*], 131.2 [C, aromatic *C*], 134.2 [C, aromatic *C*], 135.7 [C, aromatic *C*], 150.4 [C, *C*=O], 155.9 [C, *C*=O]. HRMS (ESI+): Exact mass calculated for C_21_H_29_ClN_2_O_4_Na [M + Na]^+^ 431.1708. Found 431.1702.

### Di-*tert*-butyl 8-chloro-3,4,9,9a-tetrahydro-1*H*-pyrido[4,3-*b*]indole-2,5-dicarboxylate (**9e**)


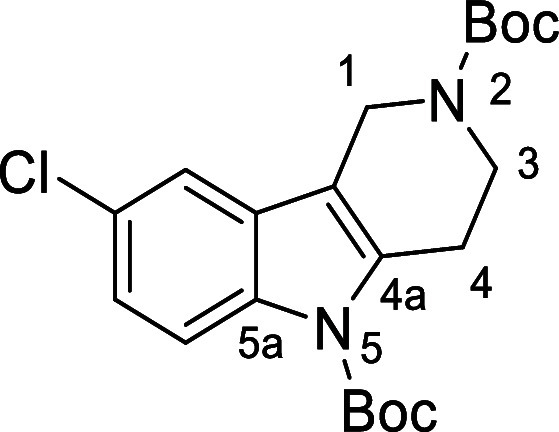
The title compound was prepared according to the *General Procedure A* using 8-chloro-2,3,4,5,9,9a-hexahydro-1*H*-pyrido[4,3-*b*]indole (0.80 g, 4.64 mmol, 1.0 equiv), di-*tert*-butyl-dicarbonate (2 × 1.27 g, 10.2 mmol, 2.2 equiv), triethylamine (0.46 g, 4.64 mmol, 1.0 equiv), and DMAP (0.06 g, 0.46 mmol, 0.1 equiv) in acetonitrile (50 mL) at 0 °C. The crude product was purified by flash column chromatography using hexane/ethyl acetate (75:15) as eluent, which afforded the bis-Boc diamine **9e** as an off-white crystalline solid (1.38 g, 80%): mp 187–191 °C. ν_max_/cm^–1^ (ATR) 1701 (C=O), 1130 (C–O). δ_H_ (300 MHz, CDCl_3_): 1.51 [9H, s, N(2)COC(C*H*_3_)_3_], 1.66 [9H, s, NCOC(C*H*_3_)_3_], 3.06–3.12 [2H, m, C(4)*H*_2_], 3.71–3.78 [2H, m, C(3)*H*_2_], 4.52 [2H, s, C(1)*H*_2_], 7.18–7.26 [1H, m, aromatic C*H*], 7.31–7.38 [1H, m, aromatic C*H*], 8.01–8.15 [1H, m, aromatic C*H*]. δ_C_ (75 MHz, CDCl_3_): 26.5 [CH_2_, *C*H_2_], 28.3, 28.5 [N(2)COC(*C*H_3_)_3_ and N(5)COC(*C*H_3_)_3_], 40.7 [CH_2_, 2 × *C*H_2_], 80.2 [C, *C*(CH_3_)_3_], 84.2 [C, *C*(CH_3_)_3_], 113.5 [C, aromatic *C*], 116.6 [CH, aromatic *C*H], 117.1 [CH, aromatic *C*H], 123.4 [CH, aromatic *C*H], 128.3 [C, aromatic *C*], 128.6 [C, aromatic *C*], 134.4 [C, aromatic *C*], 135.0 [C, aromatic *C*], 150.0 [C, *C*=O], 154.9 [C, *C*=O]. HRMS (ESI+): Exact mass calculated for C_21_H_27_N_2_O_4_ClNa [M + Na]^+^ 429.1552. Found 429.1551.

### Di-*tert-*butyl 3,4-dihydro-1*H*-pyrido[4,3-*b*]indole-2,5-dicarboxylate (**9f**)


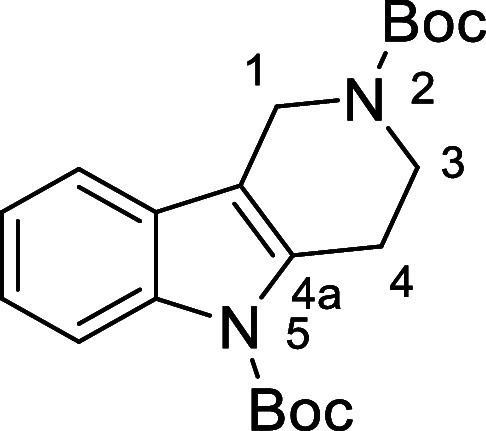
The title compound was prepared according to the *General Procedure A* using 2,3,4,5-tetrahydro-1*H*-pyrido[4,3-*b*]indole (0.80 g, 3.87 mmol, 1.0 equiv), di-*tert*-butyl-dicarbonate (2 × 0.93 g, 8.51 mmol, 2.2 equiv), triethylamine (0.39 g, 3.87 mmol, 1.0 equiv), and DMAP (0.05 g, 0.387 mmol, 0.1 equiv) in acetonitrile (50 mL) at 0 °C. The crude product was purified by flash column chromatography using hexane/ethyl acetate (75:15) as eluent, which afforded the bis-Boc diamine **9f** as a beige crystalline solid (1.05 g, 67%): mp 218–220 °C. ν_max_/cm^–1^ (ATR) 1696 (C=O), 1130 (C–O). δ_H_ (300 MHz, CDCl_3_): 1.51 [9H, s, N(2)COC(C*H*_3_)_3_], 1.66 [9H, s, N(5)COC(C*H*_3_)_3_], 3.09 [2H, t, *J* 6.9 Hz, C(4)*H*_2_], 3.69–3.79 [2H, m, C(3)*H*_2_], 4.56 [2H, s, C(1)*H*_2_], 7.17–7.26 [2H, m, 2 × aromatic C*H*], 7.27–7.39 [1H, m, aromatic C*H*], 8.15 [1H, d, *J* 7.8 Hz, aromatic C*H*]. δ_C_ (75 MHz, CDCl_3_): 26.4 [CH_2_, *C*(4)H_2_], 28.3 [CH_3_, N(2)COC(C*H*_3_)_3_], 28.5 [CH_3_, N(5)COC(C*H*_3_)_3_], 40.9 [br, CH_2_, × 2 *C*H_2_], 79.9 [C, *C*(CH_3_)_3_], 83.7 [C, *C*(CH_3_)_3_], 113.9 [C, aromatic *C*], 115.6 [CH, aromatic *C*H], 117.3 [CH, aromatic *C*H], 122.7 [CH, aromatic *C*H], 123.9 [CH, aromatic *C*H], 127.4 [C, aromatic *C*], 133.5 [C, aromatic *C*], 136.0 [C, aromatic *C*], 150.3 [C, *C*=O], 155.0 [C, *C*=O]. HRMS (ESI+): Exact mass calculated for C_21_H_28_N_2_O_4_Na [M + Na]^+^ 395.1941. Found 395.1936.

### *tert*-Butyl-5-((*tert*-butoxycarbonyl)amino)-3,4-dihydroisoquinoline-2(1*H*)-carboxylate (**9g**)


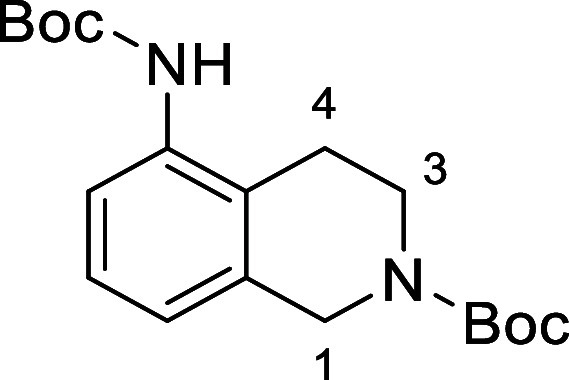
The title compound was prepared according to the *General ProcedureA* using di-*tert*-butyl-dicarbonate (0.75 g, 3.4 mmol, 2.2 equiv), 1,2,3,4-tetrahydroisoquinolin-5-amine (0.26 g, 1.7 mmol, 1.0 equiv), DMAP (0.04 g, 0.17 mmol, 0.1 equiv), and triethylamine (0.16 g, 1.7 mmol, 1.0 equiv) in acetonitrile (30 mL) at 0 °C to afford the pure bis-Boc compound **9g** as a colorless crystalline solid (0.39 g, 66%) without the need for purification: mp 211–214 °C. ν_max_/cm^–1^ (ATR) 1706 (C=O), 1115 (C–O). δ_H_ (300 MHz, CDCl_3_): 1.48 [9H, s, NCOC(C*H*_3_)_3_], 1.51 [9H, s, NHCOC(C*H*_3_)_3_], 2.67 [2H, t, *J* = 6.1 Hz, C(4)*H*_2_], 3.67 [2H, t, *J* = 5.9 Hz, C(3)*H*_2_], 4.56 [2H, s, C(1)*H*_2_], 6.23 [1H, bs, N*H*], 6.87 [1H, d, *J* 7.5 Hz, C*H*], 7.17 [1H, t, *J* 7.8 Hz, C*H*], 7.60 [1H, d, *J* 7.8 Hz, C*H*]. δ_c_ (75 MHz, CDCl_3_, broadened signals at 40.7 and 45.9 ppm) 24.2 [CH_2_, *C*H_2_], 28.3 [CH_3_, NHCOC(*C*H_3_)_3_], 28.5 [CH_3_, NCOC(*C*H_3_)_3_], 40.7 [CH_2_, *C*H_2_], 45.9 [CH_2_, *C*H_2_], 79.9 [C, *C*(CH_3_)_3_], 80.6 [C, *C*(CH_3_)_3_], 119.7 [CH, aromatic *C*H], 122.1 [CH, aromatic *C*H], 125.3 [C, aromatic *C*], 126.5 [CH, aromatic *C*H], 134.5 [C, aromatic *C*], 135.8 [C, aromatic *C*], 153.1 [C, *C*=O], 154.7 [C, *C*=O]. HRMS (ESI+): Exact mass calculated for C_19_H_28_N_2_O_4_Na [M + Na]^+^ 371.1941. Found 371.1944.

### Continuous Flow Procedure for the Deprotection of Protected Diamines **9a**–**j**

*General flow procedure B:* A solution of *N,N′-*di-Boc-amine **9a**–**j** (10 mL, 0.1 M, 1.0 equiv) in appropriate solvent was pumped through a stainless steel coil reactor (10 mL, 1 mm internal diameter) at temperatures ranging between 150–240 °C for a specified residence time. The reaction stream was passed through a series of three 8 bar back pressure regulators (24 bar) after which the reaction effluent was collected in a round-bottom flask and concentrated under reduced pressure to afford the crude deprotected mono-Boc diamines **10a**–**j** or fully deprotected amines **11a**–**j**. Chromatographic purification in some instances was undertaken as detailed below.

### *tert*-Butyl (2-(1*H*-indol-3-yl)ethyl)carbamate (**10a**)^46^


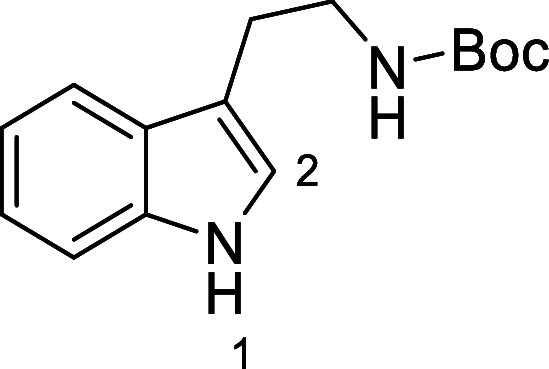
This compound was prepared according to the *General Flow Procedure B* using a temperature of 150 °C and residence time of 30 min from a methanol solution of *tert*-butyl 3-(2-((*tert*-butoxycarbonyl)amino)ethyl)-1*H*-indole-1-carboxylate (**9a**) (10 mL, 0.36 g, 0.1 M, 0.333 mL min^–1^). The crude product was purified by flash chromatography on silica gel with hexane/ethyl acetate (9:1) as eluent affording *N*-Boc amine **10a** as a white solid (0.23 g, 90%): mp 88–91 °C (lit.^46^ 90–92 °C). ν_max_/cm^–1^ (ATR) 3302 (NH), 1694 (C=O). δ_H_ (300 MHz, CDCl_3_): 1.44 [9H, s, NHCOC(C*H*_3_)_3_], 2.93 [2H, t, *J* 6.8 Hz, C*H*_2_], 3.35–3.49 [2H, m, C*H*_2_], 4.67 [1H, br s, N*H*], 6.94 [1H, s, aromatic C*H*], 7.05–7.23 [2H, m, aromatic C*H*], 7.29–7.35 [1H, m, aromatic C*H*], 7.58 [1H, d, *J* 7.8 Hz, aromatic C*H*], 8.34 [1H, br s, N*H*]. δ_C_ (75 MHz, CDCl_3_): 25.6 [CH_2_, *C*H_2_], 28.5 [CH_3_, C(*C*H_3_)_3_], 40.1 [CH_2_, C*H*_2_], 79.2 [C, *C*(CH_3_)_3_], 111.3 [CH, aromatic *C*H], 112.9 [C, aromatic *C*], 118.8 [CH, aromatic *C*H], 119.3 [CH, aromatic *C*H], 122.0 [CH, aromatic *C*H], 122.2 [CH, aromatic *C*H], 127.4 [C, aromatic *C*], 136.4 [C, aromatic *C*], 156.1 [C, *C*=O]. Spectroscopic characteristics were consistent with those reported in previous literature.^46^

### *tert*-Butyl (2-(2-methyl-1*H*-indol-3-yl)ethyl)carbamate (**10b**)^47^


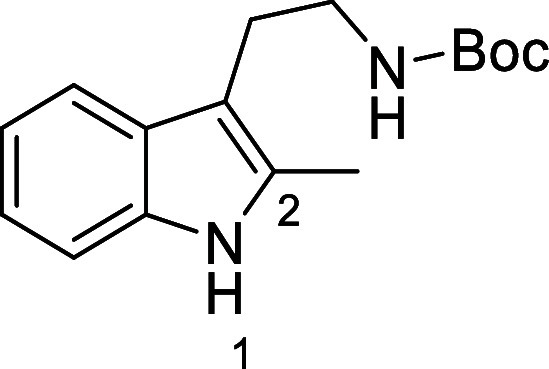
This compound was prepared according to the *General Flow Procedure B* using a temperature of 150 °C and residence time of 30 min from a methanol solution of *tert*-butyl 3-(2-((*tert*-butoxycarbonyl)amino)ethyl)-2-methyl-1*H*-indole-1-carboxylate (**9b**) (10 mL, 0.37 g, 0.1 M, 0.333 mL min^–1^). The crude product was purified by flash chromatography on silica gel with hexane/ethyl acetate (9:1) as eluent affording *N*-Boc amine **10b** as a yellow oil (0.24 g, 88%): ν_max_/cm^–1^ (ATR) 3310 (NH), 1684 (C=O). δ_H_ (300 MHz, CDCl_3_): 1.43 [9H, s, NHCOC(C*H*_3_)_3_], 2.26 [3H, s, C*H*_3_], 2.79–2.90 [2H, m, C*H*_2_], 3.21–3.33 [2H, m, C*H*_2_], 4.64 [1H, bs, N*H*], 7.01–7.10 [2H, m, 2 × aromatic C*H*], 7.13–7.21 [1H, m, aromatic C*H*], 7.40–7.49 [1H, m, aromatic C*H*], 8.40 [1H, br s, aromatic N*H*]; δ_c_ (75 MHz, CDCl_3_): 11.5 [CH_3_, *C*H_3_], 24.7 [CH_2_*C*H_2_], 28.6 [CH_3_, C(*C*H_3_)_3_], 41.2 [CH_2_, *C*H_2_], 79.2 [C, *C*(CH_3_)_3_], 108.3 [C, aromatic *C*], 110.5 [CH, aromatic *C*H], 117.9 [CH, aromatic *C*H], 119.2 [CH, aromatic *C*H], 120.9 [CH, aromatic *C*H], 128.6 [C, aromatic *C*], 132.3 [C, aromatic *C*], 135.5 [C, aromatic *C*], 156.2 [C, *C*=O]. Spectroscopic characteristics were consistent with those reported in previous literature.^47^

### *tert*-Butyl (2-(1*H*-indol-3-yl)ethyl)(benzyl)carbamate (**10c**)^48^


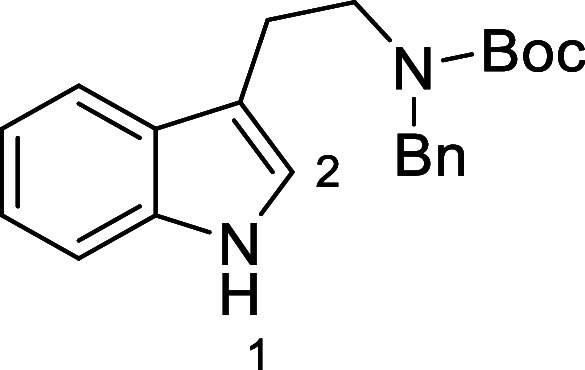
This compound was prepared according to the *General Flow Procedure B* using a temperature of 150 °C and residence time of 30 min from a methanol solution of *tert*-butyl 3-(2-(benzyl(*tert*-butoxycarbonyl)amino)ethyl)-1*H*-indole-1-carboxylate (**9c**) (10 mL, 0.45 g, 0.1 M, 0.333 mL min^–1^). The crude product was purified by flash chromatography on silica gel with hexane/ethyl acetate (9:1) as eluent affording *N*-Boc amine **10c** as a yellow oil (0.29 g, 82%): ν_max_/cm^–1^ (ATR) 3310 (NH), 1691 (C=O). δ_H_ (300 MHz, CDCl_3_): 1.45 [9H, s, NCOC(C*H*_3_)_3_], 2.91–3.01 [2H, m, C*H*_2_], 3.44–3.56 [2H, m, C*H*_2_], 4.38–4.48 [2H, m, C*H*_2_], 6.84–6.95 [1H, m, aromatic C*H*], 7.03–7.25 [8H, m, 8 × aromatic C*H*], 7.54 [1H, d, *J* 7.8 Hz, aromatic C*H*], 8.02 [1H, br s, aromatic N*H*]. δ_c_ (75 MHz, CDCl_3_): 24.2 [CH_2_, *C*H_2_], 28.5 [CH_3_, C(*C*H_3_)_3_], 47.6 [CH_2_, *C*H_2_], 50.8 [CH_2_, *C*H_2_], 79.6 [C, *C*(CH_3_)_3_], 111.2 [CH, aromatic *C*H], 113.5 [C, aromatic *C*], 118.8 [CH, aromatic *C*H], 119.3 [CH, aromatic *C*H], 121.8 [CH, aromatic *C*H], 121.9 [CH, aromatic *C*H], 127.1 [CH, aromatic *C*H], 127.5 [C, aromatic *C*], 127.8 [CH, aromatic *C*H], 128.4 [CH, aromatic *C*H], 136.4 [C, aromatic *C*], 138.6 [C, aromatic *C*], 155.9 [C, *C*=O]. Spectroscopic characteristics were consistent with those reported in previous literature.^48^

### *tert*-Butyl (2-(5-chloro-2-methyl-1*H*-indol-3-yl)ethyl)carbamate (**10d**)^48^


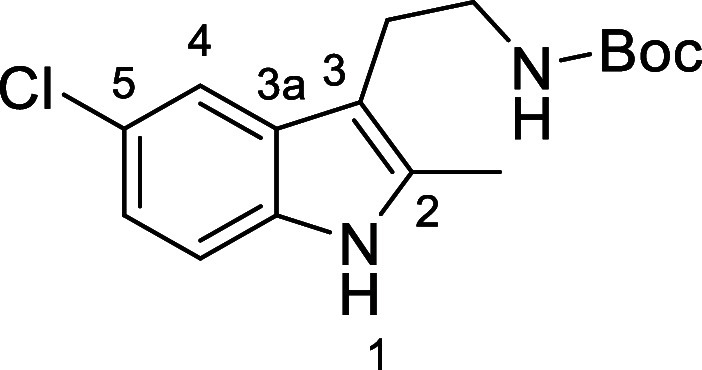
This compound was prepared according to the *General Flow Procedure B* using a temperature of 150 °C and residence time of 30 min from a methanol solution of *tert*-butyl 3-(2-((*tert*-butoxycarbonyl)amino)ethyl)-5-chloro-2-methyl-1*H*-indole-1-carboxylate (**9d**) (10 mL, 0.41 g, 0.1 M, 0.333 mL min^–1^). The crude product was purified by flash chromatography on silica gel with hexane/ethyl acetate (9:1) as eluent affording *N*-Boc amine **10d** as a yellow oil (0.26 g, 83%): ν_max_/cm^–1^ (ATR) 3315 (NH), 1698 (C=O). δ_H_ (300 MHz, CDCl_3_): 1.44 [9H, s, NHCOC(C*H*_3_)_3_], 2.34 [3H, s, C*H*_3_], 2.82 [2H, t, *J* 6.8 Hz, C*H*_2_], 3.21–3.35 [2H, m, C*H*_2_], 4.51 [1H, br s, NH], 6.98–7.21 [2H, m, 2 × aromatic C*H*], 7.38–7.45 [1H, m, aromatic C*H*], 8.07 [1H, br s, N*H*]. δ_c_ (75 MHz, CDCl_3_): 11.7 [CH_3_, *C*H_3_], 23.7 [CH_2_, *C*H_2_], 28.4 [CH_3_, C(*C*H_3_)_3_], 41.1 [CH_2_, *C*H_2_], 79.2 [C, *C*(CH_3_)_3_], 106.6 [C, aromatic *C*], 111.2 [CH, aromatic *C*H], 117.3 [CH, aromatic *C*H], 121.2 [CH, aromatic *C*H], 124.9 [C, aromatic *C*], 129.6 [C, aromatic *C*], 130.1 [C, aromatic *C*], 133.6 [C, aromatic *C*], 156.1 [C, *C*=O]. Spectroscopic characteristics were consistent with those reported in previous literature.^48^

### *tert*-Butyl 8-chloro-1,3,4,5-tetrahydro-*2H*-pyrido[4,3-*b*]indole-2-carboxylate (**10e**)^49^


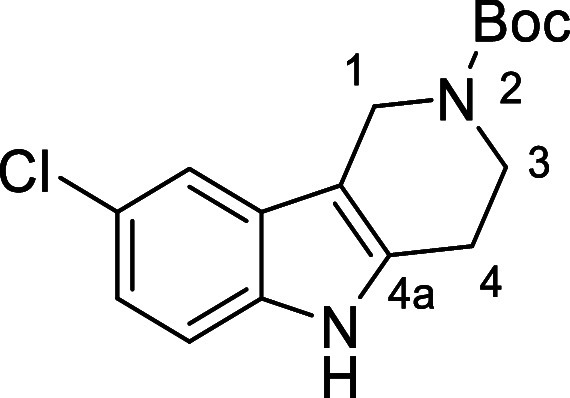
This compound was prepared according to the *General Flow Procedure B* using a temperature of 150 °C and residence time of 30 min from a methanol solution of di*-tert*-butyl 8-chloro-3,4-dihydro-1*H*-pyrido[4,3-*b*]indole-2,5-dicarboxylate (**9e**) (10 mL, 0.41 g, 0.1 M, 0.333 mL min^–1^). The crude product was purified by flash chromatography on silica gel with hexane/ethyl acetate (75:25) as eluent affording *N*-Boc amine **10e** as a yellow oil (0.24 g, 80%): ν_max_/cm^–1^ (ATR) 3324 (NH), 1651 (C=O). δ_H_ (300 MHz, CDCl_3_): 1.50 [9H, s, NCOC(C*H*_3_)_3_], 2.78–2.89 [2H, m, C(4)*H*_2_], 3.76–3.85 [2H, m, C(3)*H*_2_], 4.56 [2H, s, C(1)*H*_2_], 7.06–7.18 [1H, m, aromatic C*H*], 7.21–7.29 [1H, m, aromatic C*H*], 7.40 [1H, s, aromatic C*H*], 7.94 [1H, br s, N*H*]. δ_c_ (75 MHz, CDCl_3_): 23.6 [CH_2_, *C*H_2_], 28.5 [CH_3_, C(*C*H_3_)_3_], 40.6 [CH_2_, *C*H_2_], 41.7 [CH_2_, *C*H_2_], 80.1 [C, *C*(CH_3_)_3_], 107.8 [C, aromatic *C*], 111.6 [CH, aromatic *CH*], 117.2 [CH, aromatic *C*H], 121.7 [CH, aromatic *C*H], 126.2 [C, aromatic *C*], 133.6 [C, aromatic *C*], 134.2 [C, aromatic *C*], 135.7 [C, aromatic *C*], 150.5 [C, *C*=O]. Spectroscopic characteristics were consistent with those reported in previous literature.^49^

### *tert*-Butyl 1,3,4,5-tetrahydro-2*H*-pyrido[4,3-*b*]indole-2-carboxylate (**10f**)^49^


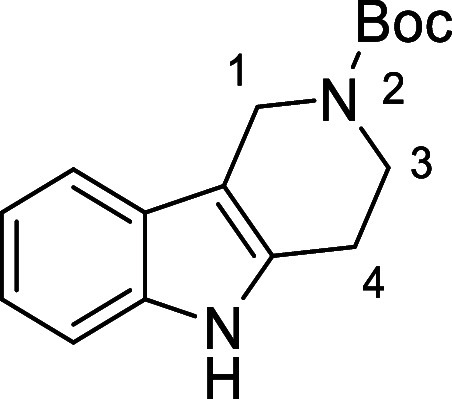
This compound was prepared according to the *General Flow Procedure B* using a temperature of 150 °C and residence time of 30 min from a methanol solution of di-*tert-*butyl 3,4-dihydro-1*H*-pyrido[4,3-*b*]indole-2,5-dicarboxylate (**9f**) (10 mL, 0.37 g, 0.1 M, 0.333 mL min^–1^). The crude product was purified by flash chromatography on silica gel with hexane/ethyl acetate (75:25) as eluent affording *N*-Boc amine **10f** as a yellow oil (0.31 g, 86%): ν_max_/cm^–1^ (ATR) 3334 (NH), 1651 (C=O). δ_H_ (300 MHz, CDCl_3_): 1.51 [9H, s, NCOC(C*H*_3_)_3_], 2.75–2.85 [2H, m, C(4)*H*_2_], 3.76–3.85 [2H, m, C(3)*H*_2_], 4.64 [2H, s, C(1)*H*_2_], 7.03–7.24 [2H, m, 2 × aromatic C*H*], 7.27–7.34 [1H, m, aromatic C*H*], 7.37–7.43 [1H, m, aromatic C*H*], 8.01 [1H, br s, N*H*]. δ_c_ (75 MHz, CDCl_3_): 23.6 [CH_2_, *C*H_2_], 28.3 [CH_3_, C(*C*H_3_)_3_], 40.7 [CH_2_, *C*H_2_], 41.4 [CH_2_, *C*H_2_], 79.9 [C, *C*(CH_3_)_3_], 107.6 [C, aromatic *C*], 110.8 [CH, aromatic *C*H], 117.6 [CH, aromatic *C*H], 119.6 [CH, aromatic *C*H], 121.6 [CH, aromatic *C*H], 125.6 [C, aromatic *C*], 132.2 [C, aromatic *C*], 135.9 [C, aromatic *C*], 155.3 [C, *C*=O]. Spectroscopic characteristics were consistent with those reported in previous literature.^49^

### *tert*-Butyl 5-amino-3,4-dihydroisoquinoline-2(1*H*)-carboxylate (**10g**)^50^


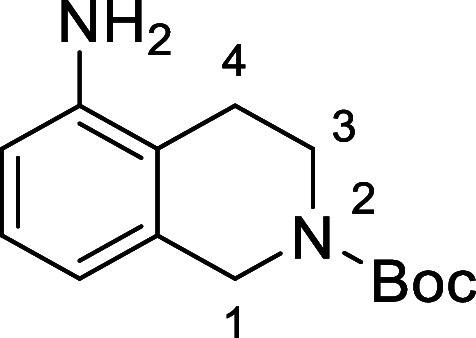
This compound was prepared according to the *General Flow Procedure B* using a temperature of 170 °C and residence time of 90 min from a methanol solution of *tert*-butyl 5-((*tert*-butoxycarbonyl)amino)-3,4-dihydroisoquinoline-2(1*H*)-carboxylate (**9g**) (0.34 g, 0.1 M, 1 mL min^–1^). The crude product was purified by flash chromatography on silica gel with hexane/ethyl acetate (75:25) as eluent affording *N*-Boc amine **10g** as a yellow oil (0.10 g, 43%): ν_max_/cm^–1^ (ATR) 3310 (NH), 1651 (C=O). δ_H_ (300 MHz, CDCl_3_): 1.48 [9H, s, NCOC(C*H*_3_)_3_], 2.56 [2H, t, *J* 6.9 Hz, C*H*_2_], 3.70 [2H, t, *J* 6.0 Hz, C*H*_2_], 4.54 [2H, s, C*H*_2_], 6.49–6.61 [2H, m, 2 × aromatic *C*H], 6.95–7.09 [1H, m, aromatic C*H*]. δ_c_ (75 MHz, CDCl_3_, evidence of rotamers at signals between 40 and 50 ppm): 23.7 [CH_2_, *C*H_2_], 28.5 [CH_3_, C(*C*H_3_)_3_], 40.12, 41.4 [CH_2_, *C*H_2_], 45.3, 46.1 [CH_2_, *C*H_2_], 79.8 [C, *C*(CH_3_)_3_], 113.2 [CH, aromatic *C*H], 117.0 [CH, aromatic *C*H], 120.1 [C, aromatic *C*], 126.7 [CH, aromatic *C*H], 134.6 [C, aromatic *C*], 143.5 [C, aromatic *C*], 154.8 [C, *C*=O]. Spectroscopic characteristics were consistent with those reported in previous literature.^50^

### *tert*-Butyl (4-aminophenethyl)carbamate (**10h**)^51^


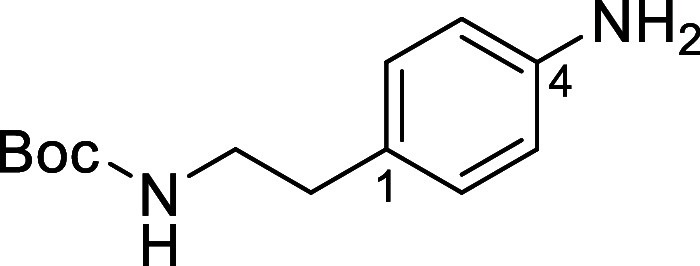
This compound was prepared according to the *General Flow Procedure B* using a temperature of 230 °C and residence time of 10 min from an acetonitrile/acetone (8:2) solution of *tert*-butyl (4-(2-((*tert*-butoxycarbonyl)amino)ethyl)phenyl)carbamate (**9h**) (0.17 g, 0.05 M, 1 mL min^–1^). The crude product was purified by flash chromatography on silica gel with hexane/ethyl acetate (8:2) a eluent affording *N*-Boc amine **10h** as light brown solid (0.083 g, 70%): mp 152–154 °C. ν_max_/cm^–1^ (ATR) 3334 (NH), 1708 (C=O). δ_H_ (500 MHz, CDCl_3_): 1.43 [9H, s, NHCOC(C*H*_3_)_3_], 2.67 [2H, t, *J* 6.8 Hz, C*H*_2_], 3.25–3.38 [2H, m, C*H*_2_], 4.60 [1H, br s, N*H*], 6.61–6.65 [2H, m, 2 × aromatic C*H*], 6.96 [2H, d, *J* 8.1 Hz, 2 × aromatic C*H*]. δ_c_ (125 MHz, CDCl_3_): 28.4 [CH_3_, NHCOC(*C*H_3_)_3_], 35.2 [CH_2_, *C*H_2_], 42.0 [CH_2_, *C*H_2_], 79.1 [C, *C*(CH_3_)_3_], 115.4 [CH, 2 × aromatic *C*H], 128.9 [C, aromatic *C*], 129.6 [CH, 2 × aromatic *C*H], 144.7 [C, aromatic *C*], 155.6 [C, *C*=O]. Spectroscopic characteristics were consistent with those reported in previous literature.^51^

### *tert*-Butyl (2-(piperazin-1-yl)ethyl)carbamate (**10i**)^52^


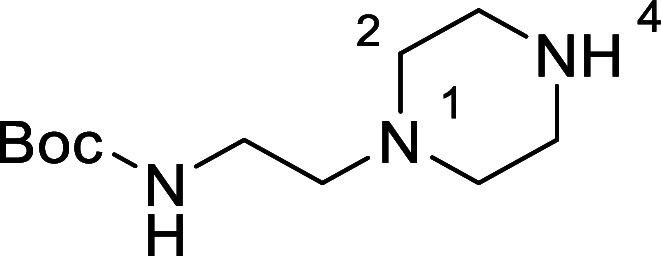
This compound was prepared according to the *General Flow Procedure B* using a temperature of 150 °C and residence time of 45 min from a methanol solution of *tert*-butyl 4-(2-((*tert*-butoxycarbonyl)amino)ethyl)piperazine-1-carboxylate (**9i**) (0.33 g, 0.1 M, 1 mL min^–1^). The crude product was purified by flash chromatography on silica gel with hexane/ethyl acetate (75:25) as eluent affording *N*-Boc amine **10i** as a yellow oil (0.03 g, 10%): ν_max_/cm^–1^ (ATR) 3310 (NH), 1710 (C=O). δ_H_ (400 MHz, CDCl_3_): 1.45 [9H, s, NHCOC(C*H*_3_)_3_], 2.19–2.29 [2H, m, C*H*_2_], 2.32–2.47 [4H, m, C*H*_2_], 2.85–2.96 [4H, m, C*H*_2_], 3.12–3.28 [2H, m, C*H*_2_], 5.09 [1H, br s, N*H*]. δ_c_ (100 MHz, CDCl_3_): 28.4 [CH_3_, NHCOC(*C*H_3_)_3_], 36.9 [CH_2_, *C*H_2_], 45.7 [CH_2_, 2 × *C*H_2_], 53.9 [CH_2_, 2 × *C*H_2_], 57.7 [CH_2_, *C*H_2_], 79.0 [C, *C*(CH_3_)_3_], 155.9 [C, *C*=O]. Spectroscopic characteristics for the above compound were consistent with those reported in previous literature.^52^

### *tert*-Butyl piperidin-4-ylcarbamate (**10j**)^53^


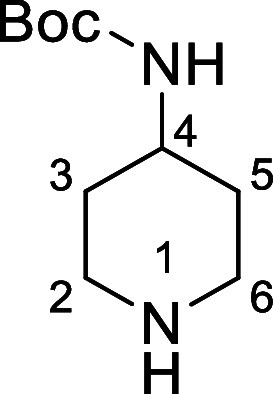
This compound was prepared according to the *General Flow Procedure B* using a temperature of 130 °C and residence time of 80 min from a methanol solution of *tert*-butyl 4-((*tert*-butoxycarbonyl)amino)piperidine-1-carboxylate (**9j**) (0.30 g, 0.1 M, 1 mL min^–1^). The crude product was purified by flash chromatography on silica gel with hexane/ethyl acetate (75:25) as eluent affording *N*-Boc amine **10j** (0.024 g, 12%): ν_max_/cm^–1^ (ATR) 3310 (NH), 1651 (C=O), 1431 (C–N). δ_H_ (400 MHz, CDCl_3_): 1.21–1.31 [2H, m, C*H*_2_], 1.45 [9H, s, NHCOC(C*H*_3_)_3_], 1.85–1.95 [2H, m, C*H*_2_], 2.78–2.89 [2H, m, C*H*_2_], 3.45–3.59 [1H, m, C*H*], 3.98–4.05 [2H, C*H*_2_], 4.46 [1H, br s, N*H*]. δ_c_ (100 MHz, CDCl_3_): 24.4 [CH_3_, NHCOC(*C*H_3_)_3_], 32.4 [CH_2_, 2 × *C*H_2_], 42.6 [CH_2_, 2 × *C*H_2_], 47.9 [CH, *C*H], 79.5 [C, *C*(CH_3_)_3_], 155.1 [C, *C*=O]. Spectroscopic characteristics for the above compound were consistent with those reported in previous literature.^53^

### Tryptamine (**11a**)^54^


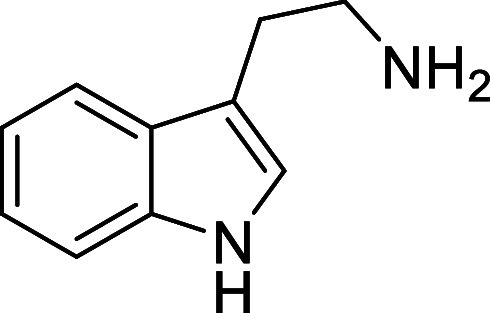
This compound was prepared according to the *General Flow Procedure B* using a temperature of 225 °C and residence time of 45 min from a methanol solution of *tert*-butyl 3-(2-((*tert*-butoxycarbonyl)amino)ethyl)-1*H*-indole-1-carboxylate (**9a**) (10 mL, 0.36 g, 0.1 M, 0.222 mL min^–1^). The crude product was purified by flash chromatography on silica gel with DCM/MeOH (95:5) as eluent affording deprotected tryptamine (**11a**) as white solid (0.14 g, 90%): mp 112–115 °C (lit.^54^ 112–114 °C). ν_max_/cm^–1^ (ATR) 3498 (NH_2_), 1345 (C–N stretch). δ_H_ (400 MHz, CDCl_3_): 1.29 [2H, br s, N*H*_*2*_], 2.89–2.96 [2H, m, C*H*_2_], 2.98–3.10 [2H, m, C*H*_2_], 7.01 [1H, s, aromatic C*H*], 7.09–7.25 [2H, m, aromatic C*H*], 7.31–7.48 [1H, m, aromatic C*H*], 7.58–7.69 [1H, m, aromatic C*H*], 8.40 [1H, br s, N*H*]. δ_c_ (100 MHz, CDCl_3_): 29.5 [CH_2_, *C*H_2_], 42.4 [CH_2_, *C*H_2_], 111.2 [CH, aromatic *C*H], 113.7 [C, aromatic *C*], 118.8 [CH, aromatic *C*H], 119.2 [CH, aromatic *C*H], 121.9 [CH, aromatic *C*H], 122.1 [CH, aromatic *C*H], 127.5 [C, aromatic *C*], 136.5 [C, aromatic *C*]. Spectroscopic characteristics were consistent with those reported in previous literature.^54^

### 2-(2-Methyl-1*H*-indol-3-yl)ethan-1-amine (**11b**)^54^


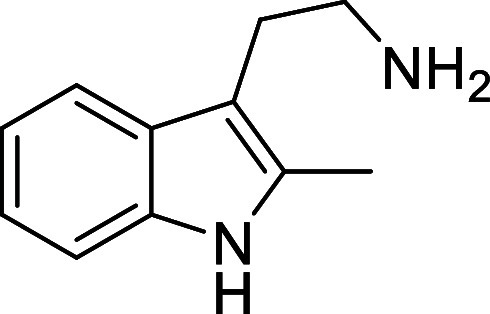
This compound was prepared according to the *General Flow Procedure B* using a temperature of 230 °C and residence time of 45 min from a methanol solution of *tert*-butyl 3-(2-((*tert*-butoxycarbonyl)amino)ethyl)-2-methyl-1*H*-indole-1-carboxylate (**9b**) (10 mL, 0.37 g, 0.1 M, 0.167 mL min^–1^). The crude product was purified by flash chromatography on silica gel with DCM/MeOH (95:5) as eluent affording deprotected diamine **11b** as a dark yellow oil (0.14 g, 83%, ∼95% pure): ν_max_/cm^–1^ (ATR): 3495 (NH_2_), 1350 (C–N). δ_H_ (400 MHz, CDCl_3_): 1.67 [2H, br s, N*H*_2_], 2.32 [3H, s, C*H*_3_], 2.79–2.87 [2H, m, C*H*_2_], 2.90–3.02 [2H, m, C*H*_2_], 7.01–7.11 [2H, m, aromatic C*H*], 7.19–7.25 [1H, m, aromatic C*H*], 7.45–7.52 [1H, m, aromatic C*H*], 8.28 [1H, br s, N*H*]. δ_c_ (100 MHz, CDCl_3_): 11.7 [CH_3_, *C*H_3_], 28.1 [CH_2_, *C*H_2_], 42.6 [CH_2_, *C*H_2_], 108.6 [C, aromatic *C*], 110.3 [CH, aromatic *C*H], 117.9 [CH, aromatic *C*H], 119.1 [CH, aromatic *C*H], 120.9 [CH, aromatic *C*H], 128.8 [C, aromatic *C*], 132.0 [C, aromatic *C*], 135.4 [C, aromatic *C*]. Spectroscopic characteristics were consistent with those reported in previous literature.^54^

### *N*-Benzyl-2-(1*H*-indol-3-yl)ethan-1-amine (**11c**)^54^


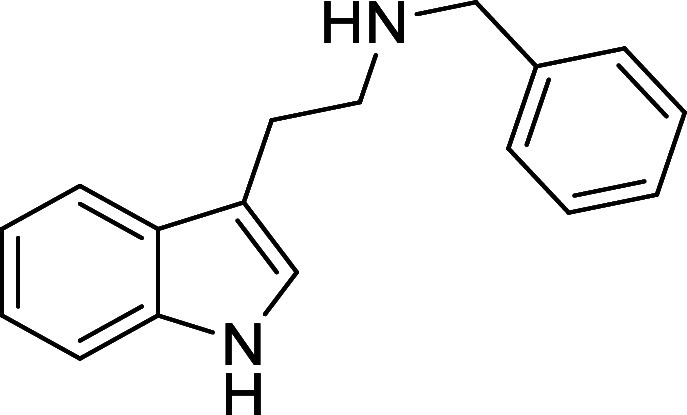
This compound was prepared according to the *General Flow Procedure B* using a temperature of 230 °C and residence time of 45 min from a methanol solution of *tert*-butyl-3-(2-(benzyl(*tert*-butoxycarbonyl)amino)ethyl)-1*H*-indole-1-carboxylate (**9c**) (10 mL, 0.45 g, 0.1 M, 0.333 mL min^–1^, 40 min residence time). The crude product was purified by flash chromatography on silica gel with hexane/ethyl acetate (80:20) as eluent affording deprotected diamine **11c** as a pale yellow oil (0.23 g, 73%): υ_max_/cm^–1^ (ATR): 3379 (NH), 1251 (C–N). δ_H_ (400 MHz, CDCl_3_): 1.94 [br s, N*H*], 2.98 [4H, s, 2 × C*H*_2_], 3.79 [2H, s, C*H*_2_], 6.93 [1H s, aromatic C*H*], 7.05–7.44 [9H, m, aromatic C*H*], 7.58 [1H, d, *J* 7.9 Hz, aromatic C*H*], 8.19 [1H, s, N*H*]. δ_C_ (100 MHz, CDCl_3_, evidence of rotamers at signals 122.0, 126.9, 127.5): 25.8 [*C*H_2_], 49.7 [*C*H_2_], 53.9 [*C*H_2_], 111.2 [CH, aromatic *C*H], 113.9 [C, aromatic *C*], 118.9 [CH, aromatic *C*H], 119.3 [CH, aromatic *C*H], 122.0 [CH, aromatic *C*H], 126.9 [CH, aromatic *C*H], 127.5 [C, aromatic *C*], 128.2 [CH, aromatic *C*H), 128.4 [CH, aromatic *C*H], 128.6 [CH, aromatic *C*H], 136.5 [C, aromatic *C*], 140.2 [C, aromatic *C*]. This compound was used without any further purification. Spectroscopic characteristics were consistent with those reported in previous literature.^54^

### 2-(5-Chloro-2-methyl-1*H*-indol-3-yl)ethan-1-amine (**11d**)^54^


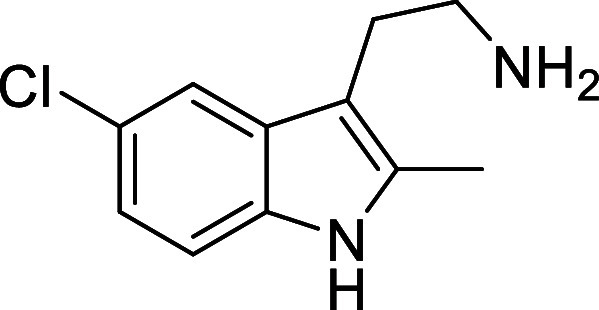
This compound was prepared according to the *General Flow Procedure B* using a temperature of 230 °C and residence time of 45 min from a methanol solution of *tert*-butyl 3-(2-((*tert*-butoxycarbonyl)amino)ethyl)-5-chloro-2-methyl-1*H*-indole-1-carboxylate (**9d**) (10 mL, 0.41 g, 0.1 M, 0.167 mL min^–1^). The crude product was purified by flash chromatography on silica gel with DCM/MeOH (95:5) as eluent affording deprotected diamine **11d** as a dark yellow oil (0.17 g, 82%,): ν_max_/cm^–1^ (ATR): 3491 (NH_2_), 1349 (C–N). δ_H_ (300 MHz, methanol-*d*_4_): 1.10 [3H, s, C*H*_3_], 1.68–1.83 [4H, m, 2 × C*H*_2_], 5.61–5.71 [1H, m, aromatic C*H*], 5.88–5.95 [1H, m, aromatic C*H*], 6.17 [1H, s, aromatic C*H*]. Spectroscopic characteristics were consistent with those reported in previous literature.^54^

### 8-Chloro-2,3,4,5-tetrahydro-1*H*-pyrido[4,3-*b*]indole (**11e**)^49^


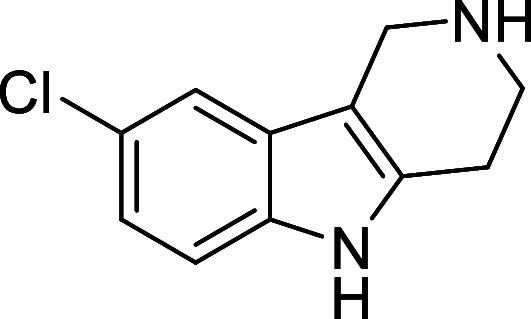
This compound was prepared according to the *General Flow Procedure B* using a temperature of 180 °C and residence time of 45 min from a trifluoroethanol solution of di-*tert*-butyl 8-chloro-3,4-dihydro-1*H*-pyrido[4,3-*b*]indole-2,5-dicarboxylate (**9e**) (10 mL, 0.41 g, 0.1 M, 0.222 mL min^–1^). The crude product was purified by flash chromatography on silica gel with DCM/MeOH (95:5) as eluent affording deprotected diamine **11e** as a beige solid (0.09 g, 55%): mp 241–243 °C (lit.^48^ 240–243 °C). ν_max_/cm^–1^ (ATR): 3309 (NH), 1320 (C–N). δ_H_ (400 MHz, DMSO-*d*_6_): 2.67–2.73 [2H, m, C*H*_2_], 2.99–3.10 [2H, m C*H*_2_], 3.86 [2H, s, C*H*_2_], 6.95–7.03 [1H, m, aromatic C*H*], 7.23–7.31 [1H, m, aromatic C*H*], 7.32–7.38 [1H, m, aromatic C*H*], 10.99 [1H, br s, N*H*]. δ_c_ (100 MHz, DMSO-*d*_6_): 24.5 [CH_2_, *C*H_2_], 42.6 [CH_2_, *C*H_2_], 43.3 [CH_2_, *C*H_2_], 107.6 [C, aromatic *C*], 112.1 [CH, aromatic *C*H], 116.4 [CH, aromatic *C*H], 119.9 [CH, aromatic *C*H], 122.8 [C, aromatic *C*], 126.7 [C, aromatic *C*], 133.9 [C, aromatic *C*], 135.1 [C, aromatic *C*]. Spectroscopic characteristics were consistent with those reported in previous literature.^49^

### 2,3,4,5-Tetrahydro-1*H*-pyrido[4,3-*b*]indole (**11f**)^49^


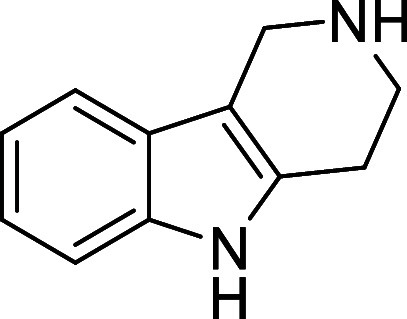
This compound was prepared according to the *General Flow Procedure B* using a temperature of 180 °C and residence time of 50 min from a trifluoroethanol solution of di-*tert*-butyl 3,4-dihydro-1*H*-pyrido[4,3-*b*]indole-2,5-dicarboxylate (**9f**) (10 mL, 0.37 g, 0.1 M, 0.250 mL min^–1^). The crude product was purified by flash chromatography on silica gel with hexane/ethyl acetate (75:25) as eluent affording deprotected diamine **11f** as a beige solid (0.15 g, 70%): mp 214–216 °C. ν_max_/cm^–1^ (ATR): 3401 (NH), 3260 (NH). δ_H_ (600 MHz, DMSO-*d*_6_): 2.63–2.69 [2H, m, C*H*_2_], 2.98–3.04 [2H, m, C*H*_2_ ], 3.84 [2H, s, C*H*_2_], 6.86–7.05 [2H, m, aromatic C*H*], 7.21–7.33 [2H, m, aromatic C*H*], 10.71 [1H, s, N*H*]. δ_c_ (150 MHz, DMSO-*d*_6_): 24.6 [CH_2_, *C*H_2_], 42.2 [CH_2_, *C*H_2_], 43.5 [CH_2_, *C*H_2_], 108.7 [C, aromatic *C*], 111.1 [CH, aromatic CH], 117.5 [CH, aromatic *C*H], 118.5 [CH, aromatic *C*H], 120.5 [CH, aromatic *CH*], 126.1 [C, aromatic *C*], 133.8 [C, aromatic *C*], 135.8 [C, aromatic *C*]. Spectroscopic characteristics were consistent with those reported in previous literature.^49^

### 1,2,3,4-Tetrahydroisoquinolin-5-amine (**11g**)^56^


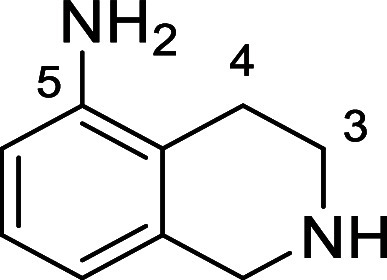
This compound was prepared according to the *General Flow Procedure B* using a temperature of 230 °C and residence time of 35 min from a methanol solution of *tert*-butyl 5-((*tert*-butoxycarbonyl)amino)-3,4-dihydroisoquinoline-2(1*H*)-carboxylate (**9g**) (10 mL, 0.35 g, 0.1 M, 0.250 mL min^–1^). The crude product was purified by flash chromatography on silica gel with DCM/MeOH (95:5) as eluent affording deprotected diamine **11g** as a yellow oil (0.12 g, 79%): ν_max_/cm^–1^ (ATR): 3360 (NH_2_). δ_H_ (400 MHz, CDCl_3_): 1.68 [1H, br s, N*H*], 2.40–2.49 [2H, m C*H*_2_], 3.15–3.24 [2H, m C*H*_2_], 3.57 [2H, br s, N*H*_2_], 3.96 [2H, s, C*H*_2_], 6.43–6.57 [2H, m, 2 × aromatic C*H*], 6.89–6.99 [1H, m, aromatic C*H*]. δ_c_ (100 MHz, CDCl_3_): 24.2 [CH_2_, *C*H_2_], 43.9 [CH_2_, *C*H_2_], 48.7 [CH_2_, *C*H_2_], 112.6 [CH, aromatic *C*H], 116.5 [CH, aromatic *C*H], 119.6 [C, aromatic *C*], 126.2 [CH, aromatic *C*H], 136.9 [C, aromatic *C*], 144.4 [C, aromatic *C*]. Spectroscopic characteristics were consistent with those reported in previous literature.^56^

### 4-(2′-Aminoethyl)aniline (**11h**)^55^


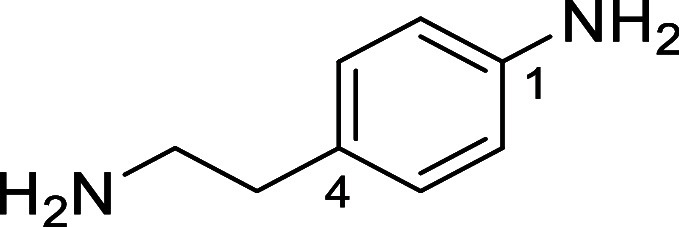
This compound was prepared according to the *General Flow Procedure B* using a temperature of 240 °C and residence time of 45 min from a methanol solution of *tert*-butyl (4-(2-((*tert*-butoxycarbonyl)amino)ethyl)phenyl)carbamate (**9h**) (10 mL, 0.34 g, 0.1 M, 0.200 mL min^–1^). The crude product was purified by flash chromatography on silica gel with DCM/MeOH (95:5) as eluent affording deprotected diamine **11h** as a yellow oil (0.12 g, 88%): ν_max_/cm^–1^ (ATR): 3379 (NH). δ_H_ (400 MHz, CDCl_3_): 2.45–2.53 [2H, m, C*H*_2_], 2.71–2.81 [2H, m, C*H*_2_], 6.46–6.52 [2H, 2 × aromatic C*H*], 6.83–6.91 [2H, 2 × aromatic C*H*]. δ_c_ (100 MHz, CDCl_3_): 39.3 [CH_2_, *C*H_2_], 43.8 [CH_2_, *C*H_2_], 115.3 [C*H*, 2 × aromatic C*H*], 129.6 [CH, 2 × aromatic *C*H], 144.7 [C, aromatic *C*]. Signal for one quaternary carbon overlapping with another signal. Spectroscopic characteristics were consistent with those reported in previous literature.^55^

### 2-(Piperidin-4-yl)ethan-1-amine (**11i**)^57^


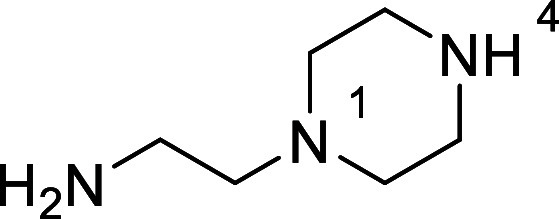
This compound was prepared according to the *General Flow Procedure B* using a temperature of 235 °C and residence time of 45 min from a methanol solution of *tert*-butyl 4-(2-((*tert*-butoxycarbonyl)amino)ethyl)piperazine-1-carboxylate (**9i**) (10 mL, 0.33 g, 0.1 M, 0.167 mL min^–1^), which after evaporation of the reaction solvent afforded deprotected diamine **11i** as a dark yellow oil (0.12 g, 90%): ν_max_/cm^–1^ (ATR): 3504 (NH_2_), 3349 (NH). δ_H_ (400 MHz, CDCl_3_): 1.41 [3H, br s, N*H*_2_ and N*H*], 2.31–2.42 [6H, m, 3 × C*H*_2_], 2.74–2.81 [2H, m, C*H*_2_], 2.83–2.91 [4H, m, 2 × C*H*_2_]; δ_c_ (100 MHz, CDCl_3_): 38.0 [CH_2_, *C*H_2_], 45.6 [CH_2_, 2 × *C*H_2_], 54.1 [CH_2_, 2 × *C*H_2_], 63.3 [CH_2_, *C*H_2_]. Spectroscopic characteristics were consistent with those reported in previous literature.^57^

### Piperidin-4-amine (**11j**)^58^


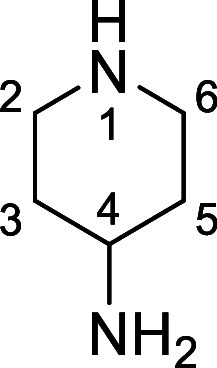
This compound was prepared according to the *General Flow Procedure B* using a temperature of 235 °C and residence time of 45 min from a methanol solution of *tert*-butyl 4-((*tert*-butoxycarbonyl)amino)piperidine-1-carboxylate (**9j**) (10 mL, 0.30 g, 0.1 M, 0.167 mL min ^–1^), which after evaporation of the reaction solvent afforded deprotected diamine **11j** as a dark yellow oil (0.08 g, 90%): ν_max_/cm^–1^ (ATR): 3505 (NH_2_), 3355 (NH). δ_H_ (400 MHz, CDCl_3_): 1.16–1.29 [2H, m], 1.65–2.80 {8H, m, containing 1.75–1.89 [2H, m], 2.07 [3H, br s, NH and NH_2_], 2.53–2.62 [2H, m], 2.65–2.78 [1H, m, C*H*]}, 2.95–3.12 [2H, m]. δ_c_ (100 MHz, CDCl_3_): 36.8 [CH_2_, 2 × *C*H_2_], 45.2 [CH_2_, 2 × *C*H_2_], 48.7 [CH, *C*H]. Spectroscopic characteristics for the above compound were consistent with those reported in previous literature.^58^

### Telescoped Synthesis of *tert*-Butyl (4-benzamidophenethyl)carbamate (**12**)


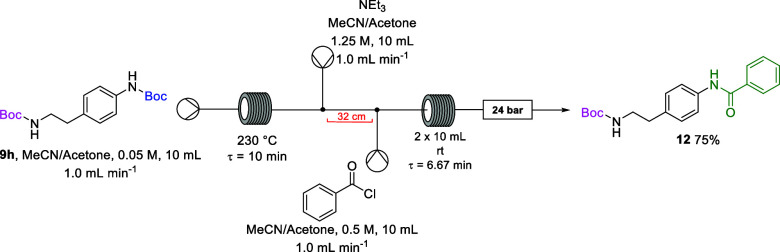
*tert*-Butyl-(4-(2-((*tert*-butoxycarbonyl)amino)ethyl)phenyl)carbamate (**9h**) was dissolved in acetonitrile/acetone (8:2) to make a 10 mL solution that was pumped into a stainless steel coil reactor heated to 230 °C for a residence time of 10 min (10 mL, 0.05 M, 1.0 equiv, 1.0 mL min^–1^). The emerging reaction stream was pumped through a micromixer T-piece where it met an acetonitrile/acetone (8:2) solution of triethylamine (10 mL, 1.25 M, 2.5 equiv, 1.0 mL min^–1^). The combined stream was pumped through a 32 cm piece of PFA tubing where it met a solution of benzoyl chloride in acetonitrile/acetone (10 mL, 0.05 M, 1.0 equiv, 1.0 mL min^–1^) at a T-piece. The combined streams were pumped through 2 × PFA reactor coils at 30 °C to give a residence time of 6.67 min after which the reactor effluent passed through a series of back-pressure regulators (3 × 8 bar) and was collected in a round-bottom flask. The reaction effluent was concentrated under reduced pressure, and the residue was dissolved in DCM (15 mL) and washed with water (20 mL), brine (20 mL), dried with MgSO_4_, and filtered. The solvent was removed under reduced pressure, and the crude product was subsequently purified by column chromatography on silica gel using hexane/ethyl acetate (8:2) as eluent affording amide **12** as an off-white solid (0.11 g, 75%): ν_max_/cm^–1^ (ATR): 3505 (NH_2_), 3355 (NH). δ_H_ (500 MHz, CDCl_3_): 1.43 [9H, s, NHCOC(C*H*_3_)_3_], 2.78 [2H, t, *J* 6.9 Hz, C*H*_2_], 3.30–3.39 [2H, m, C*H*_2_], 4.57 [1H, br s, N*H*], 7.19 [2H, d, *J* 8.9 Hz, 2 × aromatic C*H*], 7.46–7.60 [5H, m, aromatic C*H*], 7.83–7.90 [2H, m, 2 × aromatic C*H* ]; δ_c_ (125 MHz, CDCl_3_): 28.4 [CH_3_, C(*C*H_3_)_3_], 35.6 [CH_2_, *C*H_2_], 41.8 [CH_2_, *C*H_2_], 79.3 [C, *C*(CH_3_)_3_], 120.6 [CH, 2 × aromatic *C*H], 127.0 [CH, 2 × aromatic *C*H], 128.8 [CH, aromatic 2 × *C*H], 129.4 [CH, 2 × aromatic *C*H], 131.8 [CH, aromatic *C*H], 135.0 [C, aromatic *C*], 135.4 [C, aromatic *C*], 136.2 [C, aromatic *C*], 155.9 [*C*=O], 165.7 [*C*=O].

## Supporting Information Available

Further details of preparation and characterization of mono-Boc amine substrates (**8a**−**n**), bis-Boc diamine substrates (**9a**, **9b**, **9h**−**j**) and diamines (**11b**, **11c**, **11e**, and **11f**) including ^1^H and ^13^C NMR spectra (PDF)
