# Supplementary material for: Selective Thermal Deprotection of N-Boc Protected Amines in Continuous Flow
Source: Org Process Res Dev. 2024 Apr 25;28(5):1946–63. doi: 10.1021/acs.oprd.3c00498 (PMC11110071; doi:10.1021/acs.oprd.3c00498)
Supplement: Supplementary file 1 — op3c00498_si_001.pdf [file op3c00498_si_001.pdf]

Supporting Information

Selective Thermal Deprotection of *N*-Boc Protected Amines in Continuous Flow

Michelle-Rose Ryan,[¥] Denis Lynch,[¥] Stuart G. Collins,*[§] Anita R. Maguire*[¤]

[¥]School of Chemistry, Analytical and Biological Chemistry Research Facility, University College Cork, Cork T12 YN60, Ireland

[§]School of Chemistry, Analytical and Biological Chemistry Research Facility, SSPC, The SFI Research Centre for Pharmaceuticals, University College Cork, Cork T12 YN60, Ireland

[¤]School of Chemistry and School of Pharmacy, Analytical and Biological Chemistry Research Facility, SSPC, The SFI Research Centre for Pharmaceuticals, University College Cork, Cork T12 YN60, Ireland

*Email: a.maguire@ucc.ie *Email: stuart.collins@ucc.ie

Table of Contents

1. General Information S2

1.1 Materials and Methods S2

1.2 Instrumentation and Analysis S2

1.3 Continuous Flow Details and Specifications S3

2. Synthesis and Characterization of *N*-Boc Amines **8a-n** S4

3. Synthesis of Diamines **11b, 11c, 11e** and **11f** S12

4. Synthesis and Characterization of Bis-Boc Diamines **9a, 9b, 9h-j** S15

5. Spectroscopic Data of Novel Bis-Boc Diamines **9c-g** S18

6. Copies of ^{1}H NMR and ^{13}C NMR Spectra S23

1. General Information

1.1 Materials and Methods

Solvents were distilled prior to use as follows: dichloromethane was distilled from phosphorus pentoxide, ethyl acetate was distilled from potassium carbonate, tetrahydrofuran was distilled from sodium benzophenone ketyl in a nitrogen atmosphere, hexane was distilled prior to use. Methanol, toluene and HPLC grade acetonitrile were obtained from Sigma-Aldrich. Trifluoroethanol was obtained commercially from Fluorochem. All commercial reagents were used without further purification unless otherwise stated.

1.2 Instrumentation and Analysis

^{1}H (300 MHz) and ^{13}C (75.5 MHz) NMR spectra were recorded on a Bruker Avance 300 MHz NMR spectrometer. ^{1}H (400 MHz) and ^{13}C (100.6 MHz) NMR spectra were recorded on a Bruker Avance 400 MHz NMR spectrometer. ^{1}H (500 MHz) and ^{13}C (125.8 MHz) NMR spectra were recorded on a Bruker Avance 500 MHz NMR spectrometer. ^{1}H (600 MHz) and ^{13}C (150.9 MHz) NMR spectra were recorded on a Bruker Avance 400 MHz NMR spectrometer. HSQC and HMBC NMR spectra were also recorded on a Bruker Avance 300 NMR spectrometer, a Bruker Avance 400 NMR spectrometer, a Bruker Avance 500 NMR spectrometer or a Bruker Avance 600 NMR spectrometer. All spectra were recorded at 300 K in deuterated chloroform ($CDCl_3$) unless otherwise stated, using tetramethylsilane (TMS) as internal standard. Chemical shifts (δ_H and δ_C) are reported in parts per million (ppm) relative to TMS and coupling constants (*J*) are expressed in hertz (Hz). Splitting patterns in ^{1}H NMR spectra are designated as s (singlet), d (doublet), dd (doublet of doublets), t (triplet), q (quartet) and m (multiplet). ^{13}C NMR spectra were calibrated using the solvent signal, i.e., $CDCl_3$: δ_C 77.0 ppm, and multiplicities were assigned with the aid of DEPT experiments. Assignment of ^{1}H NMR signals was aided using 2D NMR experiments including ^{1}H−^{1}H COSY, HSQC, and HMBC as appropriate.

Infrared spectra were measured using a Perkin–Elmer UATR2 spectrometer. Flash column chromatography was carried out using Kieselgel silica gel 60, 0.040−0.063 mm (Merck). Thin-layer chromatography (TLC) was carried out on precoated silica gel plates (Merck 60 PF254). Visualization was achieved by UV (254 nm) light absorption or by staining using *p*-anisaldehyde solution.

Solutions in acetonitrile were employed for either LRMS or HRMS. Low-resolution mass spectra (LRMS) were recorded on a Waters Quattro Micro triple quadrupole instrument in electrospray ionization (ESI) mode using 50% acetonitrile−water containing 0.1% formic acid as eluent. High-resolution mass spectra (HRMS) were recorded on a Waters LCT Premier Tof LC−MS instrument in electrospray ionization (ESI) mode using 50% acetonitrile−water containing 0.1% formic acid as eluent. High resolution (precise) mass spectra (HRMS) was also recorded on a Waters Vion IMS instrument (SAA055K) with Waters Acquity I-class UPLC in electrospray ionization (ESI) mode using 50%

acetonitrile–water containing 0.1% formic acid as eluent and Leucine Enkephalin as reference solution. Melting points were obtained using a Unimelt Thomas-Hoover Capillary melting point apparatus and, Stuart SMP11 melting point apparatus and are uncorrected.

1.3 Continuous Flow Details and Specifications

All continuous processes were performed using a Vapourtec R-Series flow chemistry system consisting of four HPLC pumps and up to four temperature controlled tubular reactors (glass reactor manifold containing a temperature-controlled glass column). All reaction tubing, coils, inlets, and connections were purged with reaction solvent prior to operation. All pumps were primed, and pump backwash reservoirs were filled using appropriate solvents. The solvent that was to be used was flushed through all injectors and reactors. Pumps were run at reaction flow rates to check for stability, in both reagent and solvent lines, before committing reagents. Reactors that were to be used were then heated to the desired temperatures, using the flow chemistry platform, to check system pressurisation.

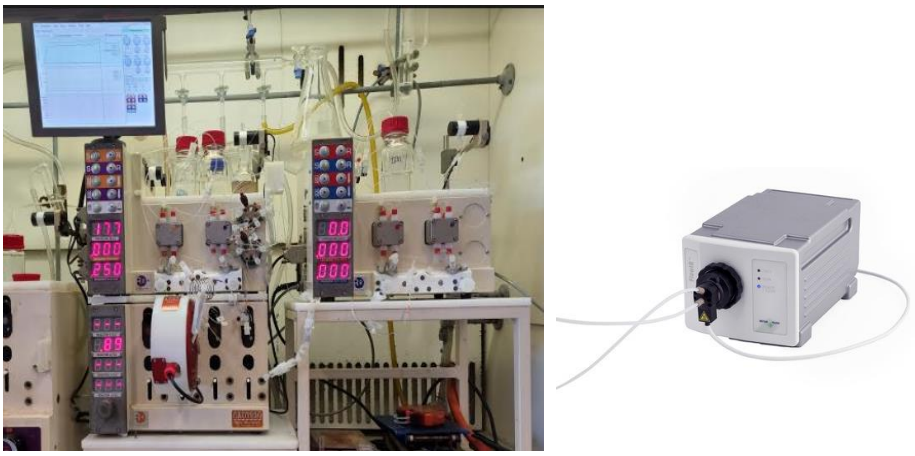

Figure S1. Continuous flow equipment utilised in this work; Vapourtec R-series and Mettler–Toledo FlowIR

General specifications for the continuous flow reactor	
Material of tubing	PFA<br>(stainless steel – high temperature reactor coil)
Diameter of tubing	1 mm
Working flow rates	0.05 mL/min to 9.99 mL/min
Pressure	Max 42 bar
Temperature range	–70 °C to 250 °C

2. Synthesis and Characterization of *N*-Boc Amines **8a-n**

The series of *N*-Boc protected amines **8a-n** were synthesised according to literature procedures; in each case the spectral characteristics agreed with the literature data.

General procedure C[1]: Di-*tert*-butyl-dicarbonate (1.1 equiv) was added dropwise to a stirring solution of amine (1.0 equiv) at 0 °C with triethylamine (1.0 equiv) in methanol (0.3 M solution) under nitrogen atmosphere. The resulting solution was stirred at room temperature with TLC monitoring. Following completion of the reaction, the solvent was removed by rotary evaporator and the resulting residue was dissolved in dichloromethane (1 × 10 mL/mmol). The organic layer was washed with aqueous HCl (1 M, 1 × 10 mL/mmol), water (1 × 15 mL/mmol) and brine (1 × 15 mL/mmol), dried over sodium sulfate, filtered, and concentrated in vacuo to give the corresponding crude *N*-Boc protected product which, if necessary, was purified by column chromatography using hexane:ethyl acetate as eluent.

General procedure D[2]: Di-*tert*-butyl-dicarbonate (1.1 equiv) was added dropwise to a stirring solution of amine (1.0 equiv) at 0 °C with triethylamine (1.0 equiv) and DMAP (10 mol%) in acetonitrile (0.3 M solution) under nitrogen atmosphere. The resulting solution was stirred at room temperature with TLC monitoring. Following completion of the reaction, the solvent was removed by rotary evaporator and the resulting residue was dissolved in dichloromethane (1 × 10 mL/mmol). The organic layer was washed with aqueous HCl (1 M, 1 × 10 mL/mmol), water (1 × 15 mL/mmol) and brine (1 × 15 mL/mmol), dried over sodium sulfate, filtered, and concentrated in vacuo to give the corresponding crude *N*-Boc protected product which, if necessary was purified by column chromatography using hexane:ethyl acetate as eluent.

General procedure E[3]: Di-*tert*-butyl-dicarbonate (1.1 equiv) was added dropwise to a stirring solution of amino acid (1.0 equiv) at 0 °C with triethylamine (1.0 equiv) in water:1,4-dioxane (1:1, 0.3 M solution) under nitrogen atmosphere. The resulting solution was stirred at room temperature with TLC monitoring. Following completion of the reaction, the solvent was removed by rotary evaporator and the resulting residue was dissolved in water (1 × 20 mL/mmol) and washed with ethyl acetate (2 × 10 mL/mmol). The aqueous layer was then acidified using aqueous HCl (2 M, ~5 mL/mmol). The aqueous layer was then extracted with ethyl acetate (3 × 10 mL/mmol), dried with sodium sulfate, filtered, and evaporated in vacuo to obtain the *N*-Boc protected amino acid.

tert-Butyl phenethylcarbamate (8a)[4]

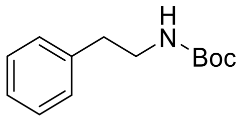

This compound was prepared according to the *General Procedure C* using phenethylamine (1.23 g, 10.17 mmol, 1.0 equiv), di-*tert*-butyl-dicarbonate (2.44 g, 11.19 mmol, 1.1 equiv) and triethylamine (1.42 mL, 10.17 mmol, 1.0 equiv) in methanol (35 mL) for a reaction time of 2 h.

White crystalline solid (2.11 g, 94%). mp 55–56 °C (lit.[4] 54.2–54.6 °C). υ_{max}/cm^{-1} (ATR): 3371 (NH), 1701 (C=O).

δ_H (400 MHz, $CDCl_3$): 1.43 [9H, s, C(C*H*$_3$)$_3$], 2.79 [2H, t, *J* 7.0 Hz, C*H*$_2$], 3.27–3.33 [2H, m, C*H*$_2$NH], 4.59 [1H, bs, N*H*], 7.16–7.34 [5H, m, aromatic C*H*].

δ_C (100 MHz, $CDCl_3$): 28.4 [CH$_3$, C(*C*H$_3$)$_3$], 36.2 [CH$_2$, *C*H$_2$], 41.8 [CH$_2$, *C*H$_2$NH], 79.2 [C, *C*(CH$_3$)$_3$], 126.4 [CH, aromatic *C*H], 128.6 [CH, 2 × aromatic *C*H], 128.8 [CH, 2 × aromatic *C*H], 139.0 [C, aromatic *C*], 155.9 [C, *C*=O]. Spectroscopic characteristics were consistent with those reported in previous literature.[4]

tert-Butyl (4-methoxyphenyl)carbamate (8b)[4]

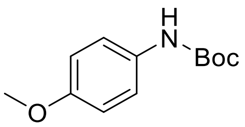

This compound was prepared according to the *General Procedure C* using *p*-anisidine (0.59 g, 4.7 mmol, 1.0 equiv), di-*tert*-butyl-dicarbonate (1.13 g, 5.17 mmol, 1.1 equiv) and triethylamine (0.65 mL, 4.7 mmol) in methanol (15 mL) for a reaction time of 3 h.

Grey crystalline solid (0.98 g, 93%). mp 93–95 °C (lit.[4] 94–96 °C). υ_{max}/cm^{-1} (ATR) 3364 (NH), 1692 (C=O).

δ_H (400 MHz, $CDCl_3$): 1.51 [9H, s, C(C*H*$_3$)$_3$], 3.77 [3H, s, OC*H*$_3$], 6.33 [1H, bs, N*H*], 6.78–6.88 [2H, d, *J* 9.0 Hz, 2 x aromatic C*H*], 7.21–7.32 [2H, d, *J* 8.9 Hz, 2 × aromatic C*H*].

δ_c (100 MHz, $CDCl_3$): 28.4 [CH$_3$, C(*C*H$_3$)$_3$], 55.5 [CH$_3$, O*C*H$_3$], 80.2 [C, *C*(CH$_3$)$_3$], 114.2 [CH, 2 × aromatic *C*H], 120.6 [CH, 2 × aromatic *C*H], 131.4 [C, aromatic *C*], 153.2, 155.7 [2 × C, *C*=O and *C*OCH$_3$]. Spectroscopic characteristics were consistent with those reported in previous literature.[4]

tert-Butyl (4-chlorophenyl)carbamate (8c)[4]

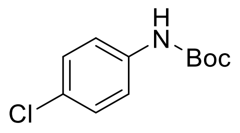

This compound was prepared according to the *General Procedure D* using 4-chloroaniline (0.88 g, 6.9 mmol, 1.0 equiv), di-*tert*-butyl-dicarbonate (1.66 g, 7.59 mmol, 1.1 equiv), triethylamine (0.96 mL, 6.9 mmol. 1.0 equiv) and DMAP (0.08 g, 0.69 mmol, 0.1 equiv) in methanol (25 mL) for a reaction time of 3 h. The crude product was purified by flash chromatography on silica gel using hexane/ethyl acetate (9:1) as eluent.

Beige crystalline solid (1.41 g, 90%). mp 109–111 °C (lit.[4] 111–113 °C). υ_{max}/cm^{-1} (ATR) 3365 (NH), 1693 (C=O).

δ_H (300 MHz, $CDCl_3$): 1.51 [9H, s, C(C*H*$_3$)$_3$], 6.52 [1H, bs, N*H*], 7.19–7.34 [4H, m, aromatic C*H*].

δ_C (75 MHz, $CDCl_3$): 28.3 [CH_3, C(*C*H$_3$)$_3$], 80.5 [C, *C*(CH$_3$)$_3$], 119.7 [CH, 2 × aromatic *C*H], 127.9 [CH, 2 × aromatic *C*H], 128.9 [C, aromatic *C*], 136.9 [C, *C*Cl], 152.6 [C, *C*=O]. Spectroscopic characteristics were consistent with those reported in previous literature.[4]

tert-Butyl phenylcarbamate (8d)[4]

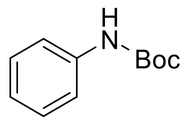

This compound was prepared according to the *General Procedure C* using aniline (0.53 g, 5.7 mmol, 1.0 equiv), di-*tert*-butyl-dicarbonate (1.36 g, 6.27 mmol, 1.1 equiv) and triethylamine (0.79 mL, 5.7 mmol, 1.0 equiv) in methanol (20 mL) for a reaction time of 3 h.

White crystalline solid (1.01 g, 92%). mp 134–136 °C (lit.[4] 132–133 °C). υ_{max}/cm^{-1} (ATR) 3369 (NH), 1692 (C=O).

δ_H (400 MHz, $CDCl_3$): 1.51 [9H, s, C(C*H*$_3$)$_3$], 6.55 [1H, bs, N*H*], 7.01 [1H, t, *J* 7.3 Hz, aromatic C*H*], 7.22–7.37 [4H, m, 4 × aromatic C*H*].

δ_C (100 MHz, $CDCl_3$): 28.4 [CH_3, C(*C*H$_3$)$_3$], 80.5 [C, *C*(CH$_3$)$_3$], 118.6 [CH, 2 × aromatic *C*H], 123.0 [CH, aromatic 2 × *C*H], 129.0 [CH, aromatic *C*H], 138.4 [C, aromatic *C*], 152.8 [C, *C*=O]. Spectroscopic characteristics were consistent with those reported in previous literature.[4]

tert-Butyl methyl(phenethyl)carbamate (8e)[5]

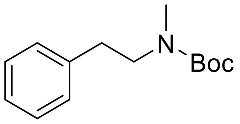

This compound was prepared according to the *General Procedure C* using *N*-methylphenethylamine (1.35 g, 9.99 mmol, 1.0 equiv), di-*tert*-butyl carbonate (2.40 g, 10.99 mmol, 1.1 equiv) and triethylamine (1.39 mL, 9.99 mmol, 1.0 equiv) in methanol (30 mL) for a reaction time of 3 h.
Pale-yellow oil (2.30 g, 98%). υ_{max}/cm^{-1} (ATR) 1699 (C=O), 1118 (C–O).
δ_H (400 MHz, $CDCl_3$, peak broadening of signals 1.39, 2.77 and 3.40 due to rotamers): 1.39–1.45 [9H, s, C(C*H*$_3$)$_3$], 2.75–2.81 [5H, m, C*H*$_2$ and C*H*$_3$], 3.39–3.45 [2H, m, C*H*$_2$], 7.14–7.31 [5H, m, aromatic C*H*].
δ_C (100 MHz, $CDCl_3$, evidence of rotamers at signals 34.6 and 50.8): 28.3 [CH_3, C(*C*H$_3$)$_3$], 34.2 [CH_2, *C*H$_2$], 34.6 [CH_3, *C*H$_3$], 50.8 [CH_2, *C*H$_2$], 79.1 [C, *C*(CH$_3$)$_3$], 126.2 [CH, aromatic *C*H], 128.4 [CH, 2 × aromatic *C*H], 128.8 [CH, 2 × aromatic *C*H], 139.2 [C, aromatic *C*], 155.5 [C, *C*=O]. Spectroscopic characteristics were consistent with those reported in previous literature.[5]

tert-Butyl morpholine-4-carboxylate (8f)[6]

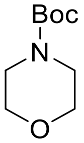

1

This compound was prepared according to the *General Procedure C* using morpholine (0.88 mL, 10.10 mmol, 1.0 equiv), di-*tert*-butyl-dicarbonate (2.42 g, 11.11 mmol, 1.1 equiv) and triethylamine (1.41 mL, 10.10 mmol, 1.0 equiv) in methanol (30 mL) for a reaction time of 1 h. The crude product was purified by flash chromatography on silica gel using hexane/ethyl acetate (9:1) as eluent.
White crystalline solid (1.70 g, 90%). mp 64–66 °C (lit.[6] 65–66 °C). υ_{max}/cm^{-1} (ATR) 1702 (C=O), 1111 (C–O).
δ_H (300 MHz, $CDCl_3$): 1.47 [9H, s, C(C*H*$_3$)$_3$], 3.35–3.45 [4H, m, 2 × C*H*$_2$], 3.59–3.68 [4H, m, 2 × C*H*$_2$].
δ_c (75 MHz, $CDCl_3$): 28.4 [CH_3, C(*C*H$_3$)$_3$], 43.9 [CH_2, br, 2 × *C*H$_2$], 66.6 [CH_2, 2 × *C*H$_2$], 79.8 [C, *C*(CH$_3$)$_3$], 155.6 [C, *C*=O]. Spectroscopic characteristics were consistent with those reported in previous literature.[6]

tert-Butyl piperidine-1-carboxylate (8g)[4]

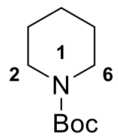

This compound was prepared according to the *General Procedure C* using piperidine (0.19 g, 2.24 mmol, 1.0 equiv), di-*tert*-butyl-dicarbonate (0.54 g, 2.46 mmol, 1.1 equiv) and triethylamine (0.31 mL, 2.24 mmol, 1.0 equiv) in methanol (10 mL) for a reaction time of 1 h.

Colourless oil (0.38 g, 93%). υ_{max}/cm^{-1} (ATR) 1702 (C=O), 1161 (C–O).

δ_H (300 MHz, $CDCl_3$): 1.45–1.60 [15H, m, C(C*H*$_3$)$_3$ and C(3)*H*$_2$, C(4)*H*$_2$, C(5)*H*$_2$], 3.33–3.41 [4H, m, C(2)*H*$_2$, C(6)*H*$_2$].

δ_C (75 MHz, $CDCl_3$): 24.5 [CH_2, *C*H$_2$], 25.7 [CH_2, 2 × *C*H$_2$], 28.4 [CH_3, C(*C*H$_3$)$_3$], 44.6 [CH_2, 2 × *C*H$_2$], 79.0 [C, *C*(CH$_3$)$_3$], 154.9 [C, *C*=O]. Spectroscopic characteristics were consistent with those reported in previous literature.[4]

tert-Butyl 3,4-dihydroisoquinoline-2(_1H_)-carboxylate (8h)[7]

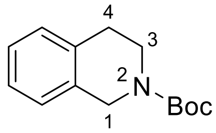

This compound was prepared according to the *General Procedure C* using 1,2,3,4,-tetrahydroisoquinoline (1.32 g, 9.91 mmol, 1.0 equiv), di-*tert*-butyl-dicarbonate (2.38 g, 10.90 mmol, 1.1 equiv), triethylamine (1.38 g, 9.91 mmol, 1.0 equiv) in methanol (30 mL) for a reaction time of 16 h. The crude product was purified by flash chromatography on silica gel using hexane/ethyl acetate (9:1) as eluent.

Yellow oil (2.08 g, 90%). υ_{max}/cm^{-1} (ATR) 1698 (C=O), 1173 (C–O).

δ_H (400 MHz, $CDCl_3$, peak broadening of signals at 1.49, 2.82 and 3.62 due to rotamers): 1.49 [9H, s, C(C*H*$_3$)$_3$], 2.75–2.87 [2H, m, C*H*$_2$], 3.59–3.67 [2H, m, C*H*$_2$], 4.56 [2H, s, C*H*$_2$], 7.04–7.19 [4H, m, 4 × aromatic C*H*].

δ_C (100 MHz, $CDCl_3$, evidence of rotamers for signals 28.5, 40.7 and 45.3): 28.5 [CH_3, C(*C*H$_3$)$_3$], 29.0 [CH_2, *C*H$_2$], 40.7 and 41.9 [CH_2, *C*H$_2$], 45.3 and 46.0 [CH_2, *C*H$_2$], 79.7 [C, *C*(CH$_3$)$_3$], 126.2 [CH, aromatic *C*H], 126.4 [CH, 2 × aromatic *C*H], 128.8 [CH, aromatic *C*H], 133.6 [C, aromatic *C*], 134.9 [C, aromatic *C*], 154.9 [C, *C*=O]. Spectroscopic characteristics were consistent with those reported in previous literature.[7]

tert-Butyl 3,4-dihydroquinoline-1(2_H_)-carboxylate (8i)[8]

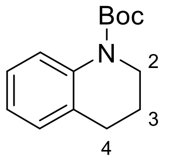

This compound was prepared according to the *General Procedure D* using 1,2,3,4,-tetrahydroquinoline (0.40 g, 3.10 mmol, 1.0 equiv), di-*tert*-butyl-dicarbonate (0.74 g, 3.41 mmol, 1.1 equiv), triethylamine (0.43 mL, 3.10 mmol, 1.0 equiv), DMAP (0.04 g, 0.31 mmol, 0.1 equiv) in methanol (10 mL) for a reaction time of 12 h. The crude product was purified by flash chromatography on silica gel using hexane/ethyl acetate (9:1) as eluent.

Pale yellow oil (0.60g, 83%). υ_{max}/cm^{-1} (ATR) 1690 (C=O), 1155 (C–O).

δ_H (300 MHz, $CDCl_3$): 1.49 [9H, s, C(CH_3)$_3$], 1.80 [2H, quintet, *J* 6.4 Hz, CH_2], 2.63 [2H, t, *J* 6.5 Hz, CH_2], 3.58-3.69 [2H, m, CH_2], 6.83–7.12 [3H, m, 3 × aromatic C*H*], 7.66 [1H, d, *J* 8.0 Hz, aromatic C*H*].

δ_C (75 MHz, $CDCl_3$): 23.6 [CH_2, *C*H_2], 27.5 [CH_2, *C*H_2], 28.3 [CH_3, C(*C*H_3)$_3$], 44.7 [CH_2, *C*H_2], 80.3 [C, *C*(CH_3)$_3$], 123.1 [CH, aromatic *C*H], 124.1 [CH, aromatic *C*H], 125.6 [CH, aromatic *C*H], 128.5 [CH, aromatic *C*H], 129.7 [C, aromatic *C*], 138.6 [C, aromatic *C*], 153.7 [C, *C*=O]. Spectroscopic characteristics were consistent with those reported in previous literature.[8]

tert-Butyl methyl(phenyl)carbamate (8j)[9]

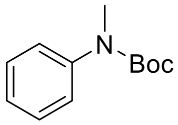

This compound was prepared according to the *General Procedure C* using *N*-methylaniline (0.71 g, 6.7 mmol, 1.0 equiv), di-*tert*-butyl-dicarbonate (1.61 g, 7.4 mmol, 1.1 equiv), triethylamine (0.93 mL, 6.7 mmol, 1.0 equiv) and DMAP (0.08 g, 0.67 mmol, 0.1 equiv) in methanol (20 mL) for a reaction time of 3 h. The crude product was purified by flash chromatography on silica gel using hexane/ethyl acetate (9:1) as eluent.

Pale yellow oil (1.18 g, 85%). υ_{max}/cm^{-1} (ATR) 1693 (C=O), 1117 (C–O).

δ_H (400 MHz, $CDCl_3$): 1.36 [9H, s, C(CH_3)$_3$], 3.16 [3H, s, NCH_3], 7.03–7.26 [5H, m, aromatic C*H*].

δ_C (100 MHz, $CDCl_3$): 28.3 [CH_3, C(*C*H_3)$_3$], 37.3 [CH_2, N*C*H_3], 80.2 [C, *C*(CH_3)$_3$], 125.4 [CH, 2 × aromatic *C*H], 125.5 [CH, aromatic *C*H], 128.6 [CH, 2 × aromatic *C*H], 143.8 [C, aromatic *C*], 157.8 [C, *C*=O]. Spectroscopic characteristics were consistent with those reported in previous literature.[9]

tert-Butyl 1H-imidazole-1-carboxylate (8k)[6]

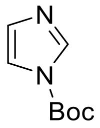

This compound was prepared according to the *General Procedure C* using imidazole (0.75 g, 10.99 mmol, 1.0 equiv), di-*tert*-butyl-dicarbonate (2.63 g, 12.09 mmol, 1.1 equiv) and triethylamine (1.53 mL, 10.99 mmol, 1.0 equiv) in methanol (35 mL) for a reaction time of 1 h. The crude product was purified by flash chromatography on silica gel using hexane/ethyl acetate (9:1) as eluent.
White crystalline solid (0.59 g, 79%). mp 43–45 °C (lit.[6] 45–47 °C). υ_{max}/cm^{-1} (ATR) 1689 (C=O), 1206 (C–O).
δ_H (400 MHz, $CDCl_3$): 1.63 [9H, s, C(C*H*$_3$)$_3$], 7.04 [1H, s, C*H*], 7.38 [1H, s, C*H*], 8.08 [1H, s, C*H*].
δ_c (100 MHz, $CDCl_3$): 27.8 [CH_3, C(CH_3)$_3$], 85.6 [C, *C*(CH_3)], 117.0 [CH, aromatic *C*H], 130.0 [CH, aromatic *C*H], 137.0 [CH, aromatic *C*H], 147.1 [C, *C*=O]. Spectroscopic characteristics were consistent with those reported in previous literature.[6]

tert-Butyl 1H-indole-1-carboxylate (8l)[10]

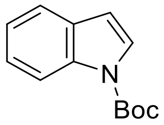

This compound was prepared according to the *General Procedure C* using indole (0.59 g, 5.12 mmol, 1.0 equiv), di-*tert*-butyl-dicarbonate (1.23 g, 5.63 mmol, 1.1 equiv) and triethylamine (0.71 mL, 5.12 mmol, 1.0 equiv) in methanol (15 mL) for a reaction time of 1 h. The crude product was purified by flash chromatography on silica gel using hexane/ethyl acetate (9:1) as eluent.
Colourless oil (1.02 g, 90%). υ_{max}/cm^{-1} (ATR) 1788 (C=O).
δ_H (300 MHz, $CDCl_3$): 1.69 [9H, s, C(C*H*$_3$)$_3$], 6.54–6.68 [1H, m, aromatic C*H*], 7.29–7.38 [2H, m, 2 × aromatic C*H*], 7.50–7.61 [2H, m, 2 × aromatic C*H*], 8.16 [1H, d, *J* 8.0 Hz, aromatic C*H*].
δ_c (75 MHz, $CDCl_3$): 28.3 [CH_3, C(*C*H$_3$)$_3$], 83.5 [C, *C*(CH_3)], 106.9 [CH, aromatic *C*H], 115.7 [CH, aromatic *C*H], 121.0 [CH, aromatic *C*H], 122.2 [CH, aromatic *C*H], 124.3 [CH, aromatic *C*H], 126.0 [CH, aromatic *C*H], 130.7 [C, aromatic *C*], 135.3 [C, aromatic *C*], 149.1 [C, *C*=O]. Spectroscopic characteristics were consistent with those reported in previous literature.[10]

tert-Butoxycarbonyl glycine (8m)[11]

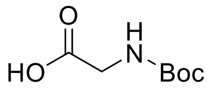

This compound was prepared according to the *General Procedure E* using glycine (0.88 g, 5.01 mmol, 1.0 equiv), di-*tert*-butyl dicarbonate (1.20 g, 5.51 mmol, 1.1 equiv) and triethylamine (0.68 mL, 5.01 mmol, 1.0 equiv) in 1:1 water/1,4-dioxane (20 mL) for a reaction time of 1 h.

White crystalline solid (0.87 g, 83%). mp 87–88 °C (lit.[11] 85–86 °C). υ_{max}/cm^{-1} (ATR) 3366 (NH), 3089 (CO_2H), 1715 (C=O), 1706 (C=O).

δ_H (400 MHz, $CDCl_3$, mixture of rotamers): 1.45 [9H, s, C(C*H*$_3$)$_3$], 3.93–3.96 [2H, m, C*H*$_2$], 5.32 (0.63H) and 6.47 (0.33H) [1H, 2 × br s, rotamers, N*H*], 7.65 [1H, bs, CO_2*H*].

δ_C (100 MHz, $CDCl_3$, mixture of rotamers): 28.2 [CH_3, C(*C*H$_3$)$_3$], 42.2 (major) and 43.3 (minor) [CH_2, *C*H$_2$], 80.4 (major) and 81.8 (minor) [C, *C*(CH$_3$)$_3$], 156.2 (major) and 157.2 (minor) [C, *C*=O], 173.7 (minor) and 174.1 (major) [C, *C*O$_2$H]. Spectroscopic characteristics were consistent with those reported in previous literature.[11]

(_tert_-Butoxycarbonyl)-_L_-phenylalanine (8n)[12]

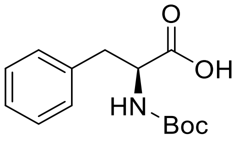

This compound was prepared according to the *General Procedure E* using *L*-phenylalanine (1.18 g, 7.13 mmol, 1.0 equiv), di-*tert*-butyl-dicarbonate (1.71 g, 7.84 mmol, 1.1 equiv) and triethylamine (0.99 mL, 7.13 mmol, 1.0 equiv) in 1:1 water/1,4-dioxane (25 mL) for a reaction time of 12 h.

White gummy solid (1.12 g, 59%). mp 61–63 °C (lit.[12] 62–65 °C). υ_{max}/cm^{-1} (ATR) 3371 (NH), 2985 (CO_2H), 1698 (C=O).

δ_H (300 MHz, $CDCl_3$, mixture of rotamers): 1.29 (4H) and 1.40 (5H) [9H, 2 × s, rotamers, C(C*H*$_3$)$_3$], 2.82–2.91 (0.4H) and 3.08–3.12 (0.6H) and 3.14–3.25 (1H) [2H, 3 × m, rotamers, C*H*$_2$], 4.34–4.40 (0.4H) and 4.59–4.67 (0.6H) [1H, 2 × m, rotamers, C*H*], 5.19–5.25 (0.6H) and 6.82–6.71 (0.4H) [1H, 2 × m, rotamers, N*H*], 7.06–7.21 [5H, m, aromatic C*H*], 11.29 [1H, br s, CO_2*H*].

δ_C (75 MHz, $CDCl_3$, mixture of rotamers): 28.0 (minor) and 28.3 (major) [CH_3, C(*C*H$_3$)$_3$], 37.9 (major) and 38.9 (minor) [CH_2,*C*H$_2$], 54.3 (major) and 56.3 (minor) [CH, *C*H], 80.2 (major) and 81.7 (minor) [C, *C*(CH$_3$)$_3$], 127.0 [CH, aromatic *C*H], 128.5 [CH, 2 × aromatic *C*H], 129.5 [CH, 2 × aromatic *C*H], 136.1 (major), 136.6 [C, aromatic *C*], 155.5 (major) and 156.8 (minor) [C, *C*=O], 175.7 [C, *C*O$_2$H]. Spectroscopic characteristics were consistent with those reported in previous literature.[12]

3. Synthesis of Diamines

Products were synthesised according to literature procedures and spectral data of known compounds agreed with the literature.

General method F[13]: Phenylhydrazine hydrochloride (1.0 equiv) was added in one portion to a stirring solution of cyclic or acyclic ketone derivative (1.2 equiv) in distilled ethanol under nitrogen. The reaction was stirred at 80 °C for 4–24 h after which it was cooled to room temperature and left overnight. The precipitate that formed was collected by filtration and dissolved in water (1 × 20 mL/mmol). The pH was adjusted to pH 10 using NaOH (2 M, ~10 mL/mmol). The resulting solid was then filtered, washed with chilled ethanol, and dried under vacuum to give the corresponding diamine derivatives.

2-(2-Methyl-1*H*-indol-3-yl)ethan-1-amine (11b)[14]

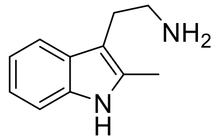

This compound was prepared according to *General Procedure F* using phenylhydrazine hydrochloride (1.45 g, 10 mmol, 1.0 equiv) and 5-chloro-2-pentanone (1.44 g, 12 mmol, 1.2 equiv) in ethanol (50 mL) for a reaction time of 6 h. The crude product was purified by flash chromatography on silica gel using hexane/ethyl acetate (9:1) as eluent.

Dark yellow oil (1.37 g, 79%). υ_{max}/cm^{-1} (ATR) 3381 (NH).

δ_H (400 MHz, $CDCl_3$): 1.67 [1H, bs, N*H*], 2.32 [3H, s, C*H₃*] 2.83 [2H, t, *J* 6.8 Hz, C*H*$_2$], 2.94 [2H, t, *J* 6.8 Hz, C*H*$_2$], 7.03–7.15 [2H, m, 2 × aromatic C*H*], 7.20 [1H, d, *J* 7.9 Hz, aromatic C*H*], 7.47 [1H, d, *J* 7.9 Hz, aromatic C*H*].

δ_c (100 MHz, $CDCl_3$): 11.7 [CH_3, *C*H_3], 28.1 [CH_2, *C*H_2], 42.6 [CH_2, *C*H_2], 108.9 [C, aromatic *C*], 110.4 [CH, aromatic *C*H], 117.9 [CH, aromatic *C*H], 119.1 [CH, aromatic *C*H], 120.9 [CH, aromatic *C*H], 128.7 [C, aromatic *C*], 132.0 [C, aromatic *C*], 135.4 [C, aromatic *C*]. Spectroscopic characteristics for the above compound were consistent with those reported in previous literature.[14]

***N*-Benzyl-2-(1*H*-indol-3-yl)ethane-1-amine (11c)[15]**

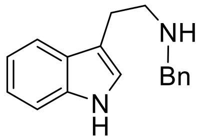

Tryptamine (0.55 g, 3.45 mmol, 1.0 equiv) was added to a stirring solution of benzaldehyde (0.35 g, 3.63 mmol, 1.0 equiv) in MeOH and stirred for 3 h. $NaBH_4$ (1.01 g, 5.2 mmol, 1.5 equiv) was then added in one portion and the reaction mixture was stirred for 3 h. The reaction mixture was then quenched

with H_2O (100 mL) and extracted with ethyl acetate (3 × 50 mL). The organic layers were combined, dried, and evaporated to obtain the crude residue. The crude product was purified by flash chromatography on silica gel using hexane/ethyl acetate (90:10) as eluent.

Yellow oil (0.85 g, 83%). υ_{max}/cm^{-1} (ATR): 3379 (NH).

δ_H (400 MHz, $CDCl_3$): 1.94 [br s, N*H*], 2.98 [4H, s, 2 × CH_2], 3.79 [2H, s, CH_2], 6.93 [1H, s, aromatic C*H*], 7.05–7.44 [8H, m, 8 × aromatic C*H*], 7.58 [1H, d, *J* 7.9 Hz, aromatic C*H*], 8.19 [1H, s, N*H*].

δ_C (100 MHz, $CDCl_3$, evidence of rotamers for signals 122.0, 126.9, 127.5): 25.8 [CH_2, *C*H_2], 49.7 [CH_2, *C*H_2], 53.9 [CH_2, *C*H_2], 111.2 [CH, aromatic *C*H], 113.9 [C, aromatic *C*], 118.9 [CH, aromatic *C*H], 119.3 [CH, aromatic *C*H], 122.0 [CH, aromatic *C*H], 126.9 [CH, aromatic *C*H], 127.5 [C, aromatic *C*], 128.2 [CH, aromatic *C*H], 128.4 [CH, aromatic *C*H], 128.6 [CH, aromatic *C*H], 136.5 [C, aromatic *C*], 140.2 [C, aromatic *C*]. Spectroscopic characteristics were consistent with those reported in previous literature.[15]

8-Chloro-2,3,4,5-tetrahydro-1*H*-pyrido[4,3-*b*]indole (11e)[13]

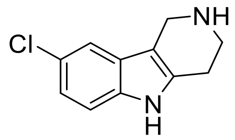

This compound was prepared according to *General Procedure F* using 4-chlorophenyl hydrazine hydrochloride (1.79 g, 10 mmol, 1.0 equiv) and 4-piperidone hydrochloride (1.84 g, 12 mmol, 1.2 equiv) in ethanol (50 ml) for 6 h. The resulting diamine product was used without further purification.

Off-white crystalline solid (0.76 g, 37%). mp 226–228 °C (lit.[13] 225–226 °C). υ_{max}/cm^{-1} (ATR) 3384 (NH).

δ_H (300 MHz, $CDCl_3$): 1.76 [1H, bs, N*H*], 2.43–2.50 [2H, m, CH_2], 3.14–3.23 [2H, m, CH_2], 3.57 [1H, bs, N*H*], 3.95 [2H, s, CH_2], 6.47–6.58 [2H, m, 2 × aromatic C*H*], 6.96 [1H, t, *J* 7.6 Hz, aromatic C*H*].

δ_c (75 MHz, $CDCl_3$): 24.5 [CH_2, *C*H_2], 41.9 [CH_2, *C*H_2], 43.3 [CH_2, *C*H_2] 108.6 [C, aromatic *C*], 112.5 [CH, aromatic *C*H], 116.9 [CH, aromatic *C*H], 120.2 [CH, aromatic *C*H], 123.3 [C, aromatic *C*], 127.2 [C, aromatic *C*], 133.9 [C, aromatic *C*], 135.9 [C, *C*-Cl]. Spectroscopic characteristics were consistent with those reported in previous literature. [13]

2,3,4,5-Tetrahydro-1*H*-pyrido[4,3-b]indole (11f)[13]

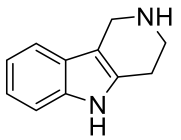

This compound was prepared according to *General Procedure F* using phenylhydrazine hydrochloride (1.45 g, 10 mmol, 1.0 equiv) and 4-piperidone hydrochloride (1.84 g, 12 mmol, 1.2 equiv) in ethanol (50 mL) for a reaction time of 12 h. The resulting diamine product was used without further purification.

Off-white crystalline solid (0.84 g, 49%). mp 212–214 °C (lit.[13] 215–216 °C). υ_{max}/cm^{-1} (ATR) 3374 (NH). δ_H (600 MHz, DMSO-d_6): 2.63–2.69 [2H, m, CH_2], 2.98–3.04 [2H, m, CH_2], 3.84 [2H, s, CH_2], 6.91 [1H, t, *J* 7.4 Hz, aromatic C*H*], 6.98 [1H, t, *J* 7.5 Hz, aromatic C*H*], 7.24 [1H, d, *J* 8.0 Hz, aromatic C*H*], 7.29 [1H, d, *J* 7.8 Hz, aromatic C*H*], 10.71 [1H, s, N*H*].

δ_c (150 MHz, DMSO-d_6): 24.6 [CH_2, *C*H_2], 42.2 [CH_2, *C*H_2], 43.5 [CH_2, *C*H_2], 108.7 [C, aromatic *C*], 111.1 [CH, aromatic *C*H], 117.5 [CH, aromatic *C*H], 118.5 [CH, aromatic *C*H], 120.5 [CH, aromatic *CH*], 126.1 [C, aromatic *C*], 133.8 [C, aromatic *C*], 135.8 [C, aromatic *C*]. Spectroscopic characteristics were consistent with those reported in previous literature.[13]

4. Synthesis and Characterization of Bis-Boc Diamines **9a, 9b, 9h-j**

Products were synthesised according to a modified literature procedure (see **Experimental** for details - *General Procedure A*).[16]

***tert*-Butyl 3-(2-((*tert*-butoxycarbonyl)amino)ethyl)-1*H*-indole-1-carboxylate (9a)[17]**

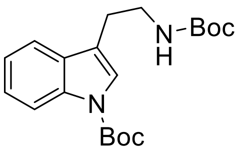

This compound was prepared according to *General Procedure A* using tryptamine (1.61 g, 5.0 mmol, 1 equiv), di-*tert*-butyl dicarbonate (2.40 g, 11 mmol, 2.2 equiv), DMAP (0.06 g, 0.5 mmol, 0.1 equiv) in MeCN (35 mL) for a reaction time of 16 h. The crude product was purified by flash chromatography on silica gel using dichloromethane/methanol (90:10) as eluent.

Yellow oil (2.96 g, 82%). υ_{max}/cm^{-1} (ATR) 3456 (NH), 1696 (C=O).

δ_H (300 MHz, $CDCl_3$): 1.44 [9H, s, NHCOC(CH_3)$_3$], 1.67 [9H, s, NCOC(CH_3)$_3$], 2.89 [2H, t, *J* 6.87 Hz, CH_2], 3.39–3.52 [2H, m, CH_2NH], 4.65 [1H, br s, N*H*], 7.19–7.26 [1H, m, aromatic C*H*], 7.26–7.36 [1H, m, aromatic C*H*], 7.41 [1H, s, aromatic C*H*], 7.52 [1H, d, *J* 7.5 Hz, aromatic C*H*], 8.41 [1H, d, *J* 8.1 Hz, aromatic C*H*].

δ_C (75 MHz, $CDCl_3$): 25.6 [CH_2, CH_2], 28.2, 28.4 [CH_3, 2 × C(CH_3)$_3$], 40.2 [CH_2, CH_2], 79.3 [C, *C*(CH_3)$_3$], 83.5 [C, *C*(CH_3)$_3$], 115.3 [CH, aromatic *C*H], 117.8 [C, aromatic *C*], 118.9 [CH, aromatic *C*H], 122.5 [CH, aromatic *C*H], 123.2 [CH, aromatic *C*H], 124.5 [CH, aromatic *C*H], 130.5 [C, aromatic *C*], 135.6 [C, aromatic *C*], 149.7 [C, *C*=O], 155.9 [C, *C*=O]. Spectroscopic characteristics were consistent with those reported in previous literature.[17]

***tert*-Butyl 3-(2-((*tert*-butoxycarbonyl)amino)ethyl)-2-methyl-1*H*-indole-1-carboxylate (9b)[17]**

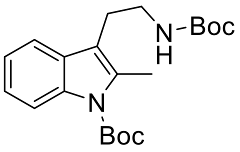

This compound was prepared according to *General Procedure A* using 2-methyl tryptamine (1.87 g, 5.0 mmol, 1.0 equiv), di-*tert*-butyl dicarbonate (2.40 g, 11 mmol, 2.2 equiv), DMAP (0.06 g, 0.5 mmol, 0.1 equiv) in MeCN (35 mL) for a reaction time of 16 h. The crude product was purified by flash chromatography on silica gel using dichloromethane/methanol (90:10) as eluent.

Yellow oil (2.96 g, 82%). υ_{max}/cm^{-1} (ATR) 3456 (NH), 1694 (C=O).

δ_H (300 MHz, $CDCl_3$): 1.44 [9H, s, NHCOC(CH_3)$_3$], 1.67 [9H, s, NCOC(CH_3)$_3$], 2.54 [3H, s, CH_3], 2.85–2.95 [2H, m, CH_2], 3.39–3.52 [2H, m, CH_2NH], 4.65 [1H, br s, N*H*], 7.18–7.29 [2H, m, 2 × aromatic C*H*], 7.36–7.46 [1H, m, aromatic C*H*], 8.14 [1H, d, *J* 8.1 Hz, aromatic C*H*].

δ_C (75 MHz, $CDCl_3$): 13.8 [CH_3, *C*H_3], 25.1 [CH_2, *C*H_2], 28.4 [CH_3, 2 × C(*C*H_3)$_3$], 40.4 [CH_2, C*H*$_2$], 79.3 [C, *C*(CH_3)$_3$], 83.6 [C, *C*(CH_3)$_3$], 116.1 [C, aromatic *C*], 115.6 [CH, aromatic *C*H], 117.8 [C, aromatic *C*], 122.5 [CH, aromatic *C*H], 123.2 [CH, aromatic *C*H], 128.9 [CH, aromatic *C*H], 134.5 [C, aromatic *C*], 135.7 [C, aromatic *C*], 150.7 [C, *C*=O], 155.9 [C, *C*=O].

Spectroscopic characteristics were consistent with those reported in previous literature.[17]

tert-Butyl (4-(2-((_tert_-butoxycarbonyl)amino)ethyl)phenyl)carbamate (9h)[4]

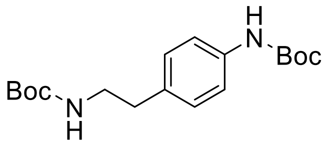

This compound was prepared according to *General Procedure A* using 4-(2-aminoethyl)aniline (0.70 g, 5.14 mmol, 1 equiv) and di-*tert*-butyl dicarbonate (2 × 1.23 g, 11.30 mmol, 2.2 equiv) in MeOH (50 mL) for a reaction time of 6 h. The crude product was purified by flash chromatography on silica gel using hexane/ethyl acetate (90:10 to 60:40) as eluent.

Colourless crystalline solid (1.38 g, 80%). mp 58–60 °C (lit.[4] 58–59 °C). υ_{max}/cm^{-1} (ATR) 3449 (NH), 1704 (C=O).

δ_H (300 MHz, $CDCl_3$): 1.43 [9H, s, aliphatic NHCOC(C*H*$_3$)$_3$], 1.52 [9H, s, NHCOC(C*H*$_3$)$_3$], 2.73 [2H, t, *J* 7.0 Hz, C*H*$_2$], 3.22–3.41 [2H, m, C*H*$_2$NH], 4.53 [1H, bs, N*H*], 6.52 [1H, bs, N*H*], 7.05–7.16 [2H, m, 2 × aromatic C*H*], 7.25–7.34 [2H, m, 2 × aromatic C*H*].

δ_c (75 MHz, $CDCl_3$): 28.3, 28.4 [CH_3, 2 × C(C*H*$_3$)$_3$], 35.4 [CH_2, *C*H_2], 41.8 [CH_2, NH*C*H_2], 79.2 [C, *C*(CH_3)$_3$], 80.4 [C, *C*(CH_3)$_3$], 118.9 [CH, 2 × aromatic *C*H], 129.2 [CH, 2 × aromatic *C*H], 133.6 [C, aromatic *C*], 136.7 [C, aromatic *C*], 152.8 [C, *C*=O], 155.8 [C, *C*=O]. Spectroscopic characteristics were consistent with those reported in previous literature.[4]

tert-Butyl 4-(2-((_tert_-butoxycarbonyl)amino)ethyl)piperazine-1-carboxylate (9i)[18]

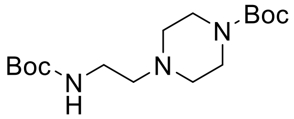

Di-*tert*-butyl dicarbonate (2.52 g, 11.57 mmol, 2.2 equiv) was added dropwise to a stirring solution of 2-(piperazin-1-yl)ethan-1-amine (0.68 g, 5.26 mmol, 1.0 equiv) and triethylamine (0.73 mL, 5.26 mmol, 1.0 equiv) in acetonitrile (30 mL) for a reaction time of 2 h. The crude product was purified by flash chromatography on silica gel using hexane/ethyl acetate (90:10) as eluent.

Colourless crystalline solid (1.64 g, 95%). υ_{max}/cm^{-1} (ATR) 3452 (NH), 1710 (C=O).

δ_H (400 MHz, $CDCl_3$): 1.46 [18H, s, NHCOC(C*H*$_3$)$_3$ and NHCOC(C*H*$_3$)$_3$], 2.36–2.44 [4H, m, 2 × C*H*$_2$], 2.46 [2H, t, *J* 6.9 Hz, C*H*$_2$], 3.20–3.25 [2H, m, C*H*$_2$NH], 3.40–3.46 [4H, m, 2 × C*H*$_2$], 4.98 [1H, s, N*H*].
δ_C (100 MHz, $CDCl_3$): 28.4 [*C*H$_3$, 2 × C(*C*H$_3$)$_3$], 37.1 [CH$_2$, br, *C*H$_2$], 43.6 [CH$_2$, br, 2 × *C*H$_2$], 52.8 [CH$_2$, 2 × *C*H$_2$], 57.22 [CH$_2$, *C*H$_2$], 79.2 [C, *C*(CH$_3$)$_3$], 79.6 [C, *C*(CH$_3$)$_3$], 154.7[C, *C*=O], 155.9[C, *C*=O].
Spectroscopic characteristics were consistent with those reported in previous literature.[18]

tert-Butyl 4-((_tert_-butoxycarbonyl)amino)piperidine-1-carboxylate (9j)[19]

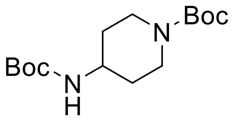

Di-*tert*-butyl dicarbonate (2.44 g, 11.20 mol, 2.2 equiv) was added dropwise to a stirring solution of piperidin-4-amine (0.51 g, 5.09 mmol, 1.0 equiv) and triethylamine (0.70 mL, 5.09 mmol, 1.0 equiv) in acetonitrile (30 mL) for a reaction time of 2 h. The crude product was purified by flash chromatography on silica gel using hexane/ethyl acetate (90:10) as eluent.
Colourless crystalline solid (1.39 g, 91%). υ_{max}/cm^{-1} (ATR) 3350 (NH), 1708 (C=O).
δ_H (400 MHz, $CDCl_3$): 1.16–1.29 [2H, m, 2 × one of C*H*$_2$], 1.41 [18H, s, NHCOC(C*H*$_3$)$_3$ and NHCOC(C*H*$_3$)$_3$], 1.81–1.91 [2H, m, 2 × one of C*H*$_2$], 2.81 [2H, t, *J* 11.8 Hz, 2 × one of C*H*$_2$], 3.55 [1H, br s, C*H*], 3.97 [2H, br s, 2 × one of C*H*$_2$], 4.47 [1H, br s, N*H*].
δ_c (100 MHz, $CDCl_3$): 28.4 [CH$_3$, 2 × C(*C*H$_3$)$_3$], 32.4 [CH$_2$, *C*H$_2$], 42.5 [CH$_2$, 2 × *C*H$_2$], 47.8 [CH, *C*H], 79.4 [C, *C*(CH$_3$)$_3$], 79.6 [C, *C*(CH$_3$)$_3$], 154.7 [C, *C*=O], 155.1 [C, *C*=O]. Spectroscopic characteristics were consistent with those reported in previous literature.[19]

5. Spectroscopic Data of Novel Bis-Boc Diamines **9c-g**

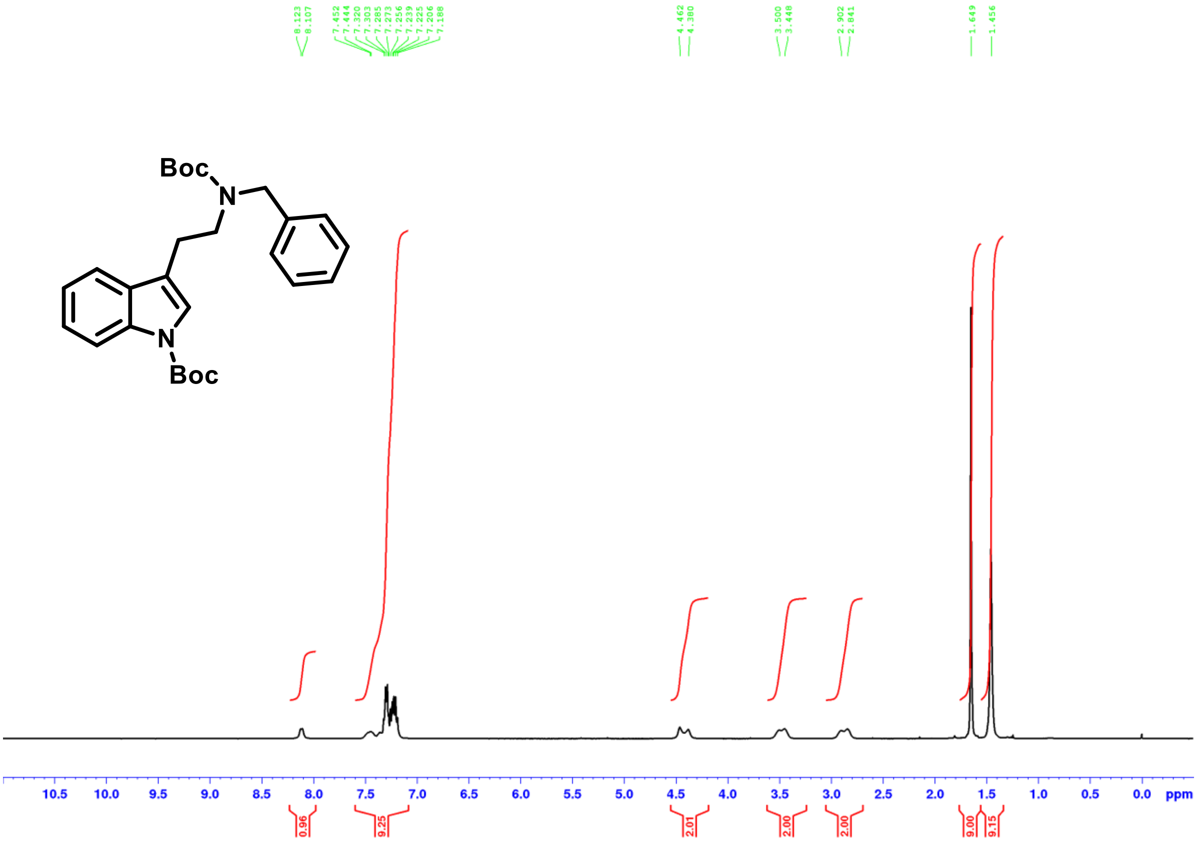

Figure S2. ^{1}H NMR ($CDCl_3$, 400 MHz) spectrum of bis-Boc compound **9c**

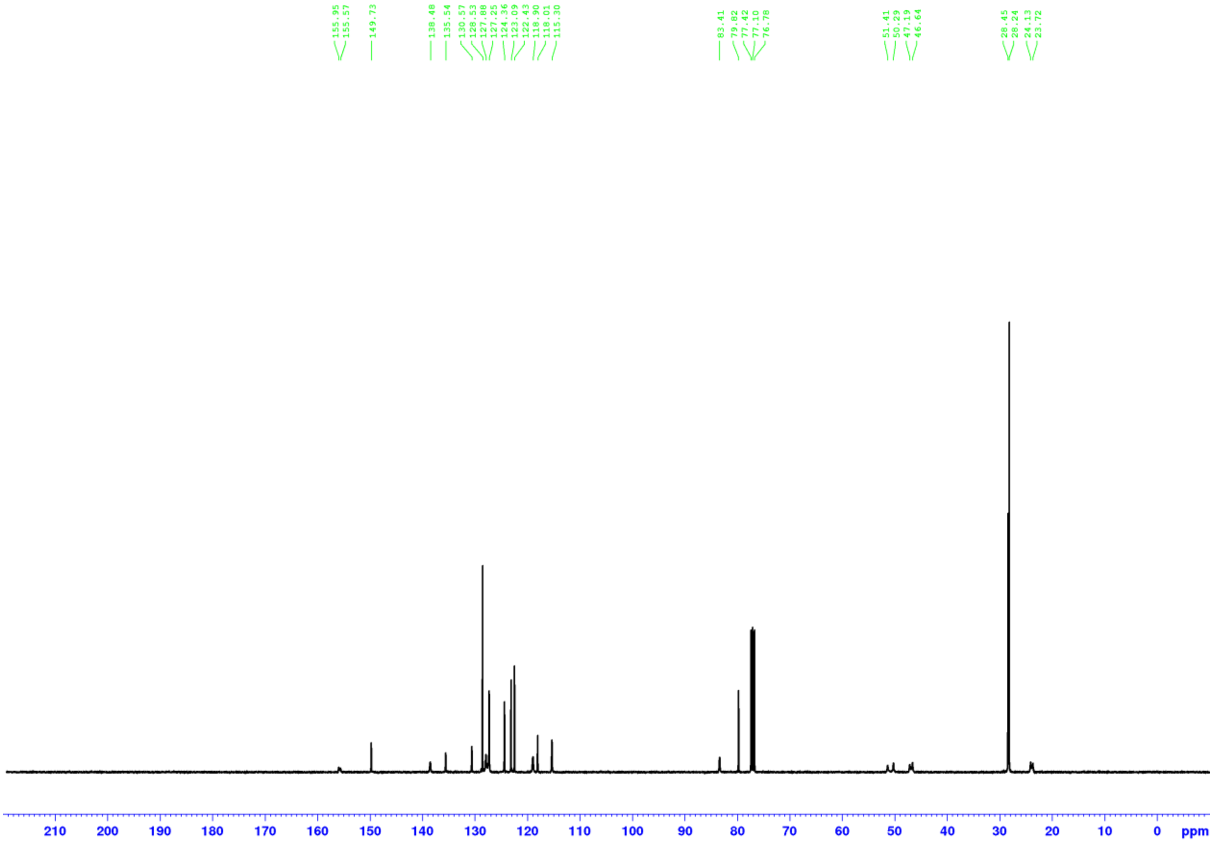

Figure S3. ^{13}C {1H} NMR ($CDCl_3$, 100 MHz) spectrum of bis-Boc compound **9c**

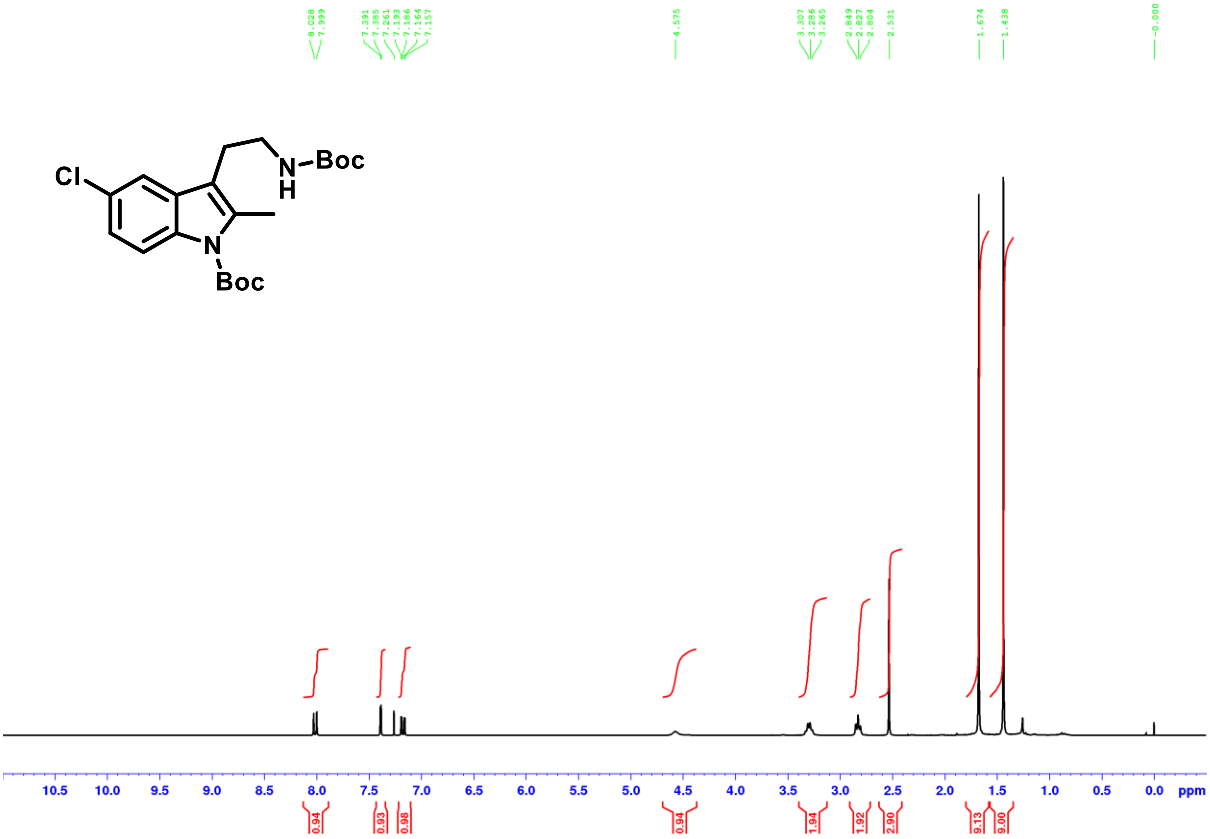

Figure S4. ^{1}H NMR ($CDCl_3$, 300 MHz) spectrum of bis-Boc compound **9d**

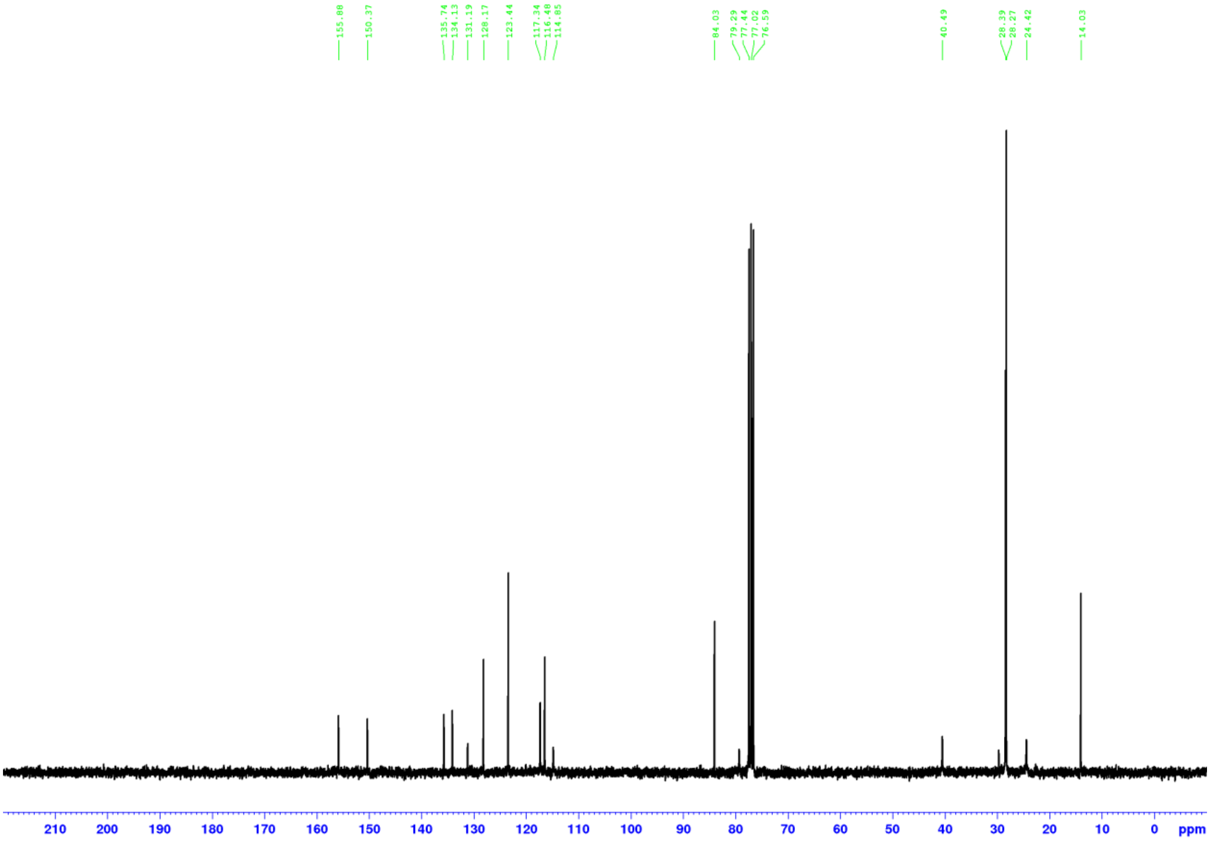

Figure S5. ^{13}C {1H} NMR ($CDCl_3$, 75 MHz) spectrum of bis-Boc compound **9d**

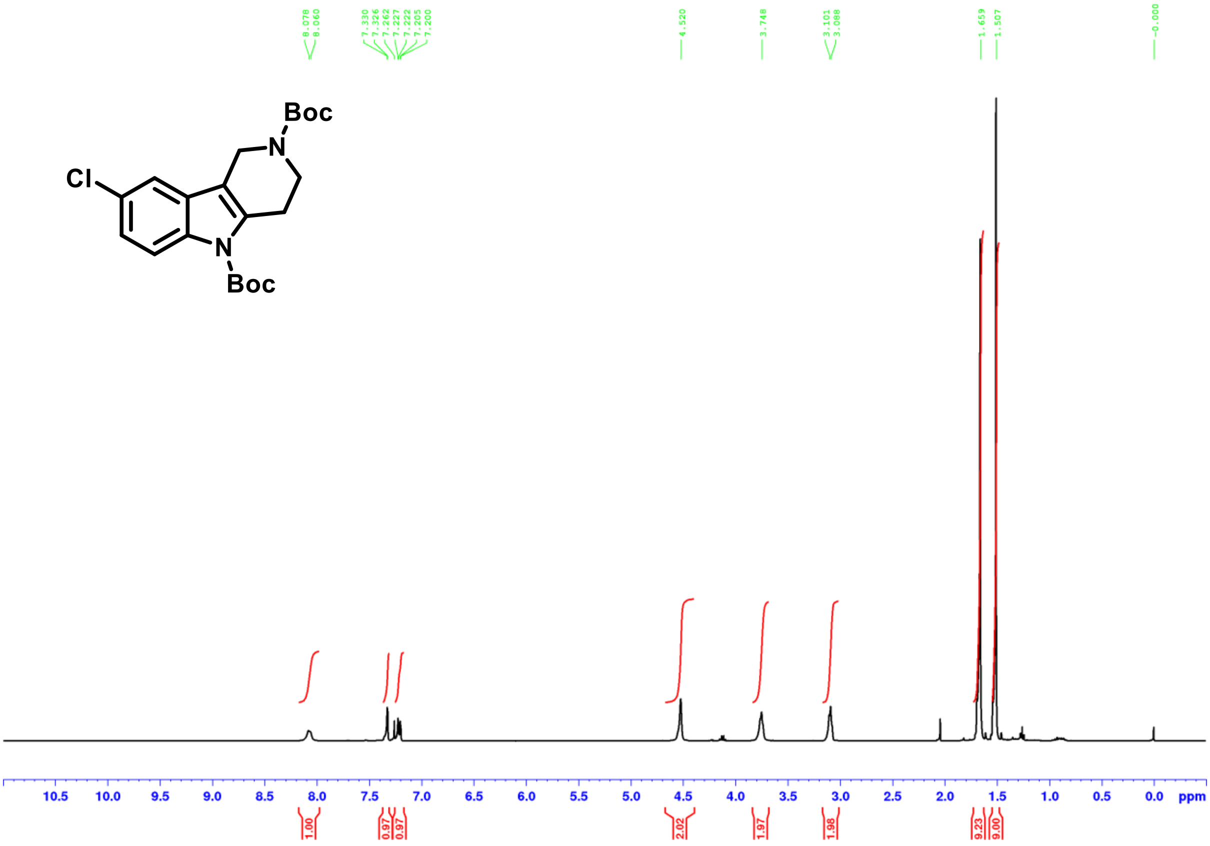

Figure S6. ^{1}H NMR ($CDCl_3$, 300 MHz) spectrum of bis-Boc compound **9e**

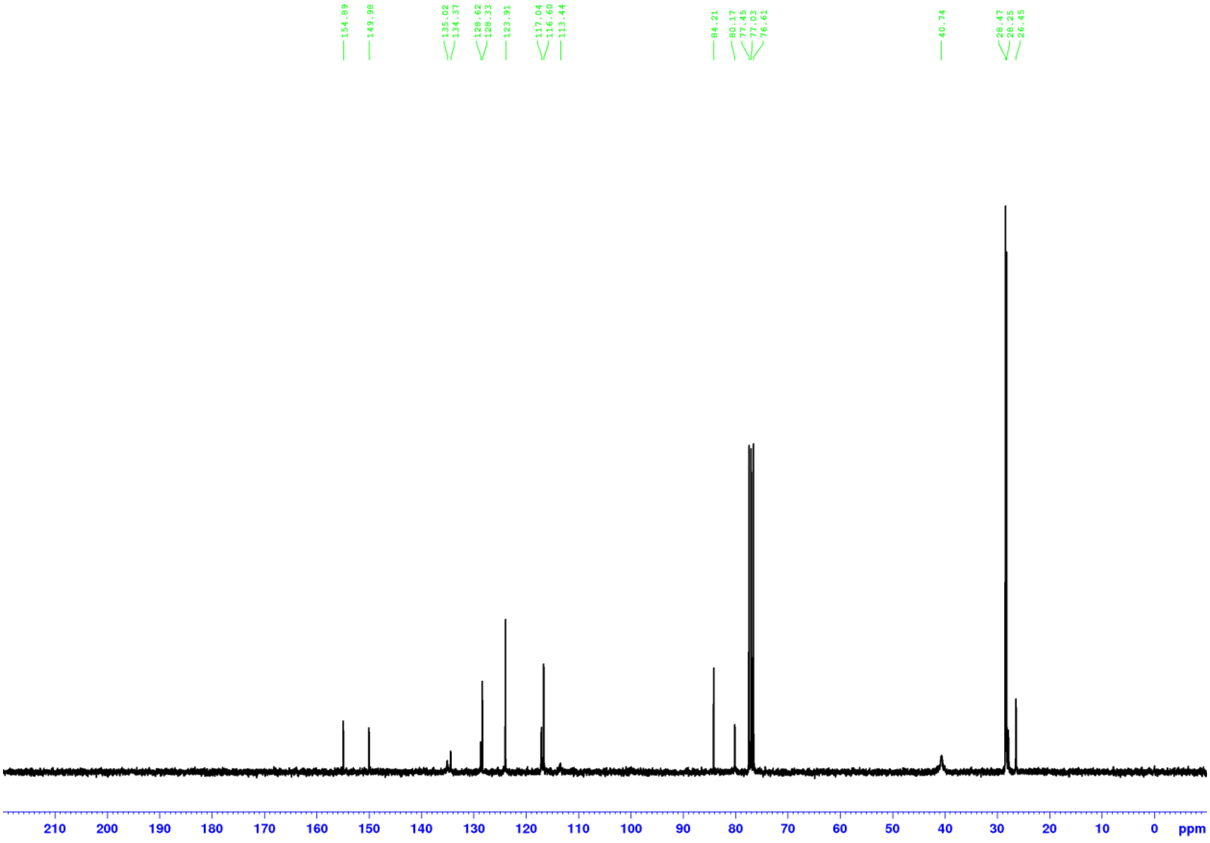

Figure S7. ^{13}C {1H} NMR ($CDCl_3$, 75 MHz) spectrum of bis-Boc compound **9e**

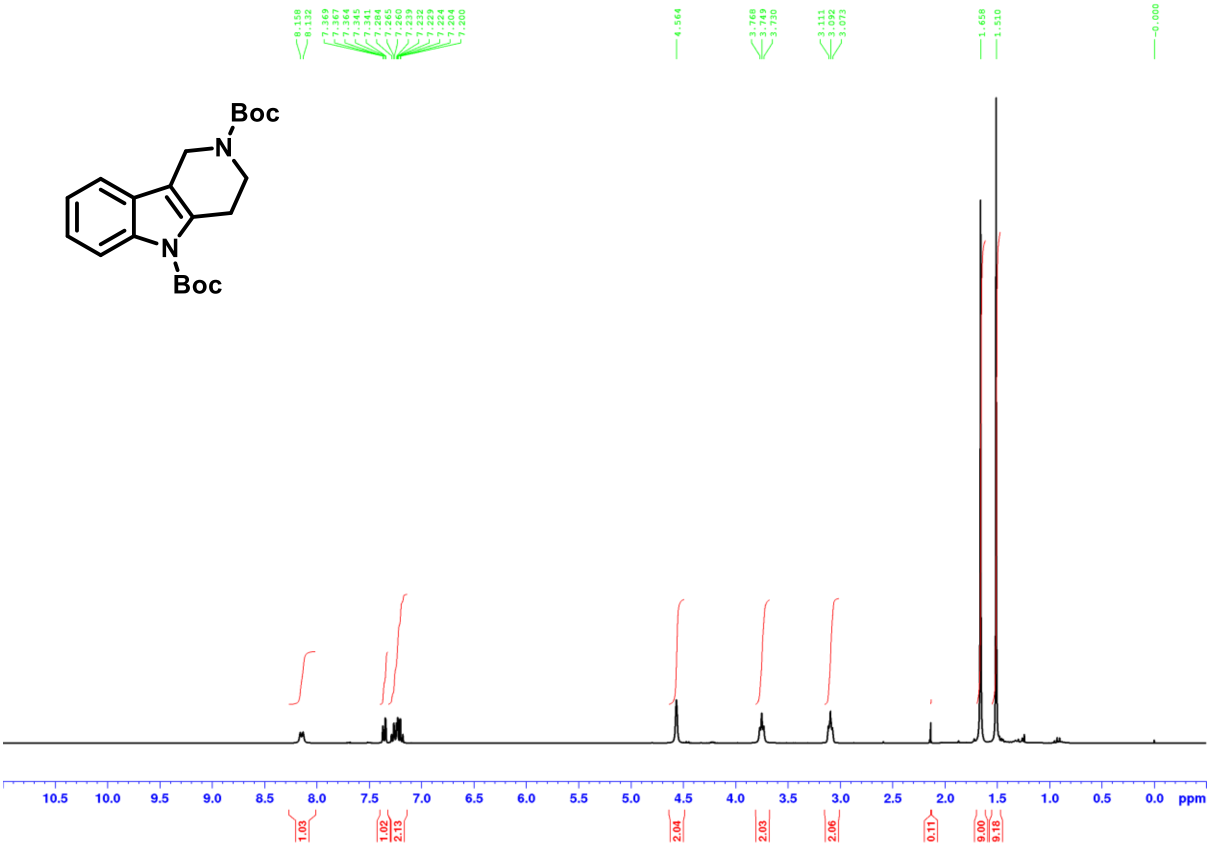

Figure S8. ^{1}H NMR ($CDCl_3$, 300 MHz) spectrum of bis-Boc compound **9f**

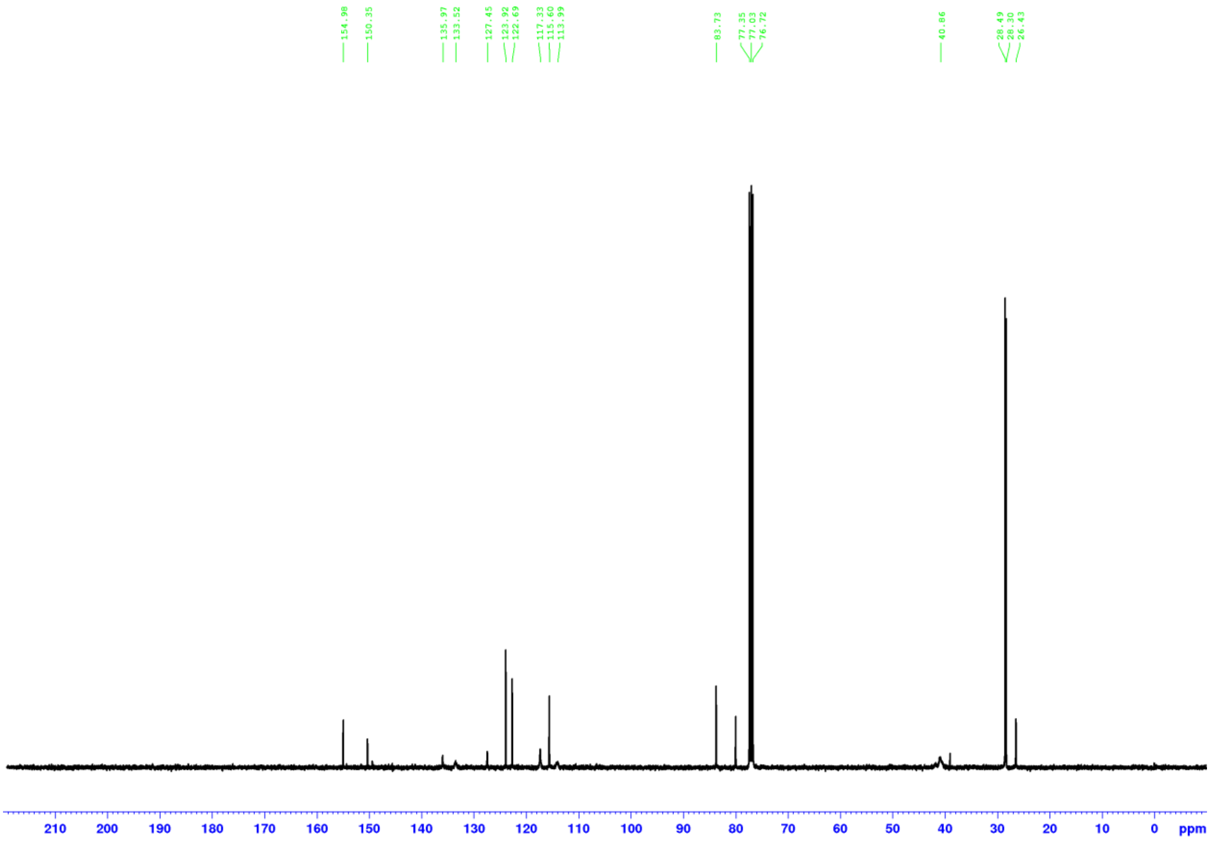

Figure S9. ^{13}C {1H} NMR ($CDCl_3$, 75 MHz) spectrum of bis-Boc compound **9f**

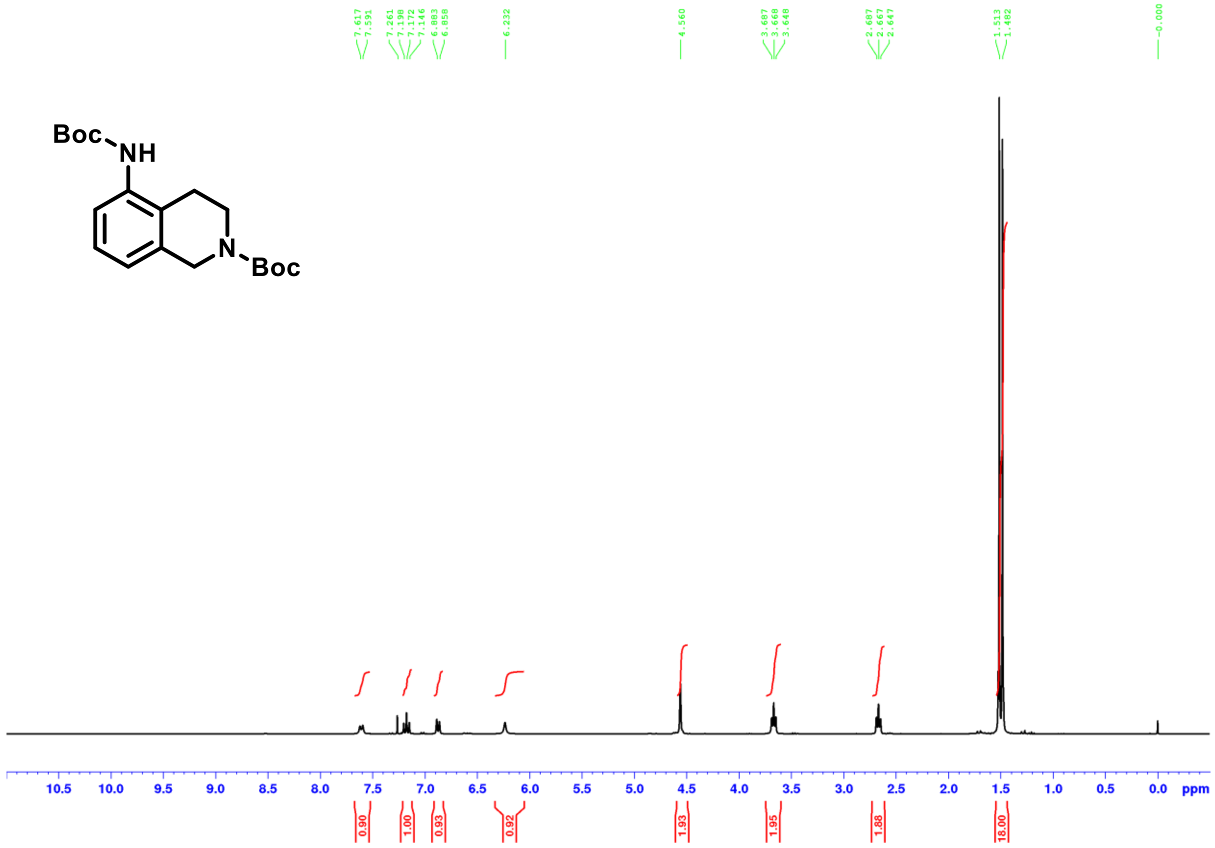

Figure S10. ^{1}H NMR ($CDCl_3$, 300 MHz) spectrum of bis-Boc compound **9g**

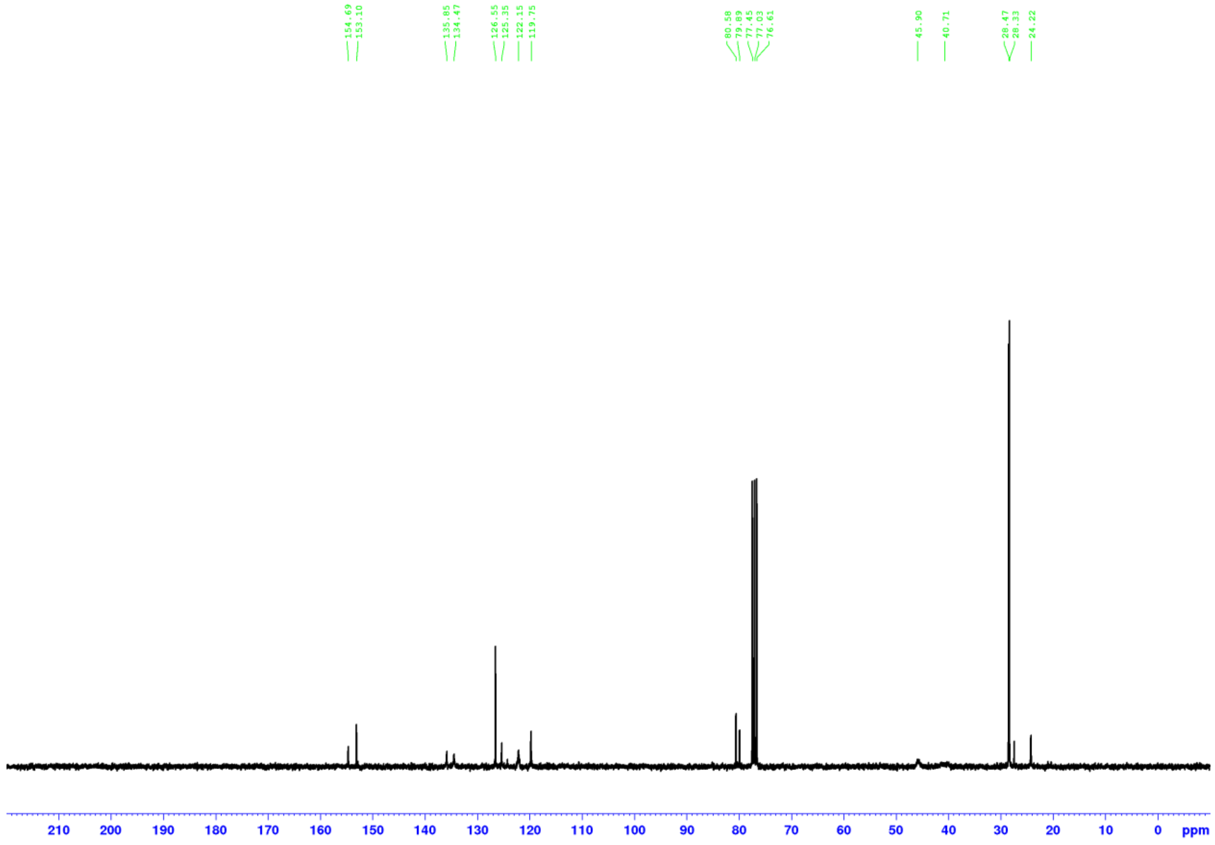

Figure S11. ^{13}C {1H} NMR ($CDCl_3$, 75 MHz) spectrum of bis-Boc compound **9g**

6. Copies of ^{1}HNMR and ^{13}C NMR Spectra

NMR spectra of the following compounds were in agreement with those previously reported.

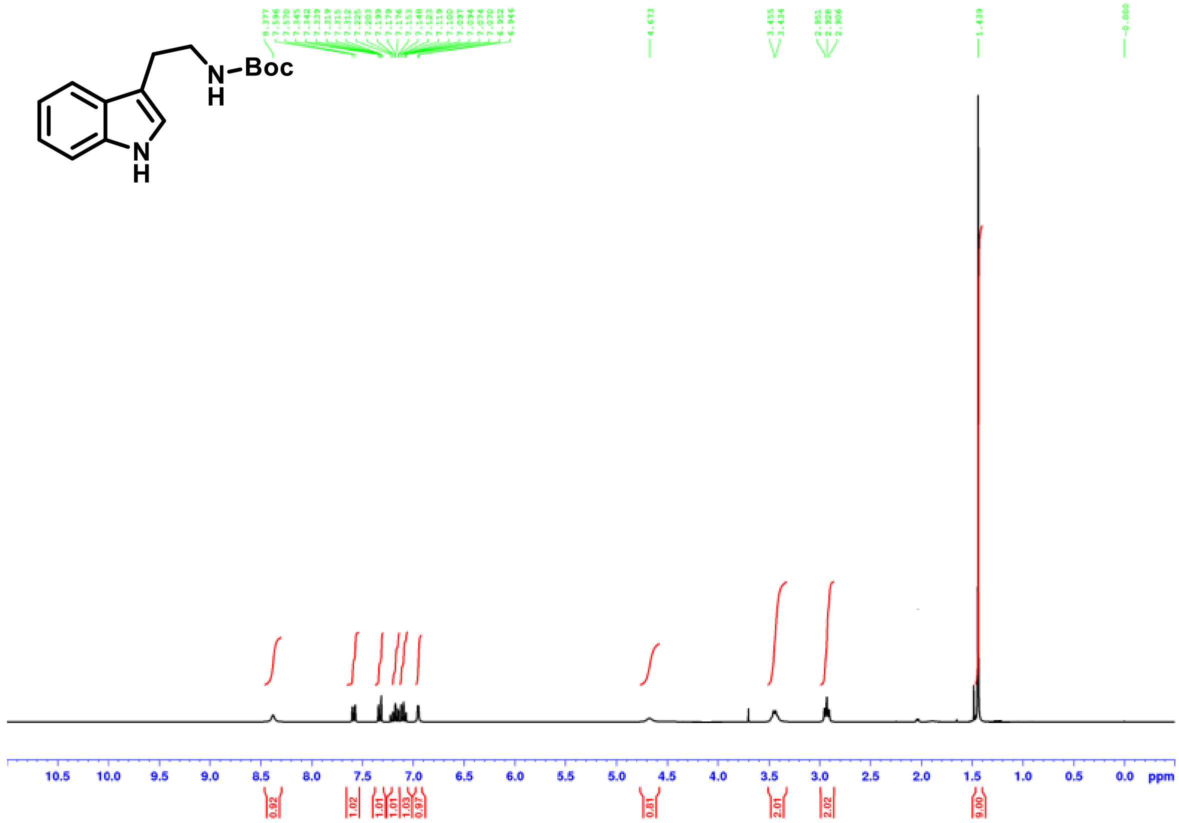

Figure S12. ^{1}H NMR ($CDCl_3$, 300 MHz) spectrum of mono-*N*-Boc compound **10a**

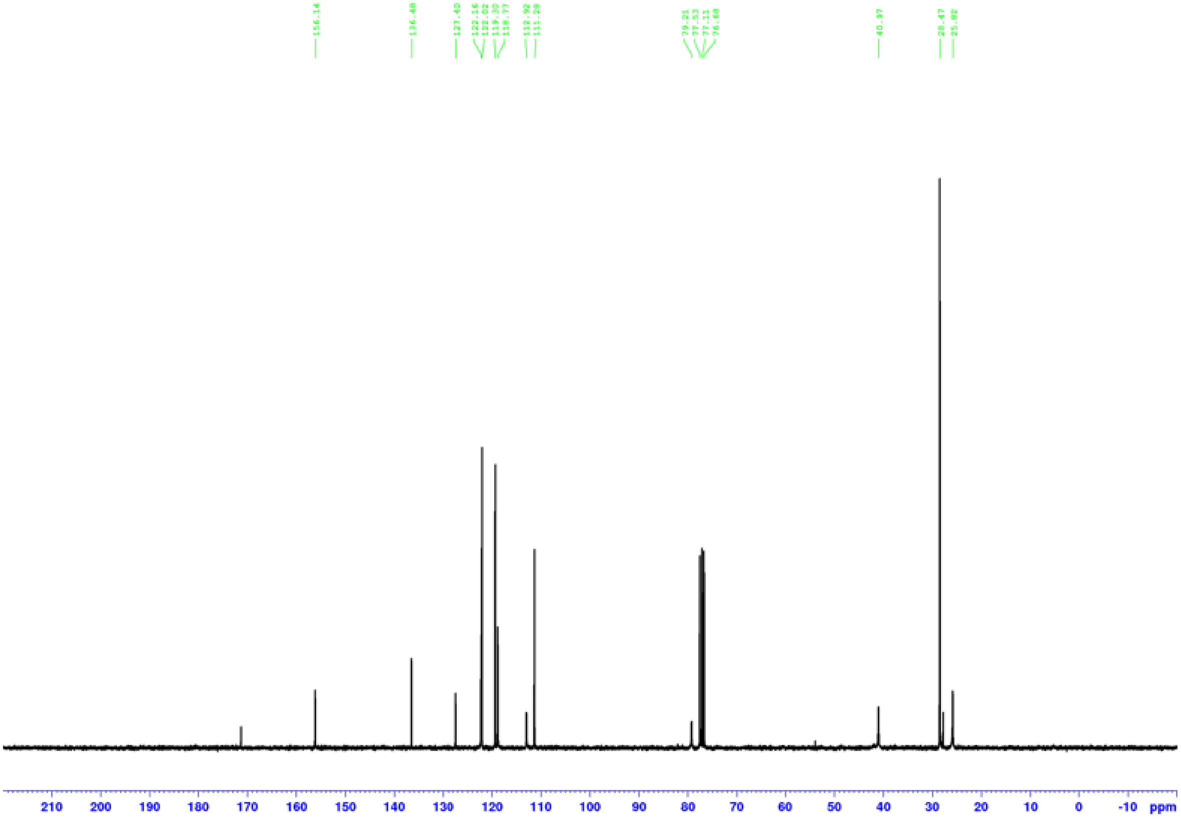

Figure S13. ^{13}C {1H} NMR ($CDCl_3$, 75 MHz) spectrum of mono-*N*-Boc compound **10a**

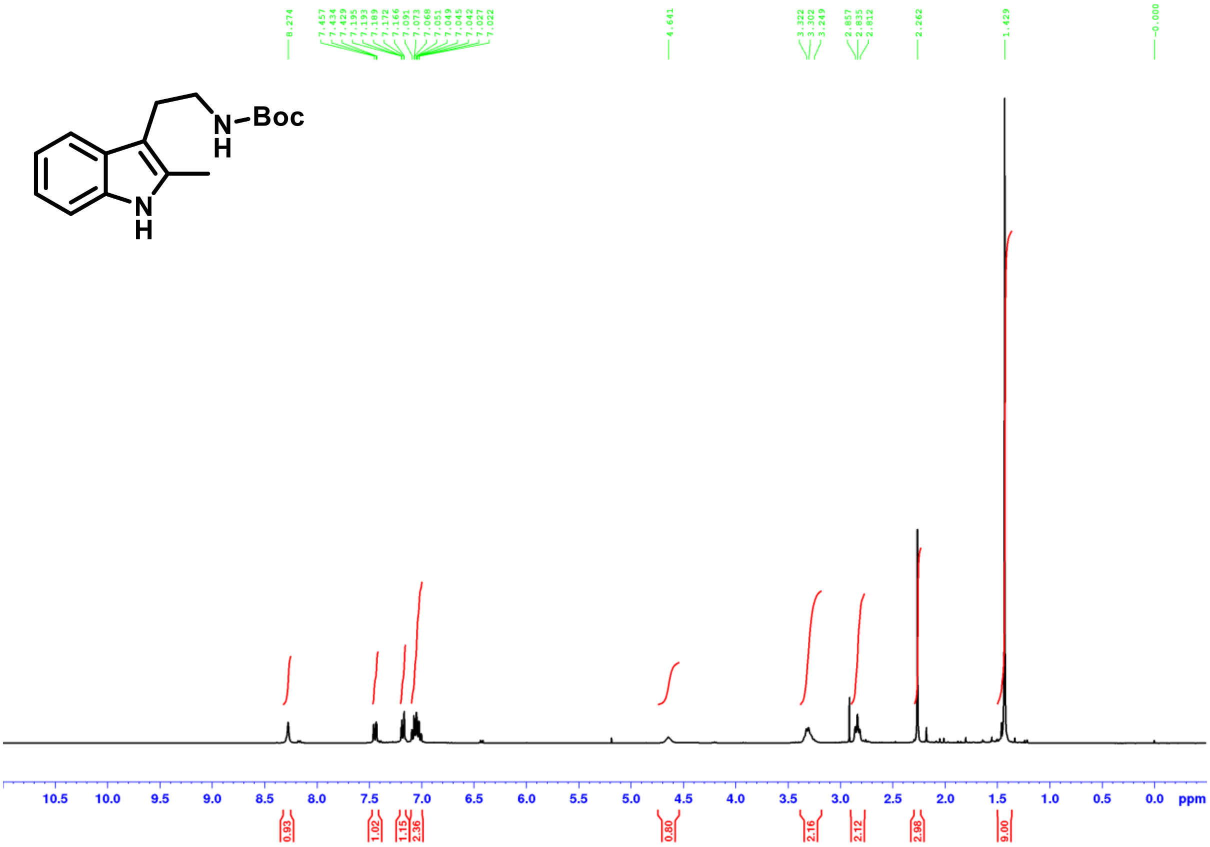

Figure S14. ^{1}H NMR ($CDCl_3$, 300 MHz) spectrum of mono-*N*-Boc compound **10b**

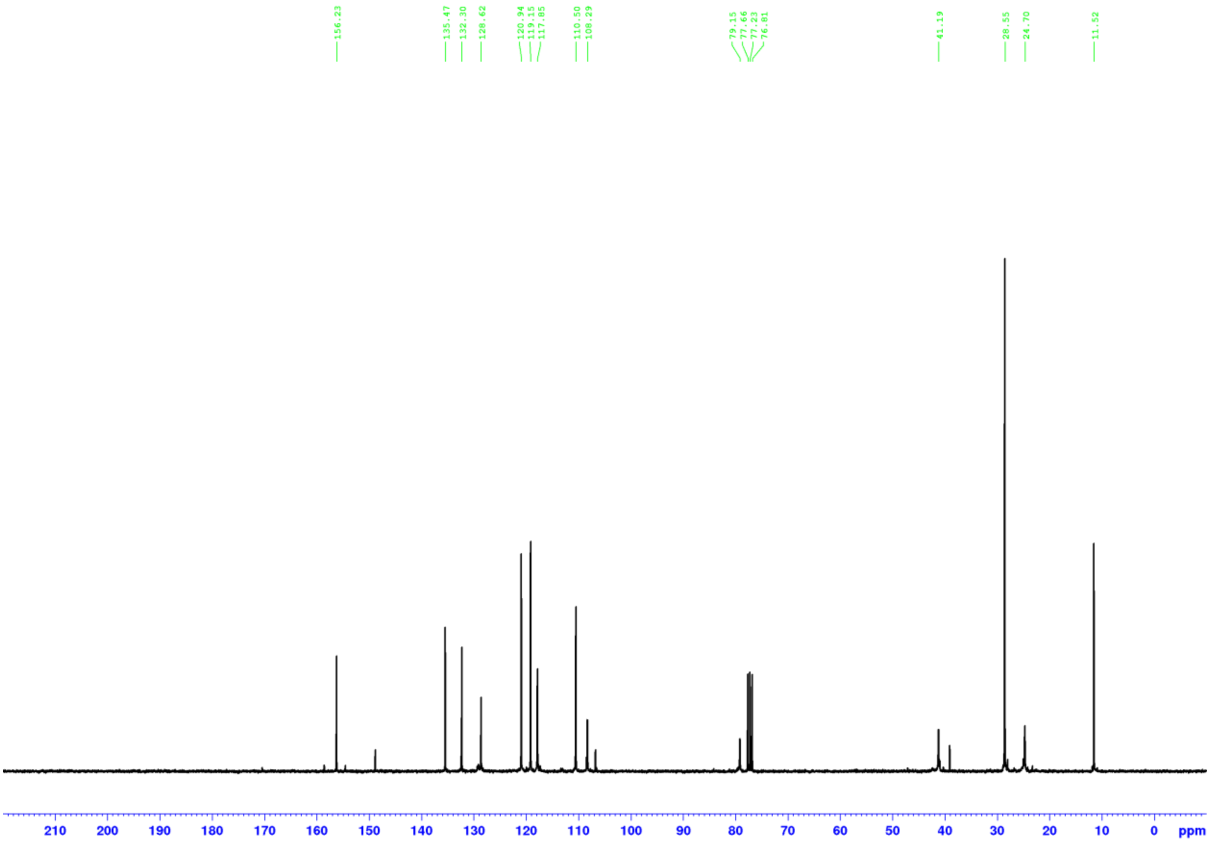

Figure S15. ^{13}C {1H} NMR ($CDCl_3$, 75 MHz) spectrum of mono-*N*-Boc compound **10b**

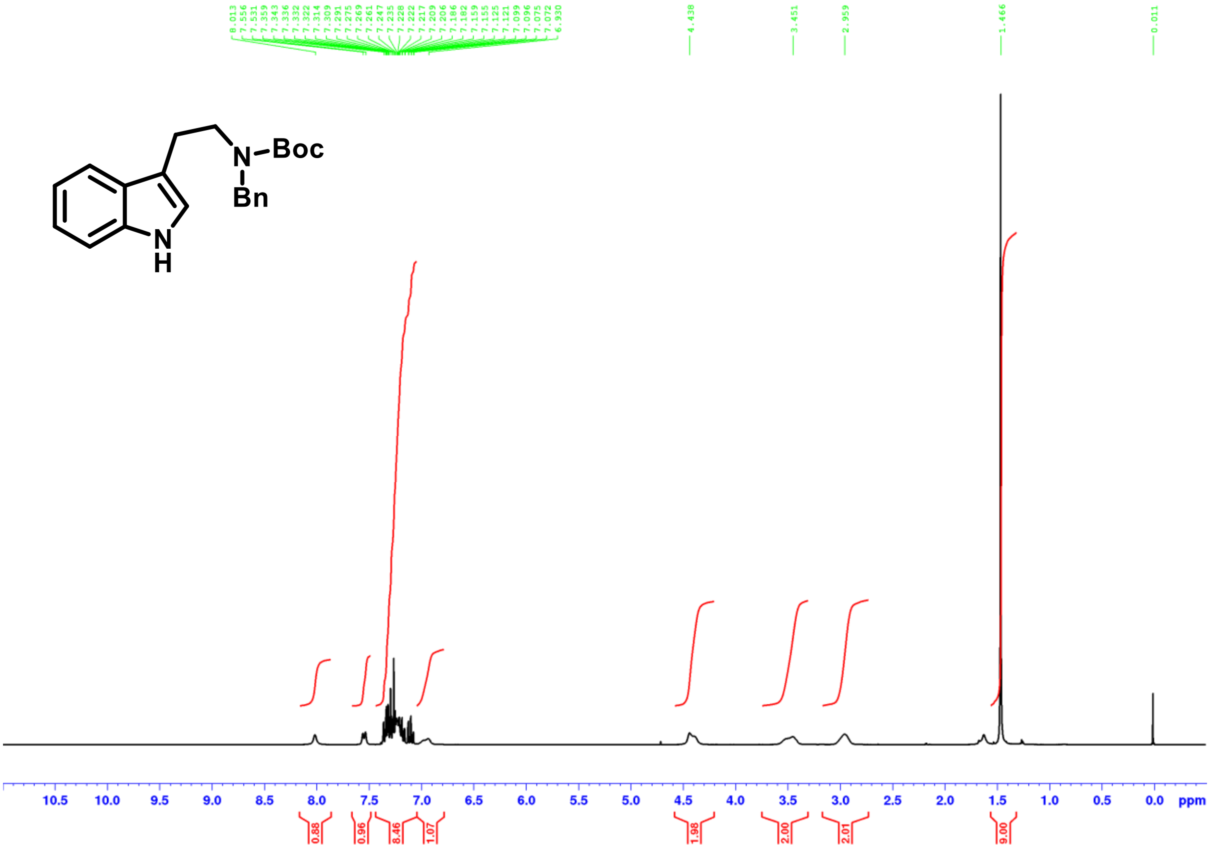

Figure S16. ^{1}H NMR ($CDCl_3$, 300 MHz) spectrum of mono-*N*-Boc compound **10c**

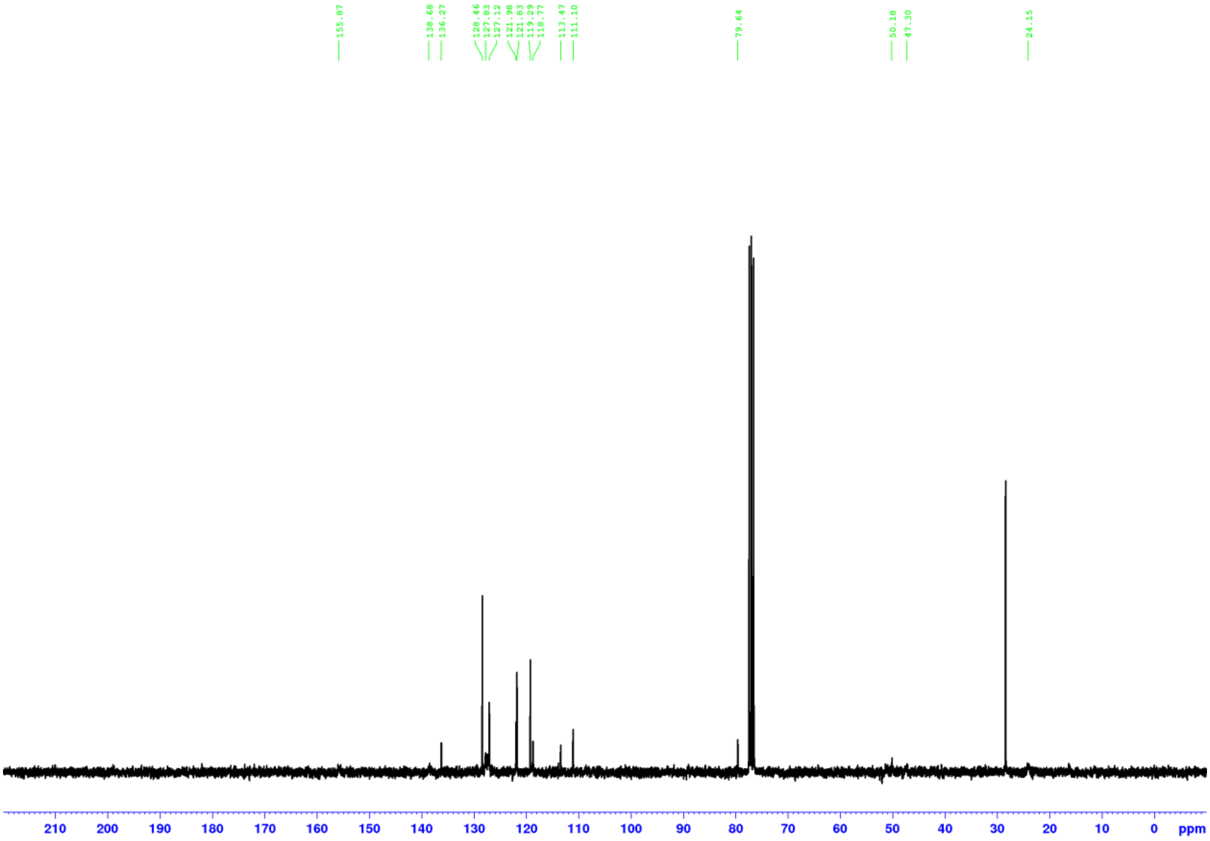

Figure S17. ^{13}C {1H} NMR ($CDCl_3$, 75 MHz) spectrum of mono-*N*-Boc compound **10c**

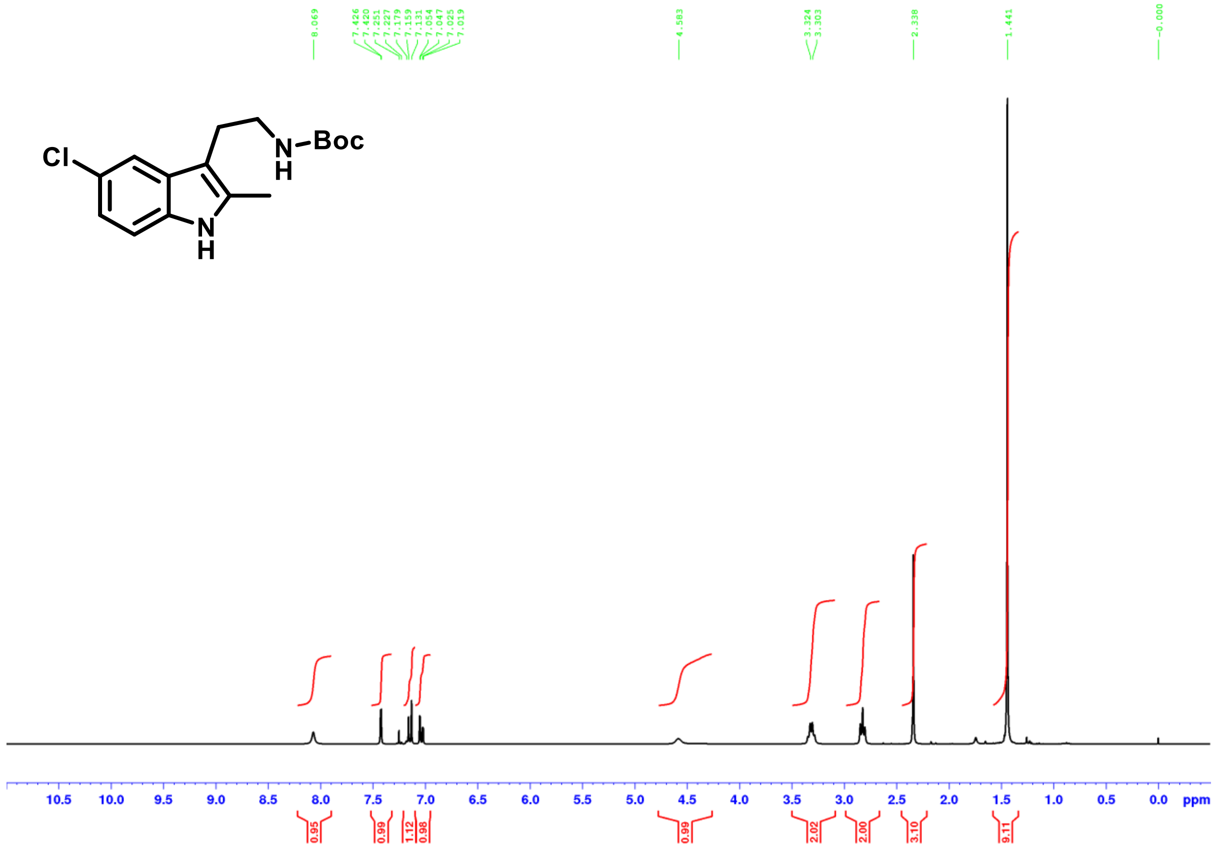

Figure S18. ^{1}H NMR ($CDCl_3$, 300 MHz) spectrum of mono-*N*-Boc compound **10d**

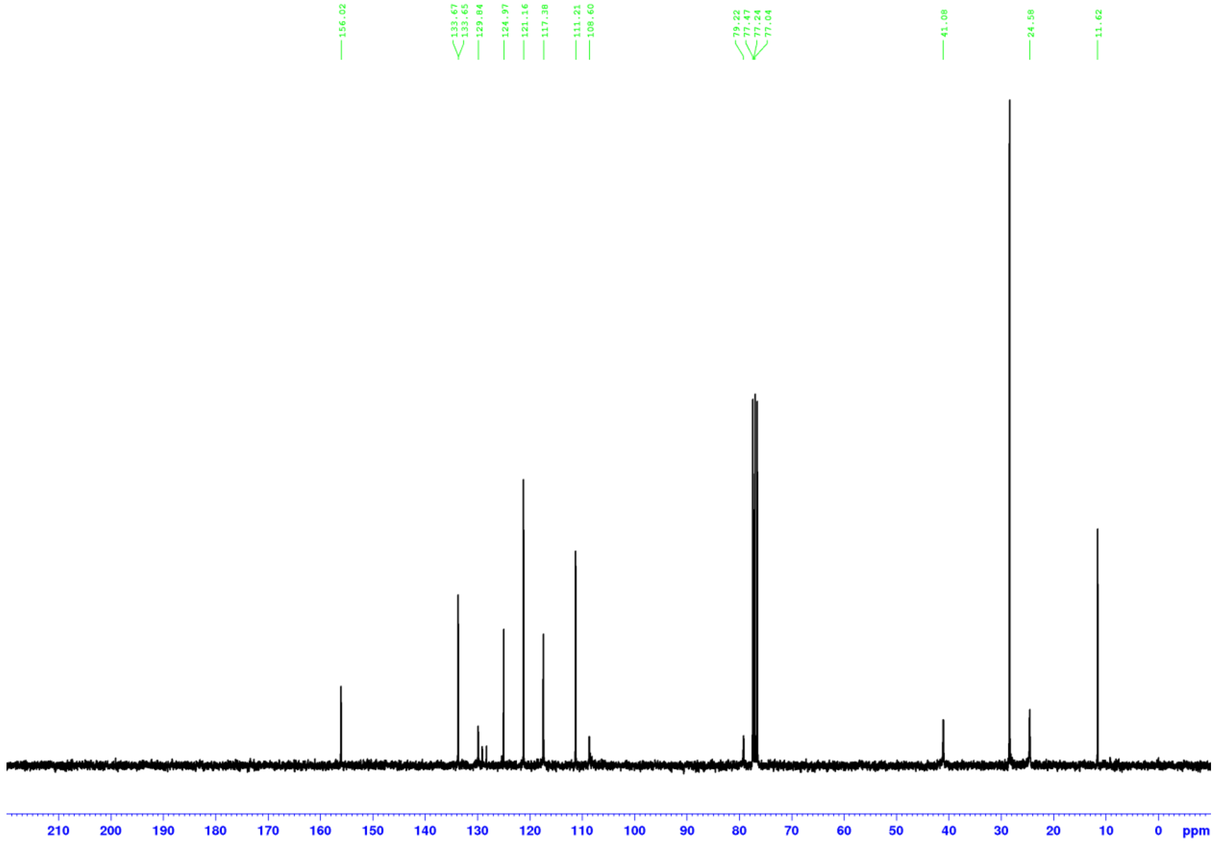

Figure S19. ^{13}C {1H} NMR ($CDCl_3$, 75 MHz) spectrum of mono-*N*-Boc compound **10d**

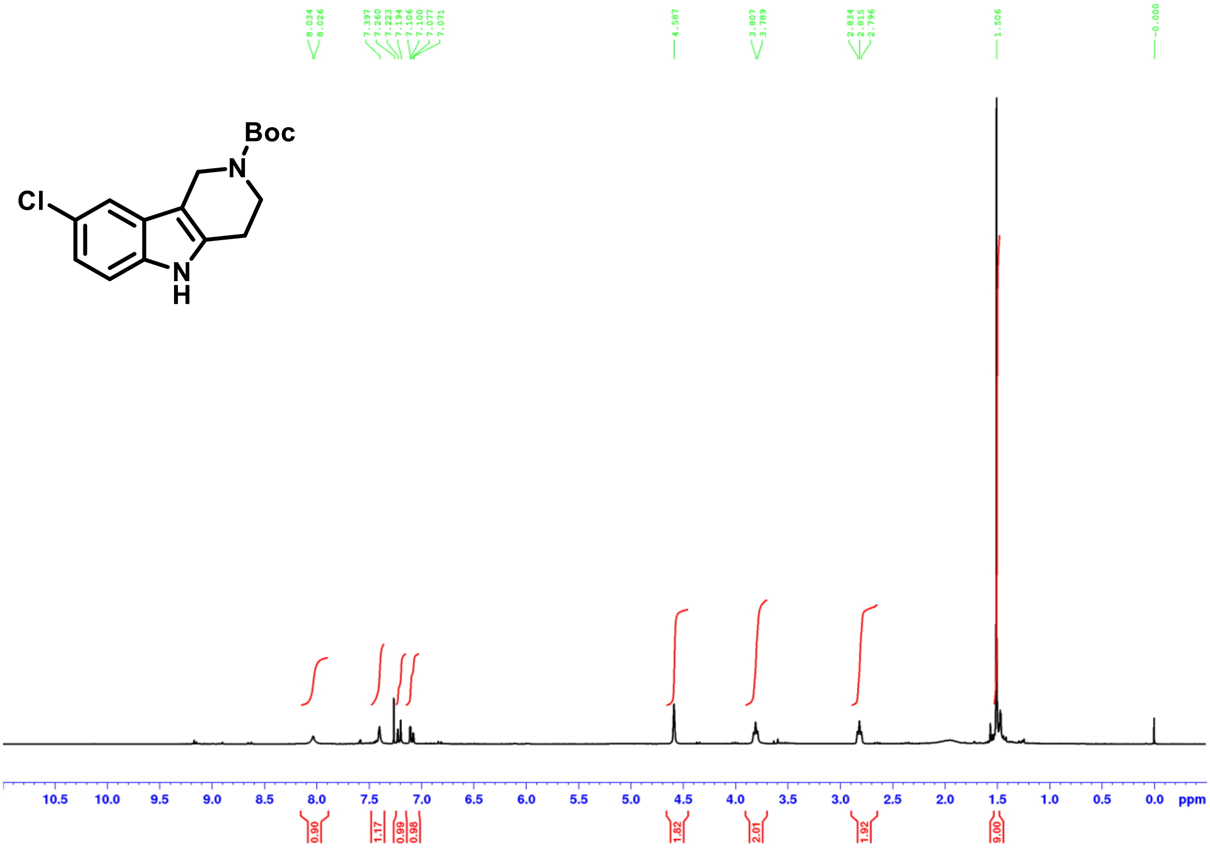

Figure S20. ^{1}H NMR ($CDCl_3$, 300 MHz) spectrum of mono-*N*-Boc compound **10e**

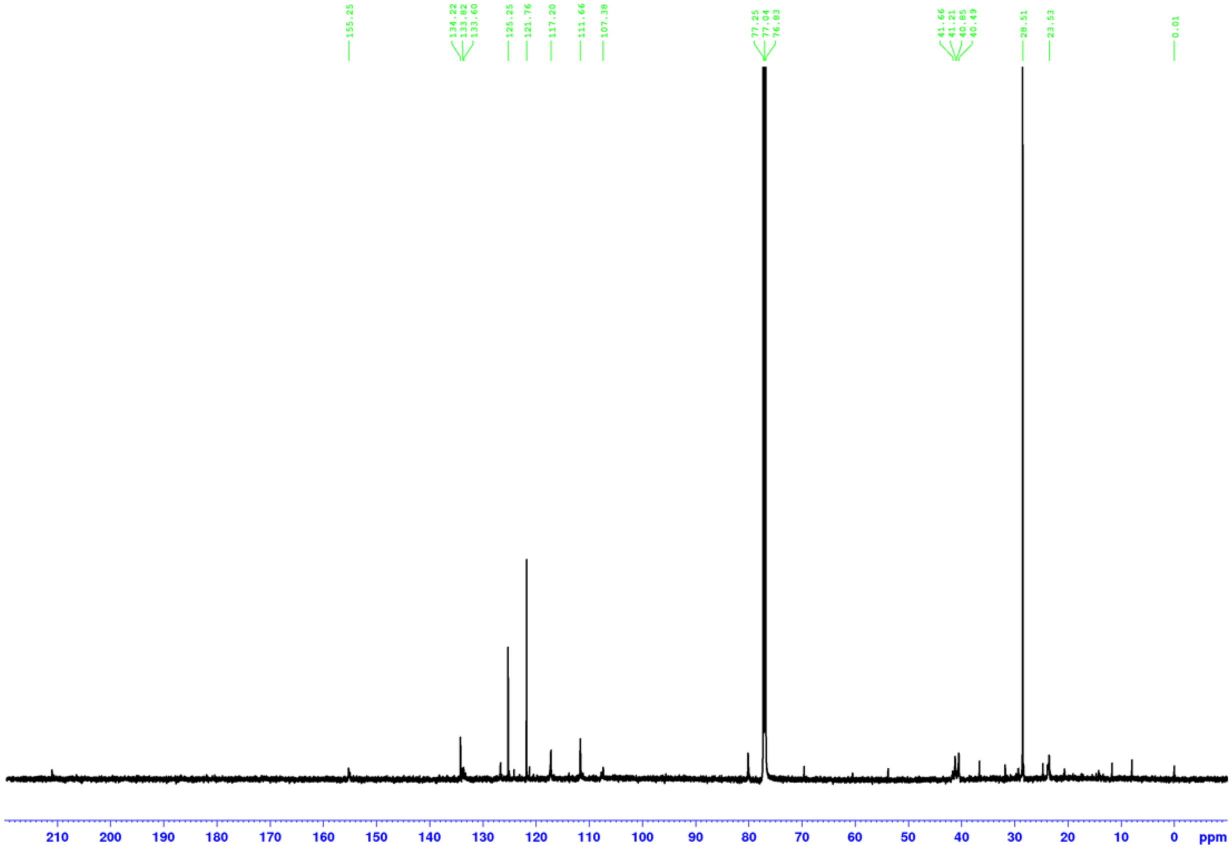

Figure S21. ^{13}C {1H} NMR ($CDCl_3$, 75 MHz) spectrum of mono-*N*-Boc compound **10e**

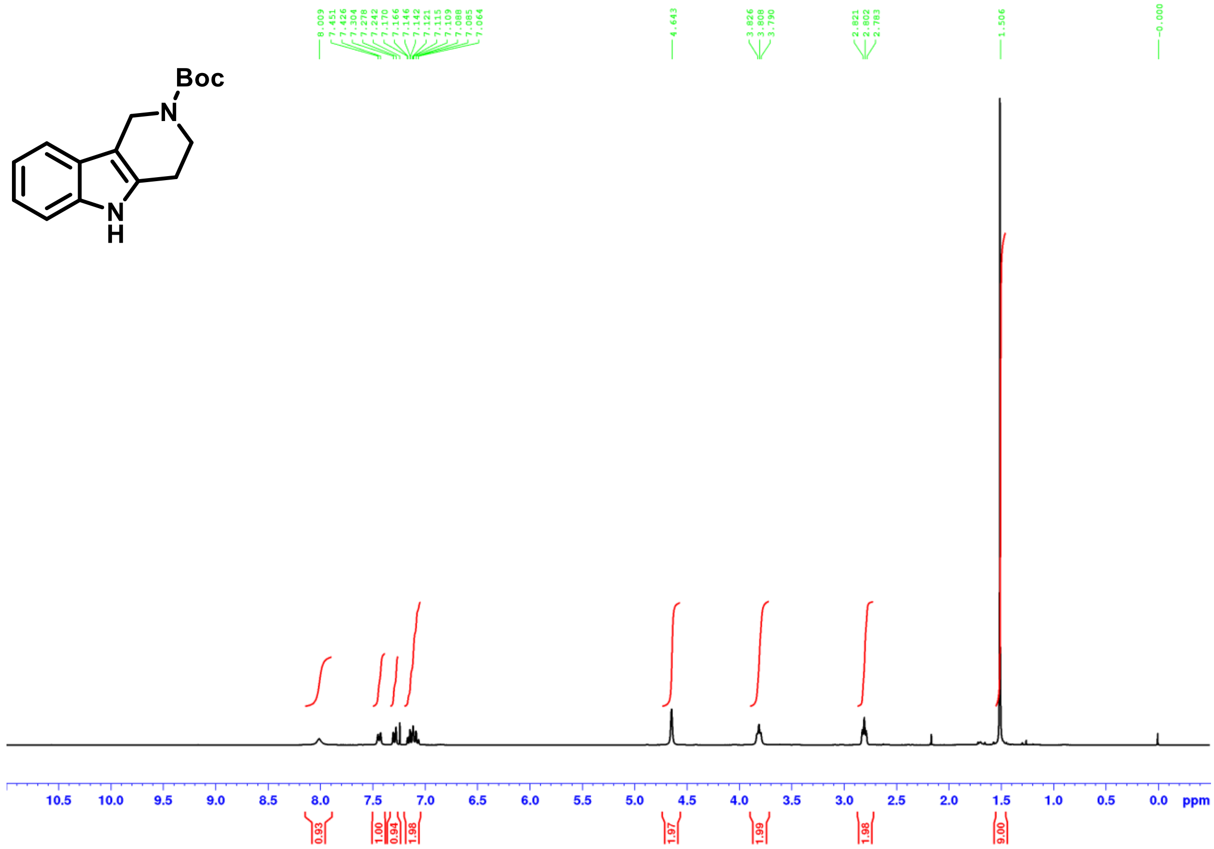

Figure S22. ^{1}H NMR ($CDCl_3$, 300 MHz) spectrum of mono-*N*-Boc compound **10f**

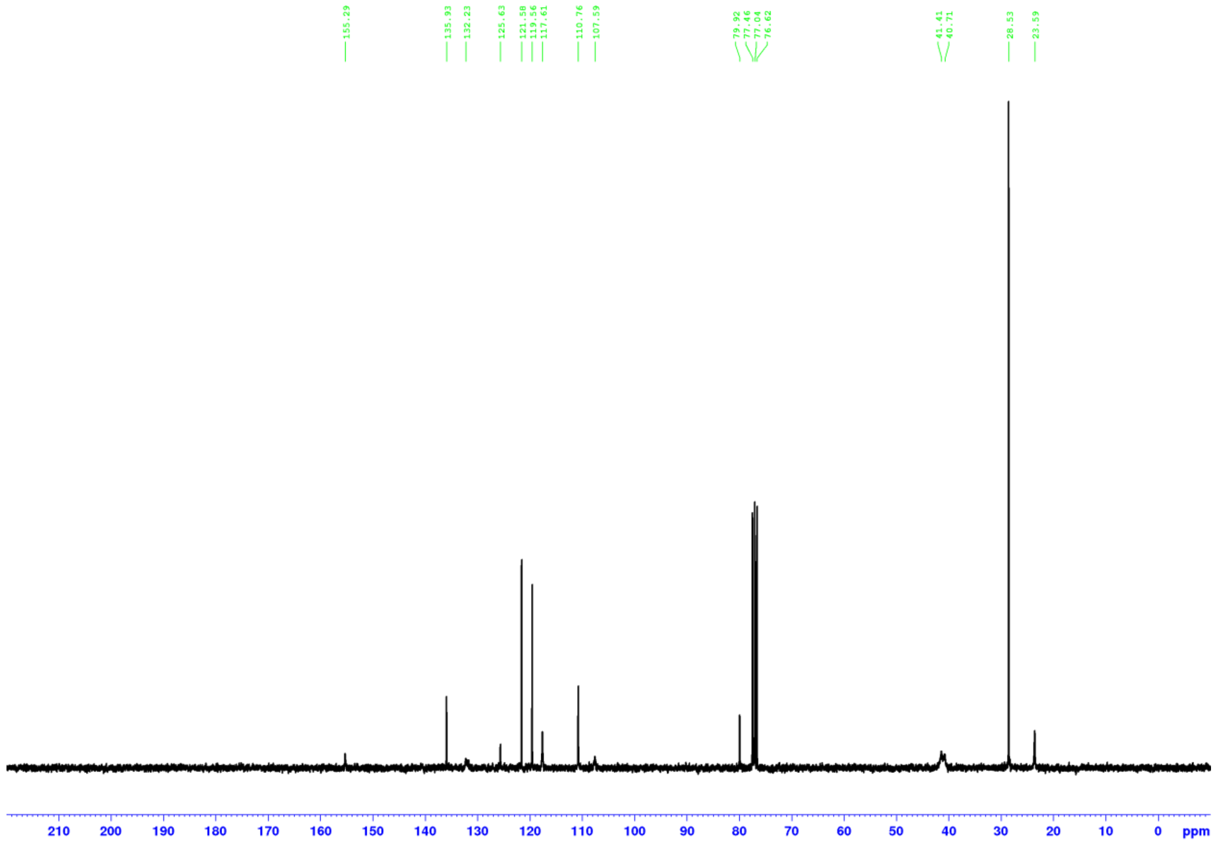

Figure S23. ^{13}C {1H} NMR ($CDCl_3$, 75 MHz) spectrum of mono-*N*-Boc compound **10f**

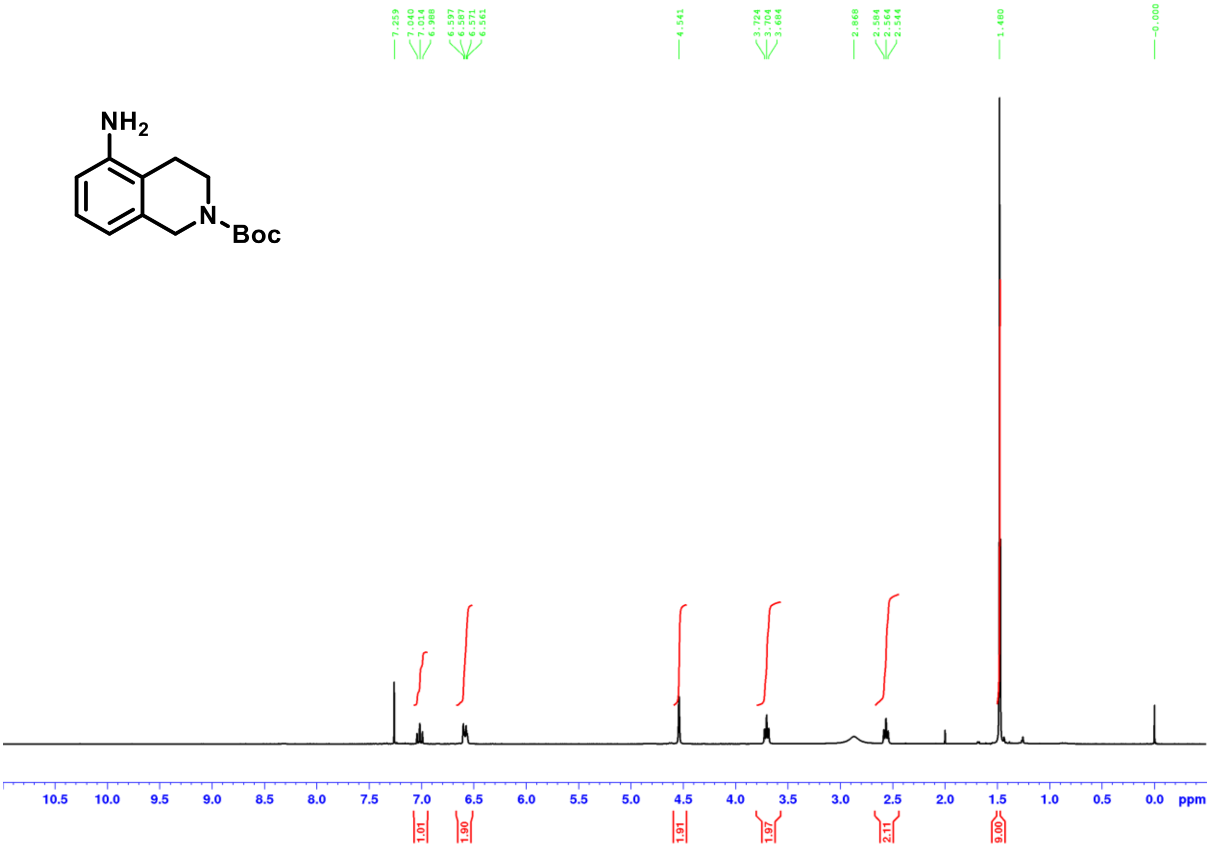

Figure S24. ^{1}H NMR ($CDCl_3$, 300 MHz) spectrum of mono-*N*-Boc compound **10g**

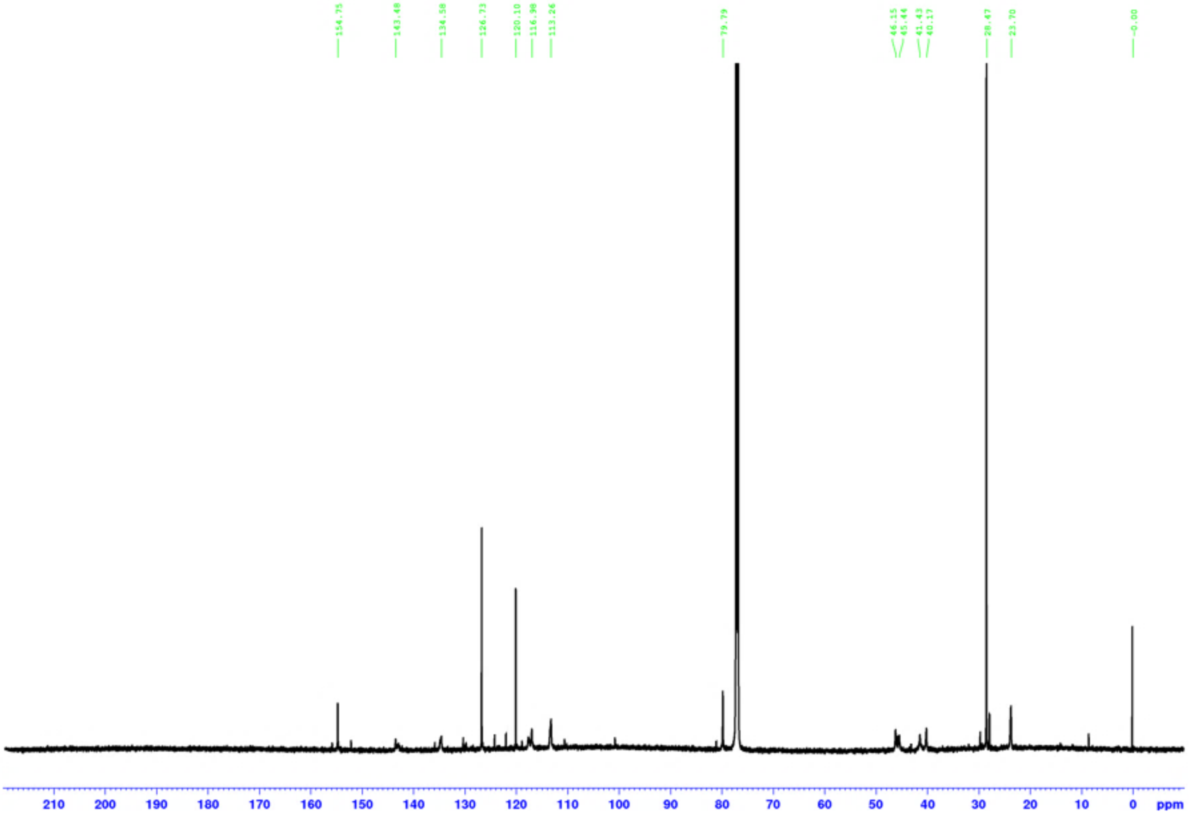

Figure S25. ^{13}C {1H} NMR ($CDCl_3$, 75 MHz) spectrum of mono-*N*-Boc compound **10g**

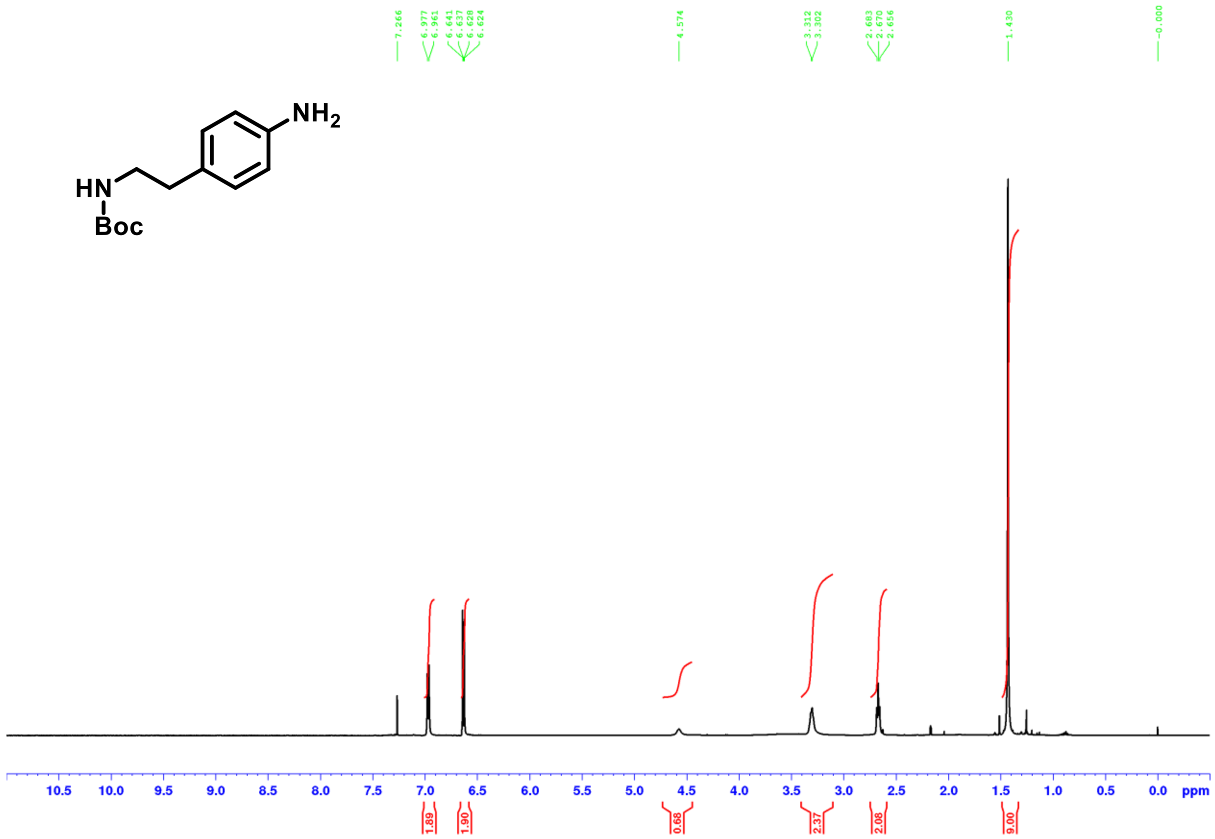

Figure S26. ^{1}H NMR ($CDCl_3$, 500 MHz) spectrum of mono-Boc amine **10h**

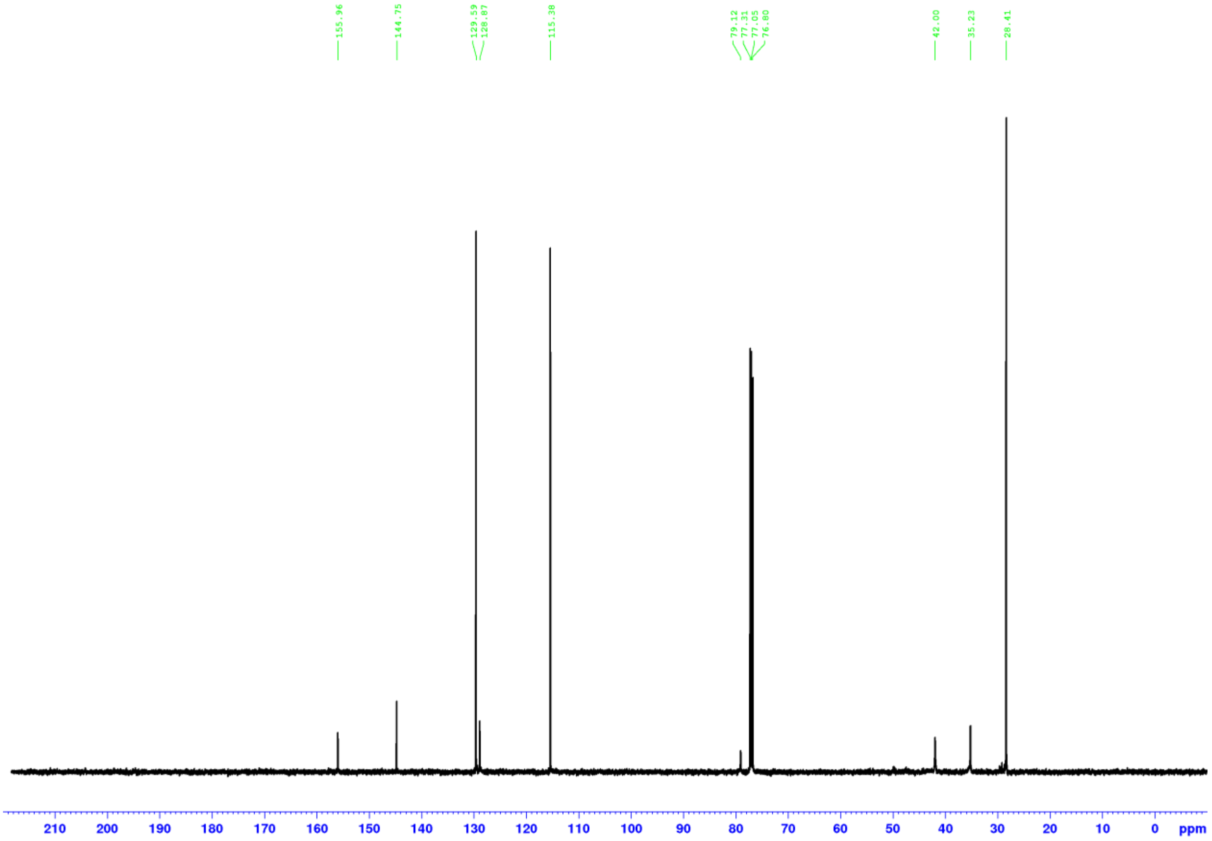

Figure S27. ^{13}C {1H} NMR ($CDCl_3$, 125 MHz) spectrum of mono-*N*-Boc compound **10h**

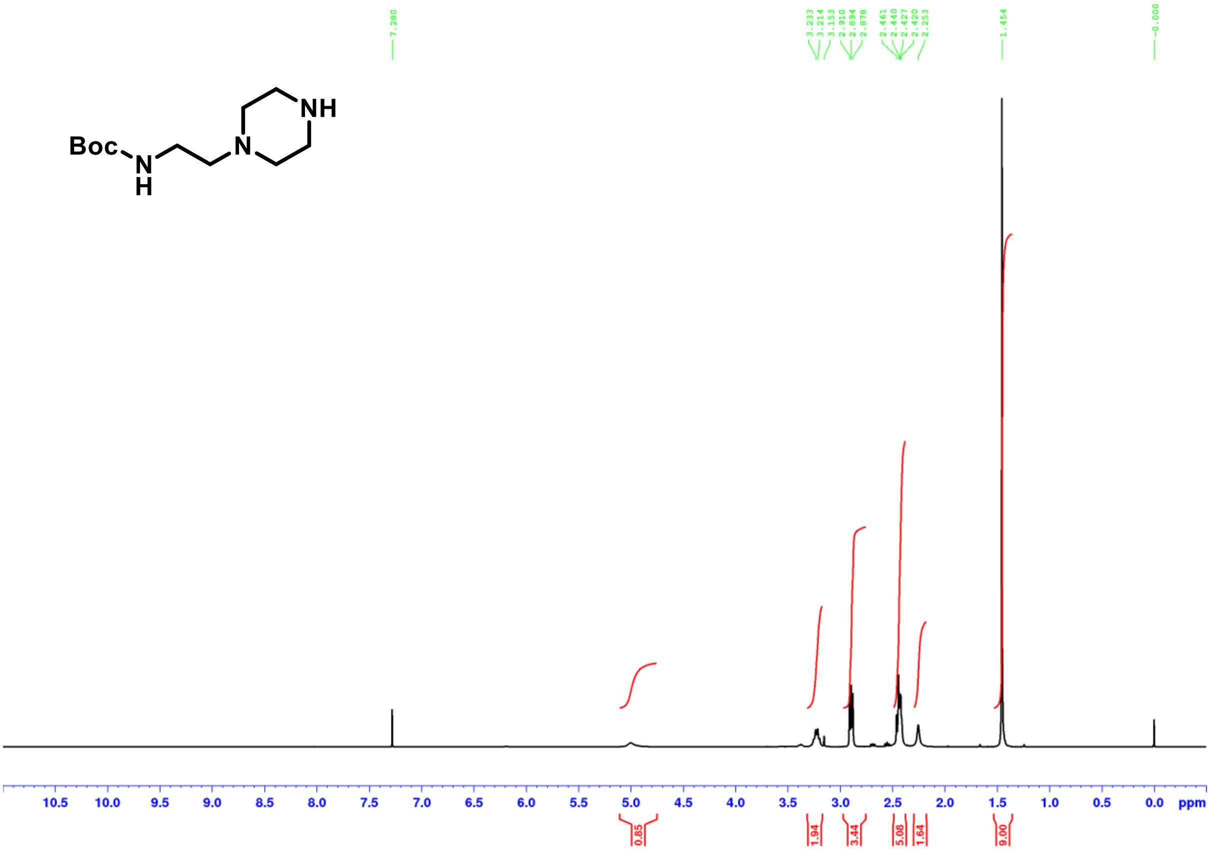

Figure S28. ^{1}H NMR ($CDCl_3$, 300 MHz) spectrum of mono-Boc amine **10i**

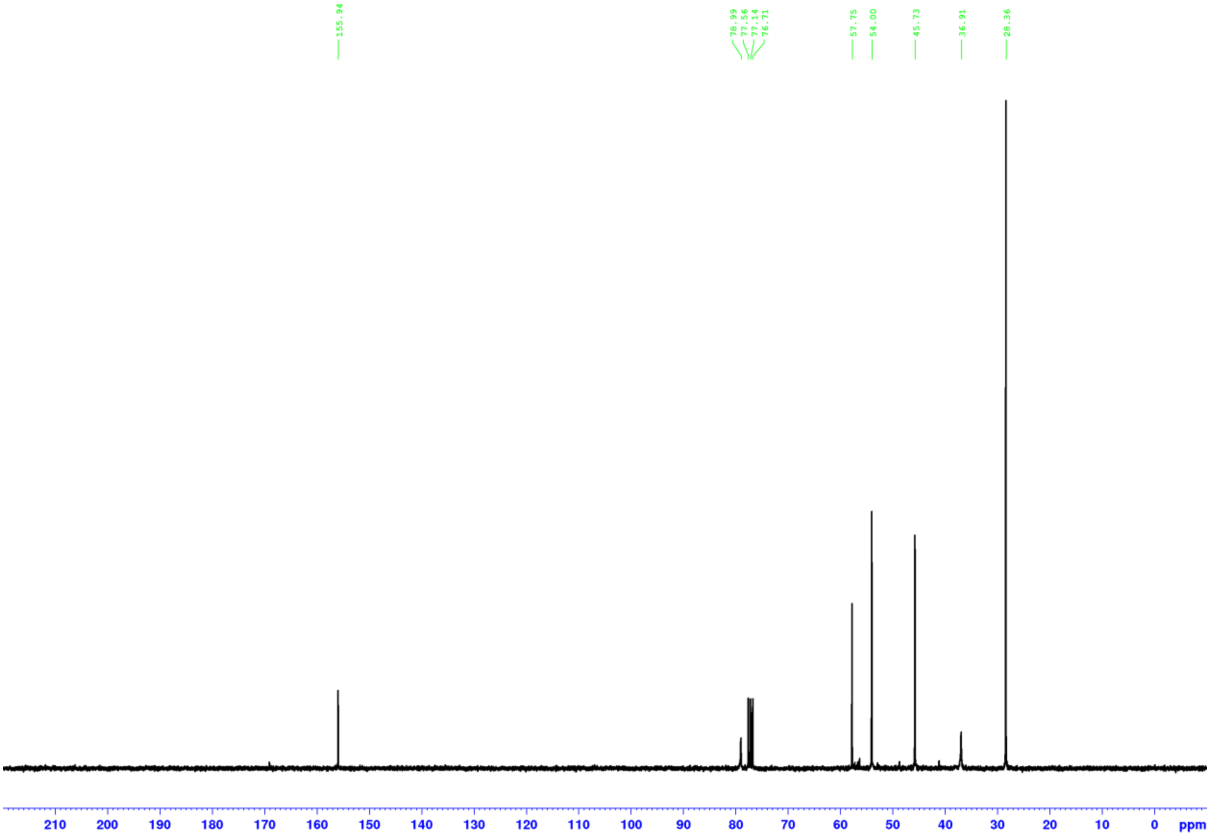

Figure S29. ^{13}C {1H} NMR ($CDCl_3$, 75 MHz) spectrum of mono-*N*-Boc compound **10i**

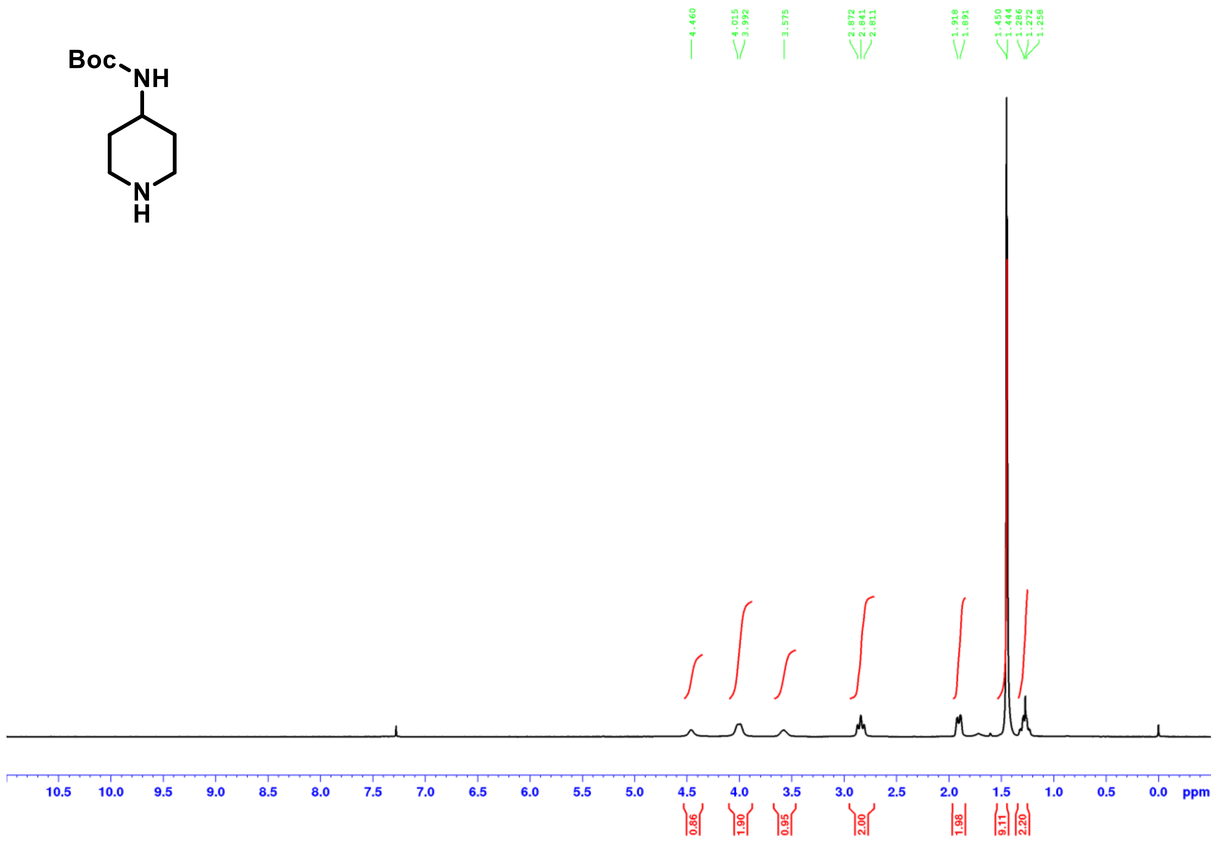

Figure S30. ^{1}H NMR ($CDCl_3$, 400 MHz) spectrum of mono-Boc amine **10j**

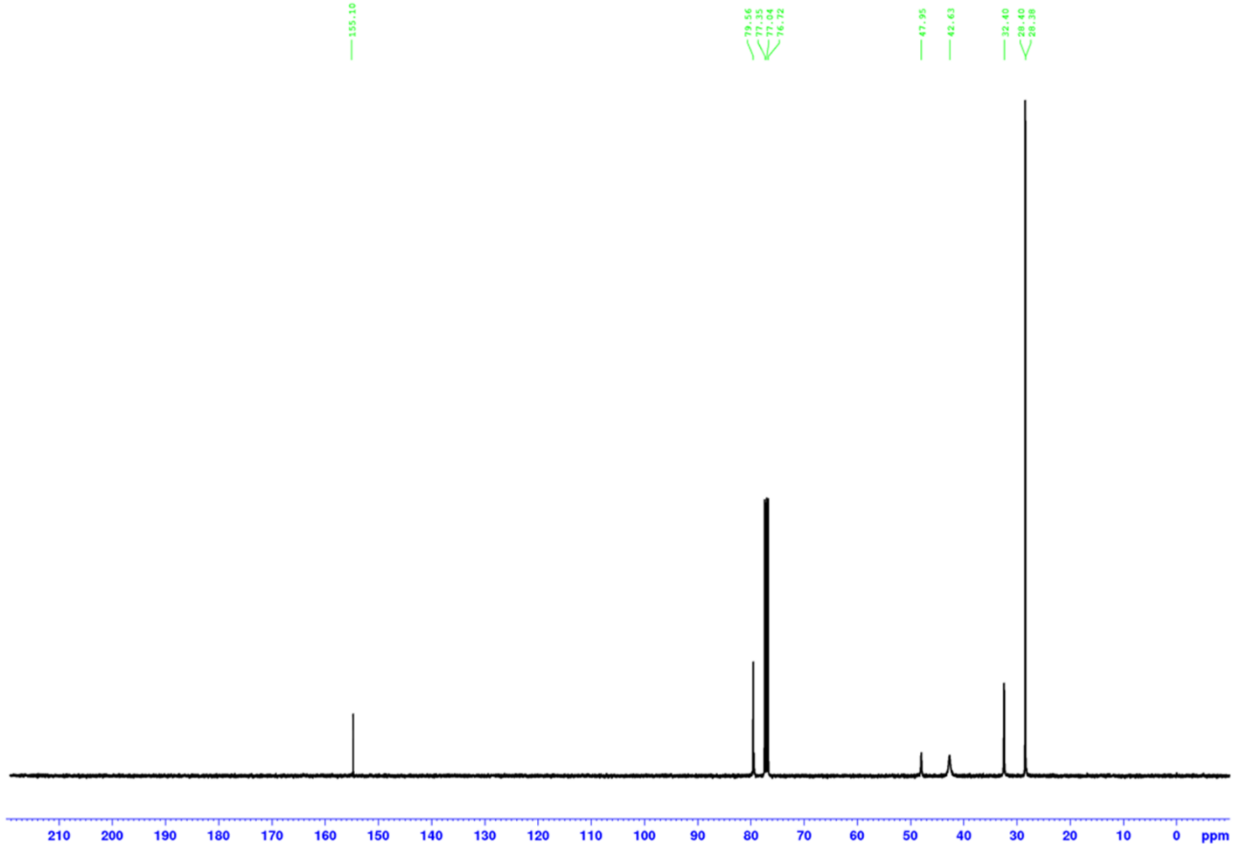

Figure S31. ^{13}C {1H} NMR ($CDCl_3$, 100 MHz) spectrum of mono-*N*-Boc compound **10j**

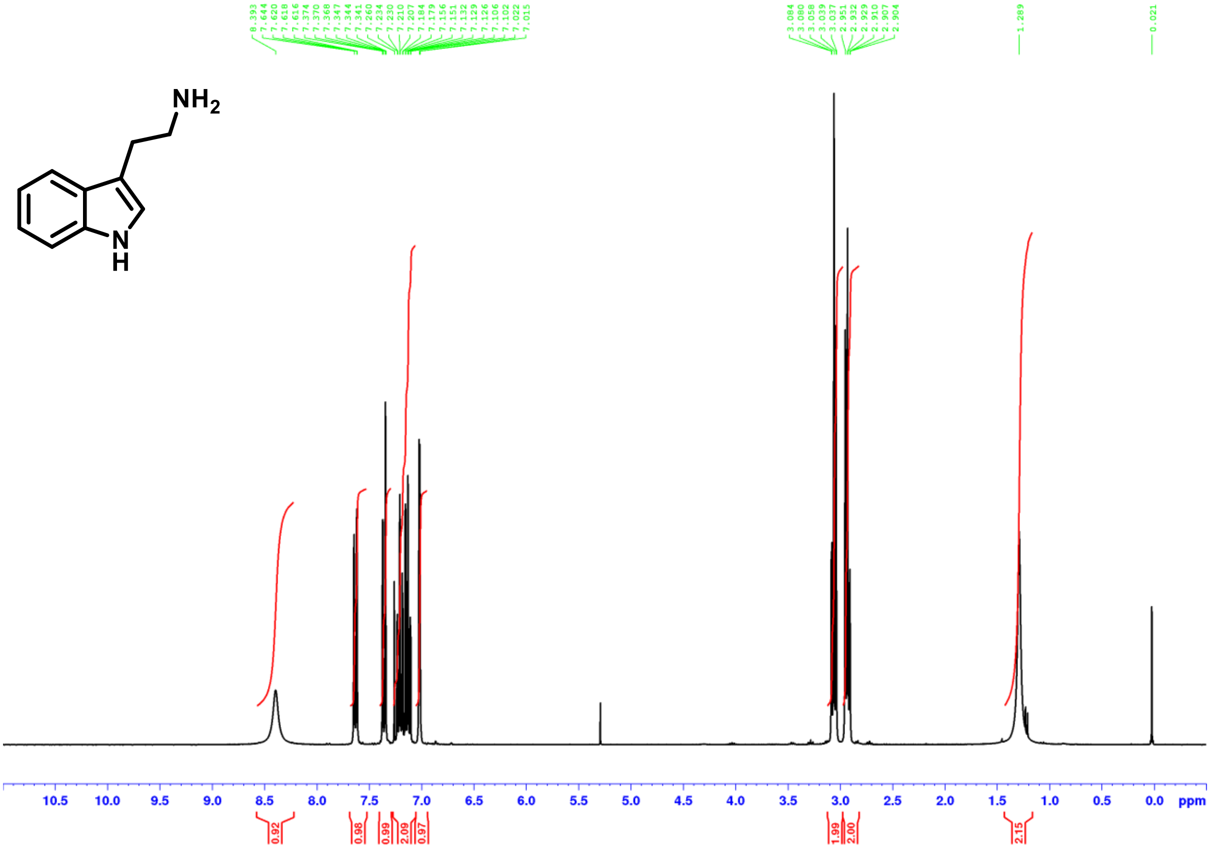

Figure S32. ^{1}H NMR ($CDCl_3$, 400 MHz) spectrum of diamine compound **11a**

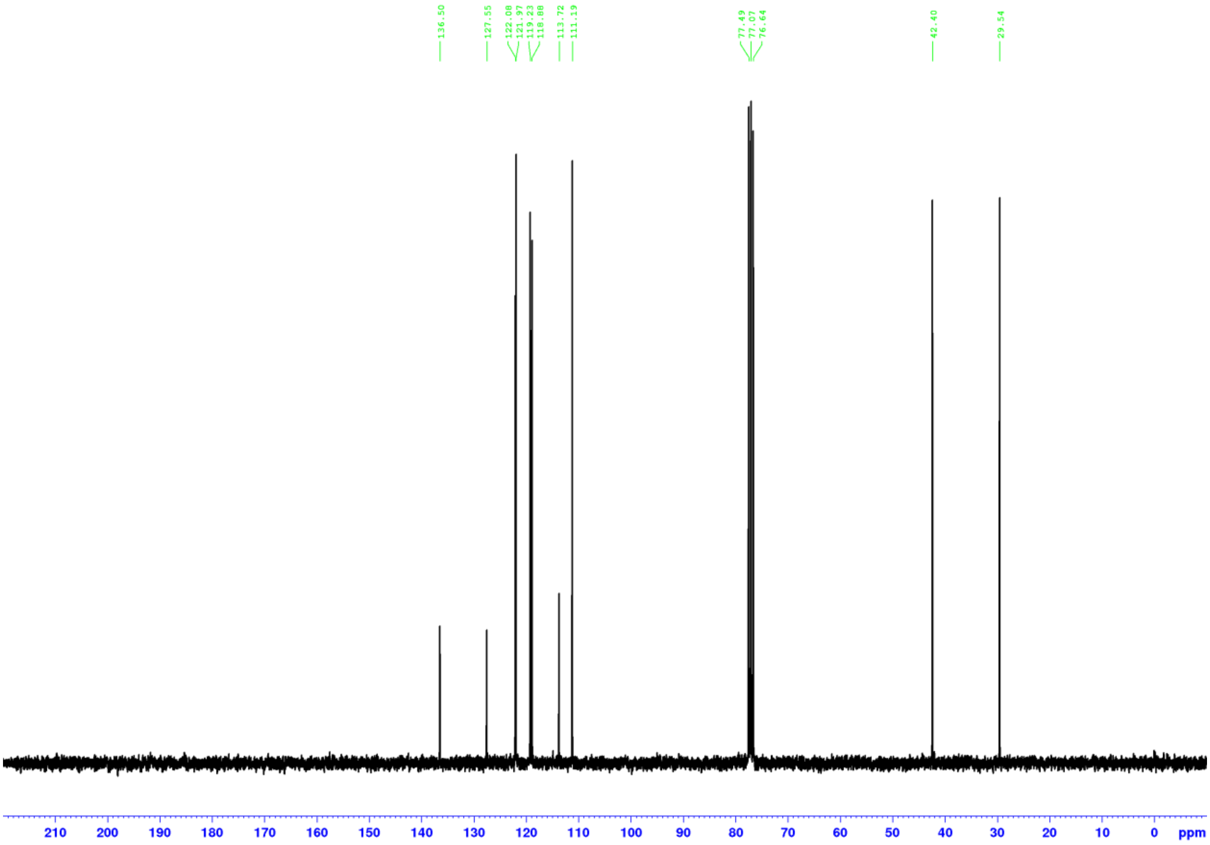

Figure S33. ^{13}C {1H} NMR ($CDCl_3$, 100 MHz) spectrum of diamine compound **11a**

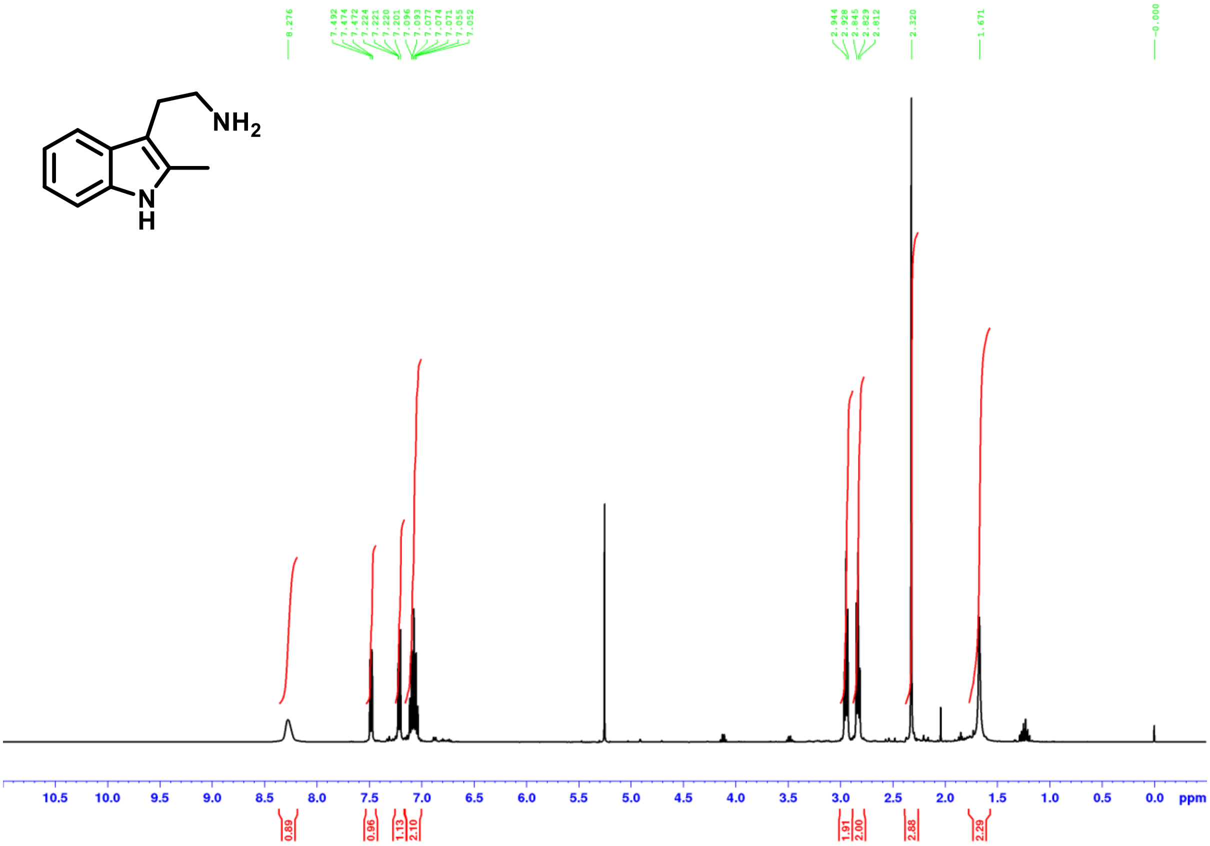

Figure S34. ^{1}H NMR ($CDCl_3$, 400 MHz) spectrum of diamine compound **11b**

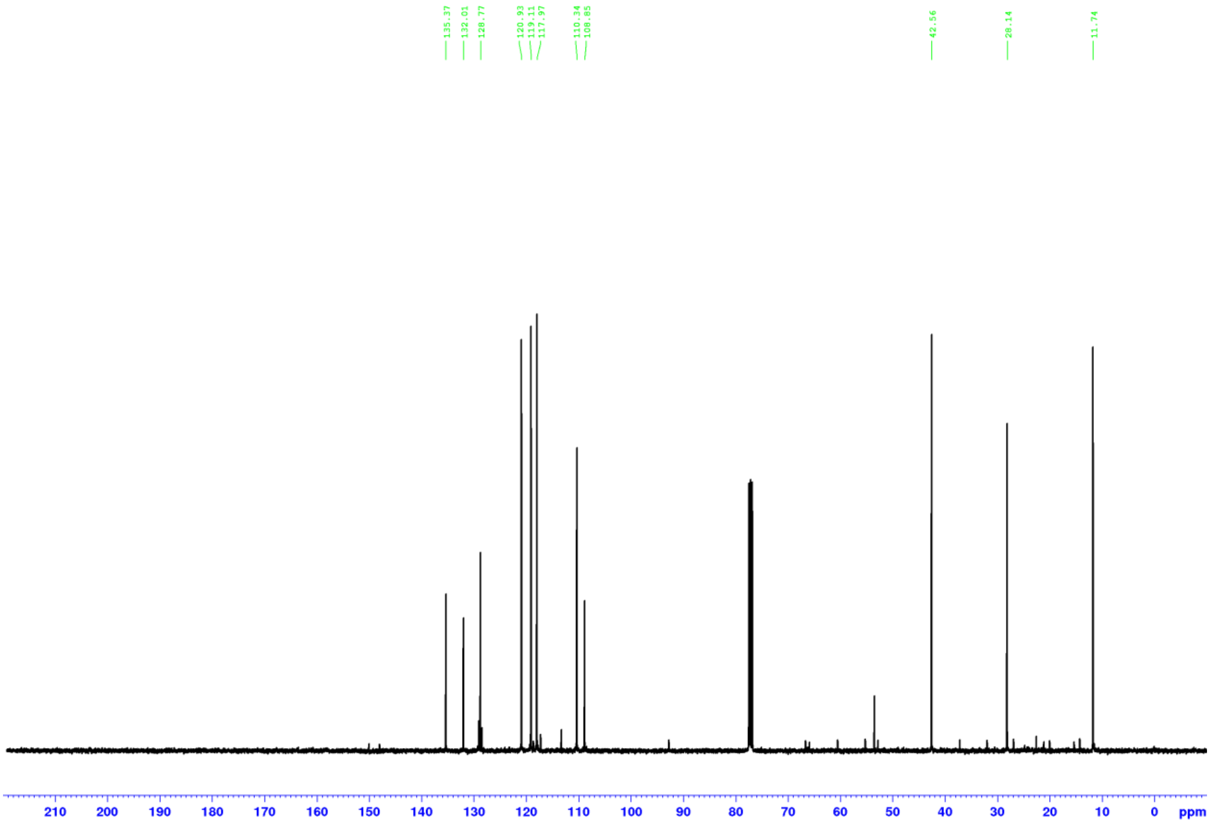

Figure S35. ^{13}C {1H} NMR ($CDCl_3$, 100 MHz) spectrum of diamine compound **11b**

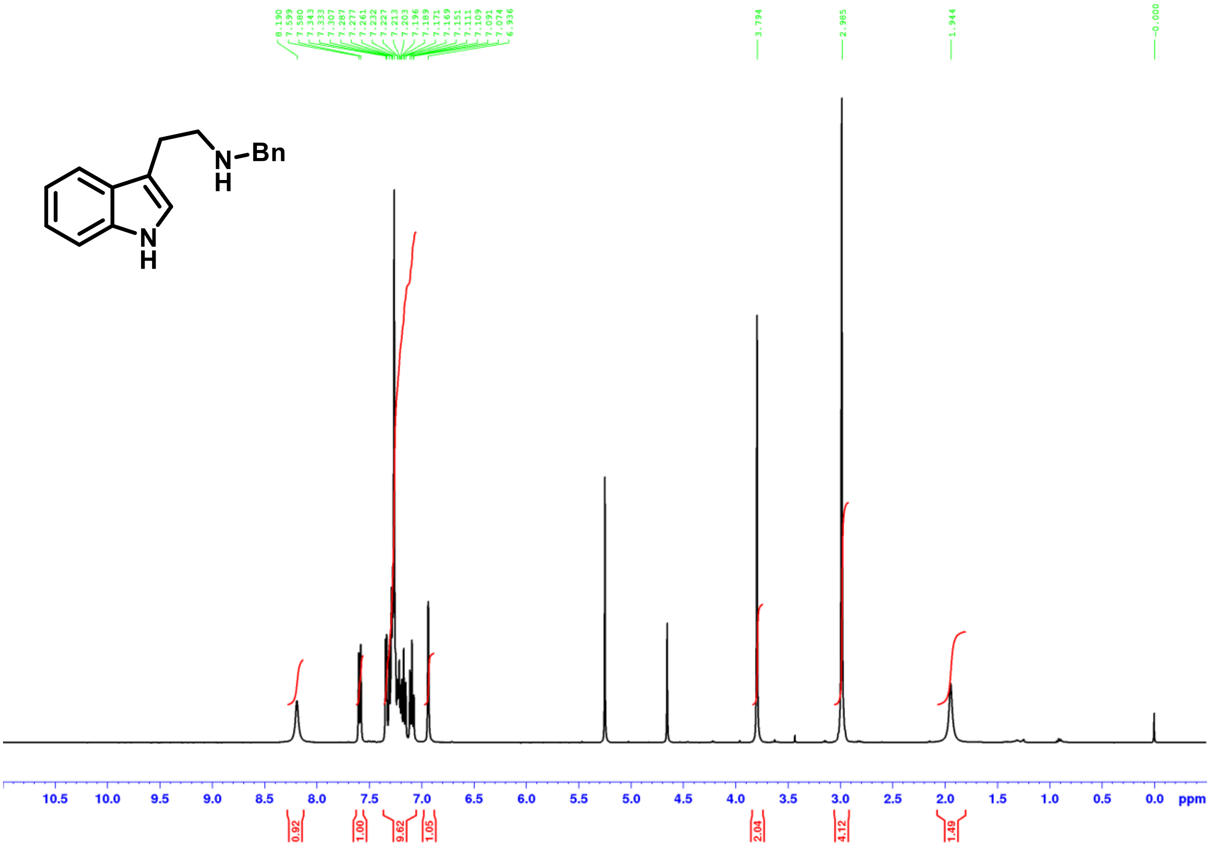

Figure S36. ^{1}H NMR ($CDCl_3$, 400 MHz) spectrum of diamine compound **11c**

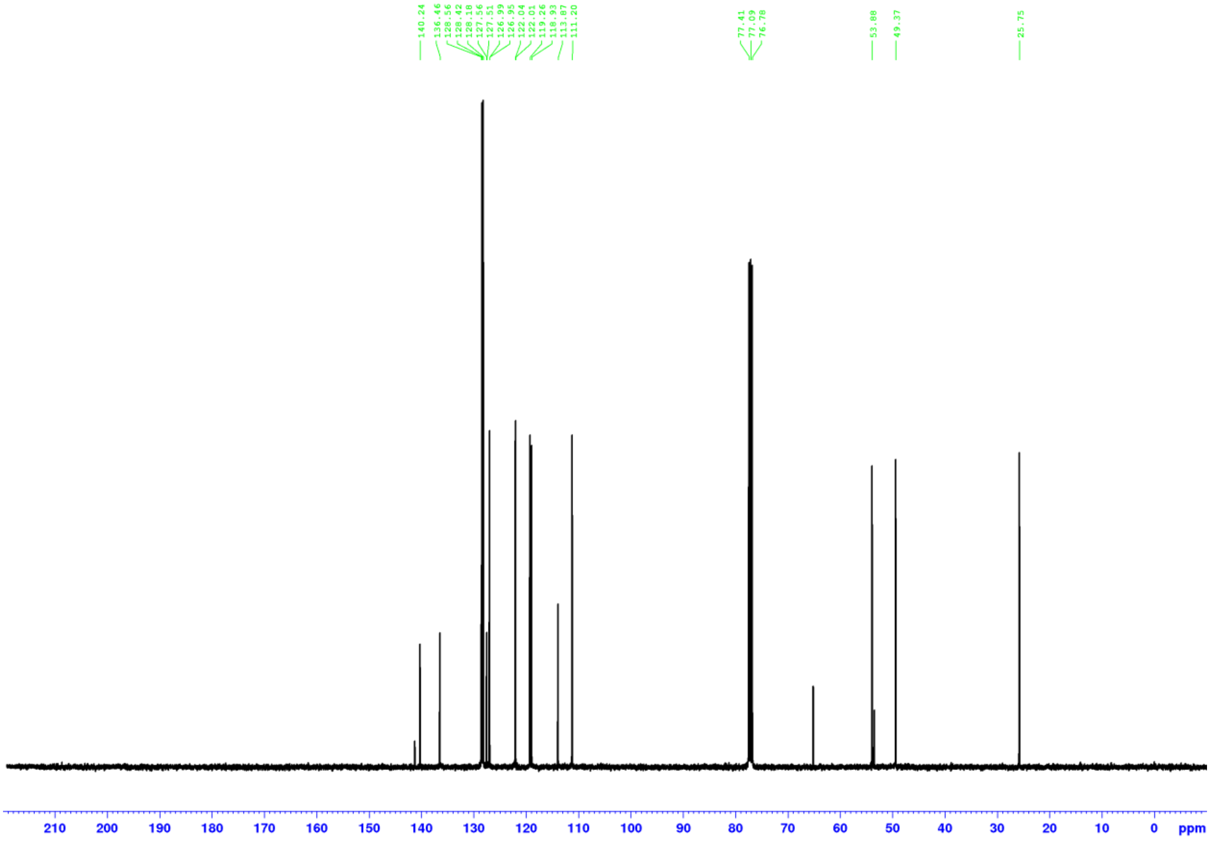

Figure S37. ^{13}C {1H} NMR ($CDCl_3$, 100 MHz) spectrum of diamine compound **11c**

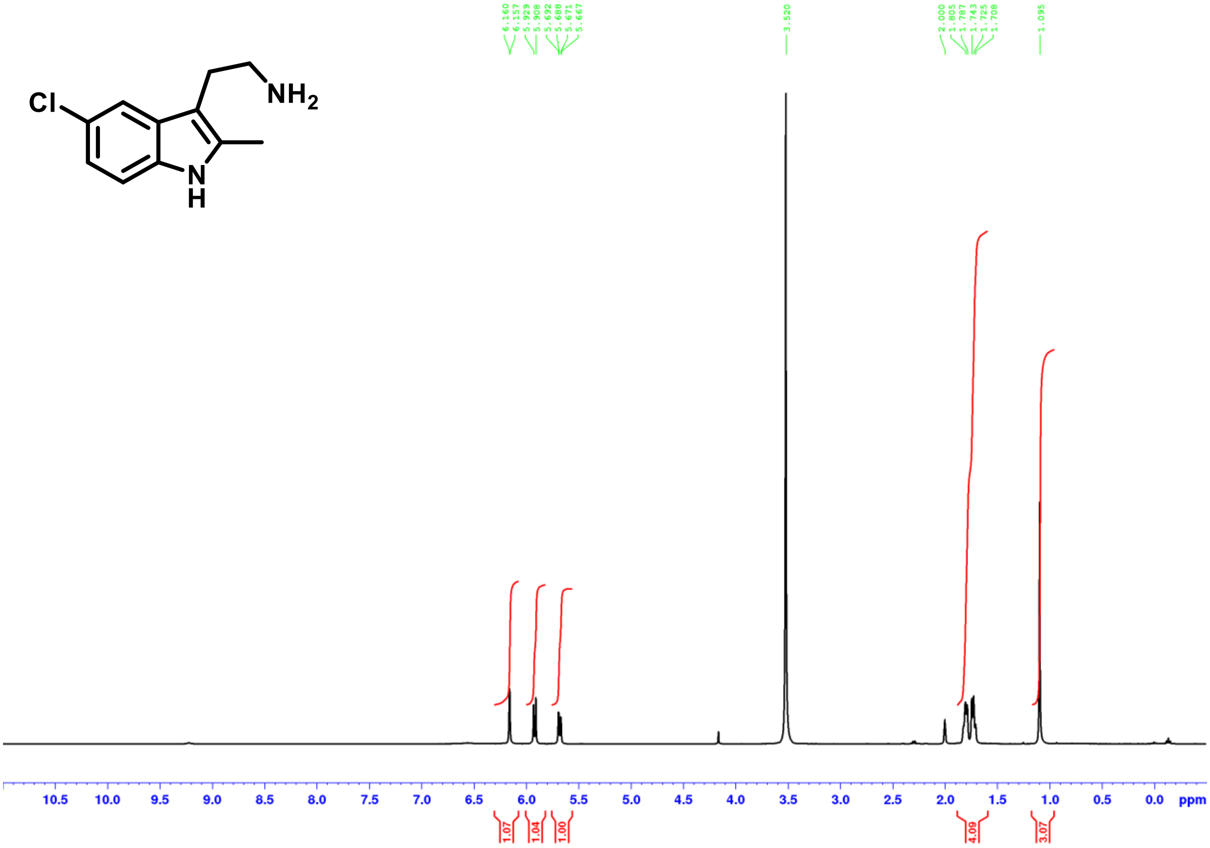

Figure S38. ^{1}H NMR (methanol-d_4, 400 MHz) spectrum of diamine compound **11d**

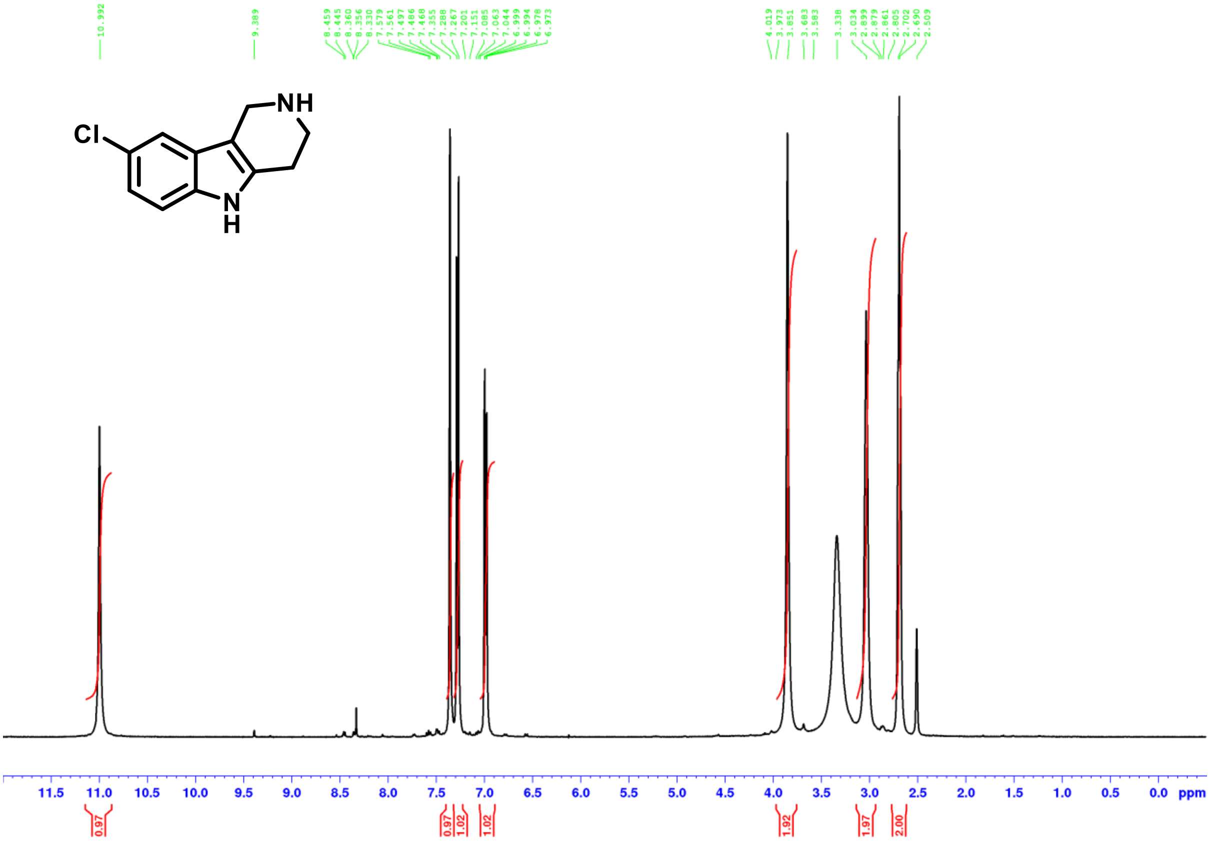

Figure S39. ^{1}H NMR (DMSO-d_6 400 MHz) spectrum of diamine compound **11e**

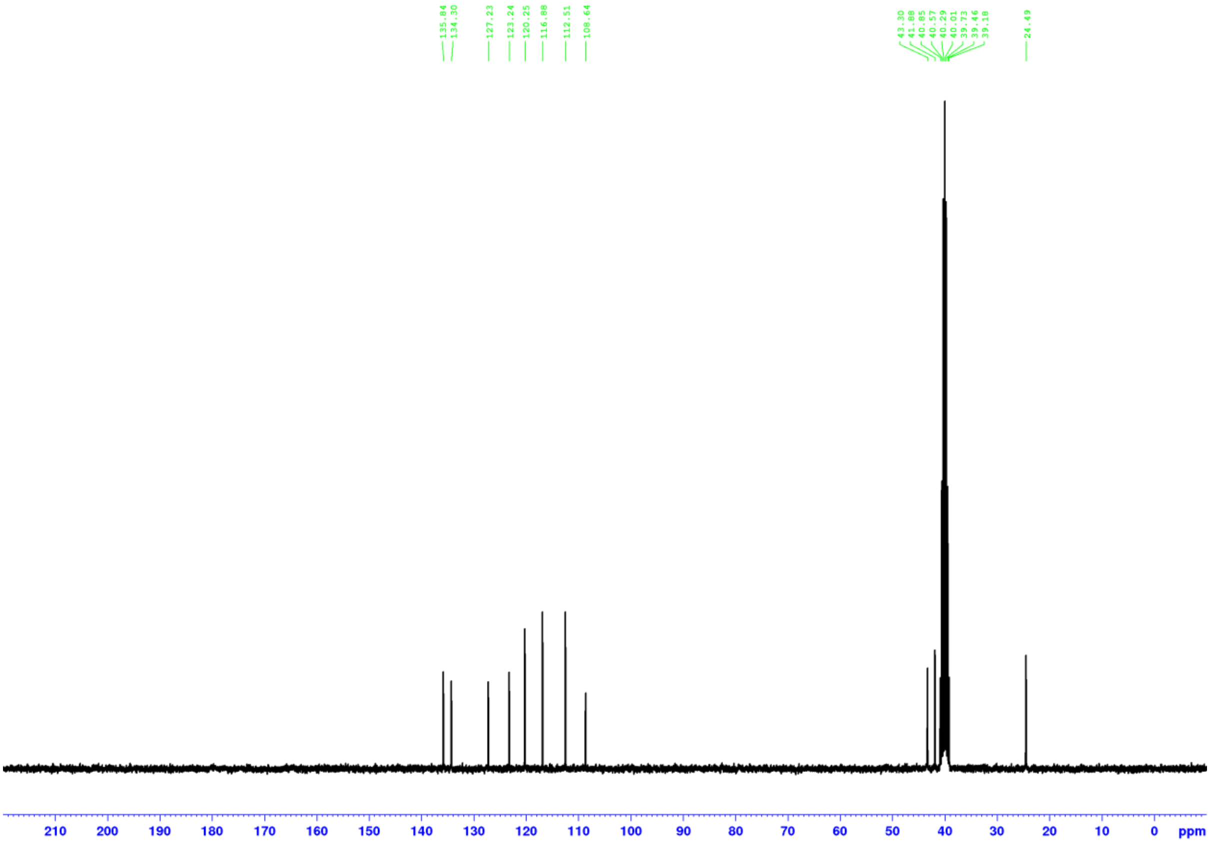

Figure S40. ^{13}C {1H} NMR (DMSO-d_6, 75 MHz) spectrum of diamine compound **11e**

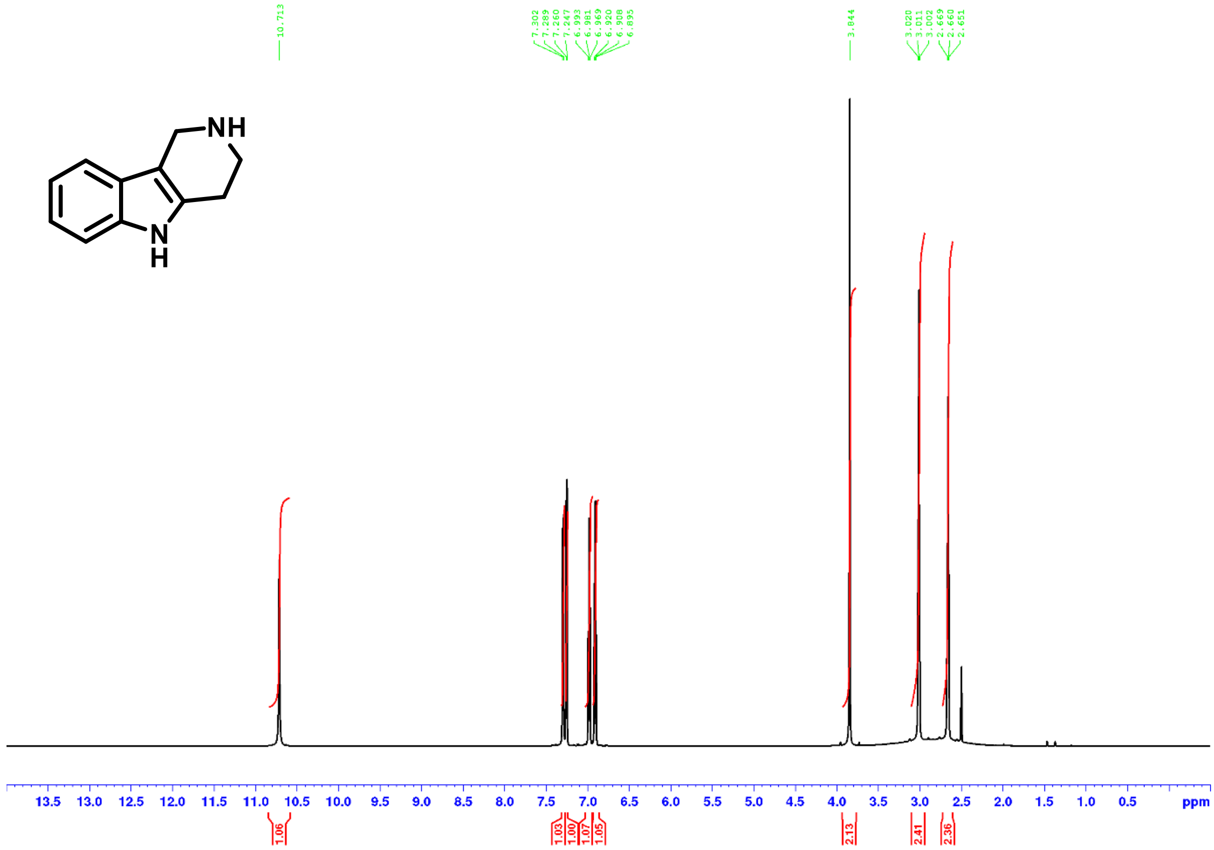

Figure S41. ^{1}H NMR (DMSO-d_6 600 MHz) spectrum of diamine compound **11f**

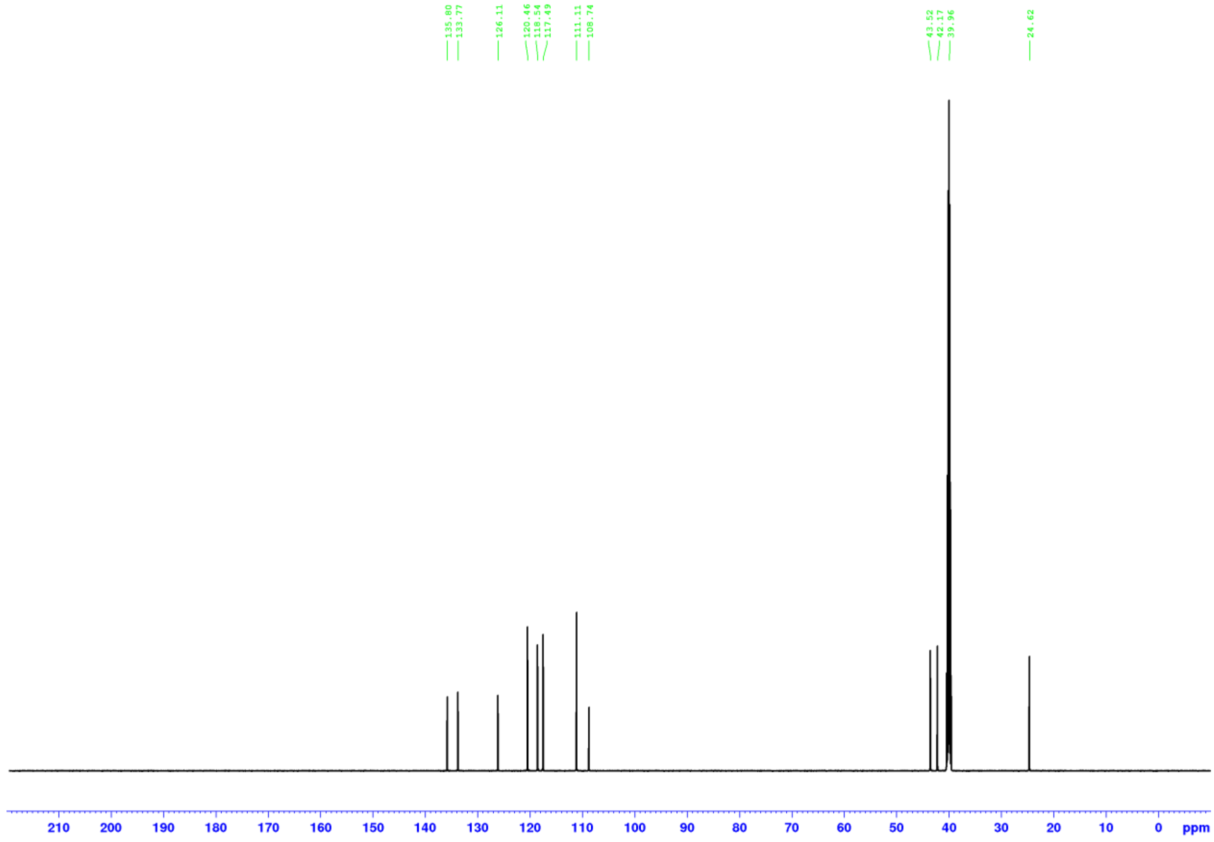

Figure S42. ^{13}C {1H} NMR (DMSO-d_6, 150 MHz) spectrum of diamine compound **11f**

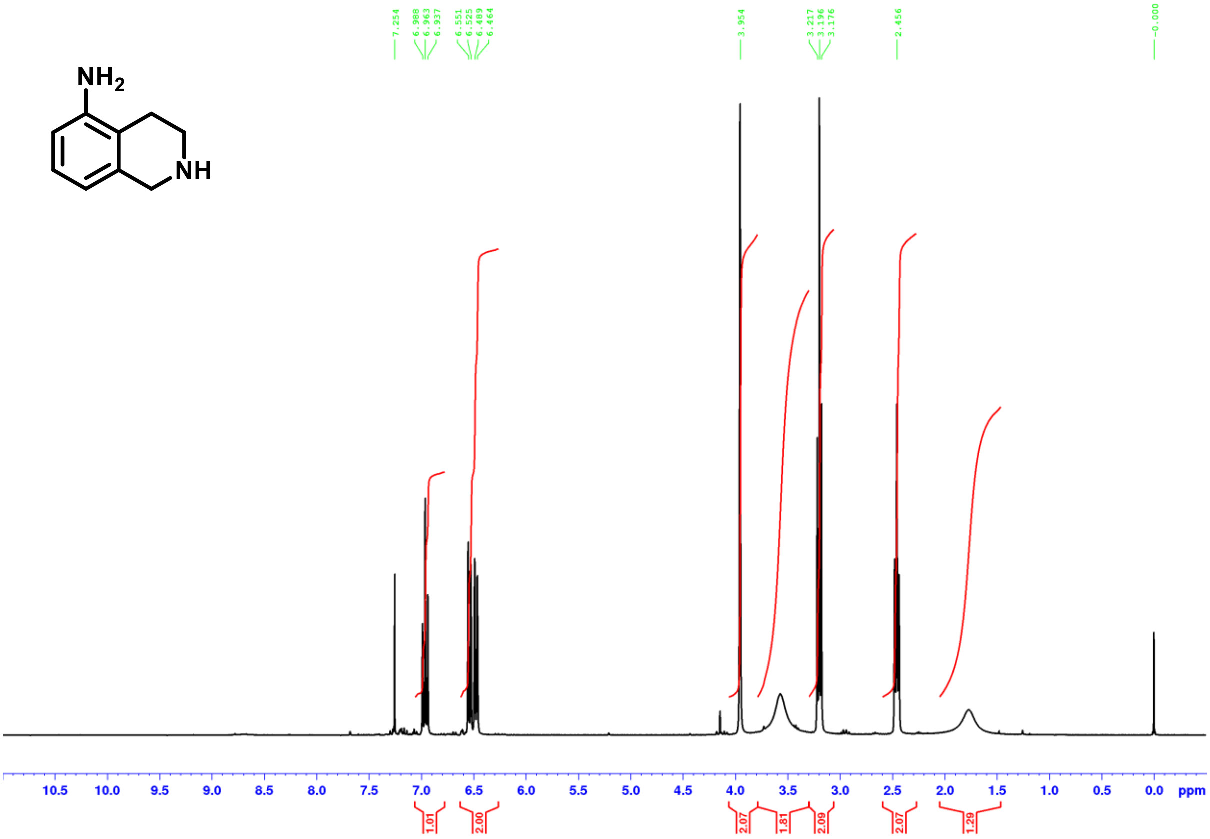

Figure S43. ^{1}H NMR ($CDCl_3$, 400 MHz) spectrum of diamine compound **11g**

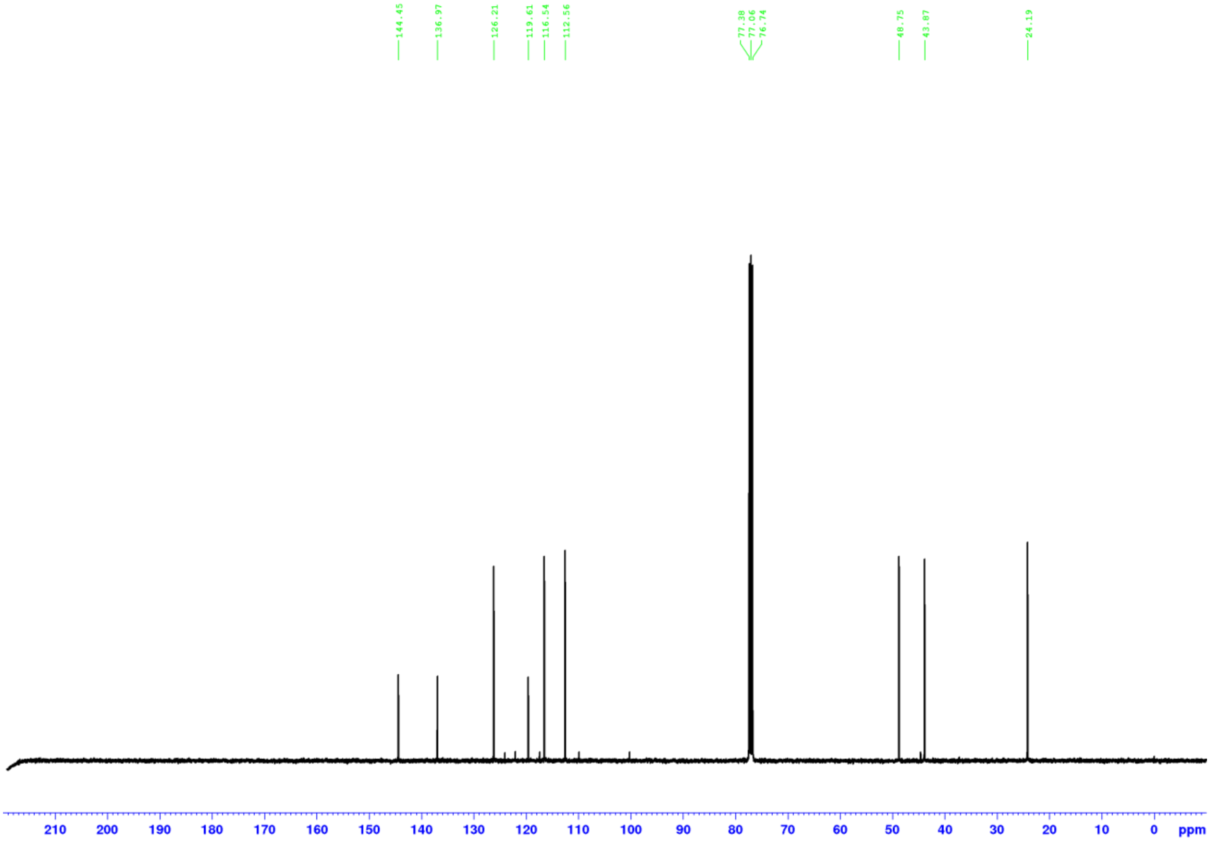

Figure S44. ^{13}C {1H} NMR ($CDCl_3$, 100 MHz) spectrum of diamine compound **11g**

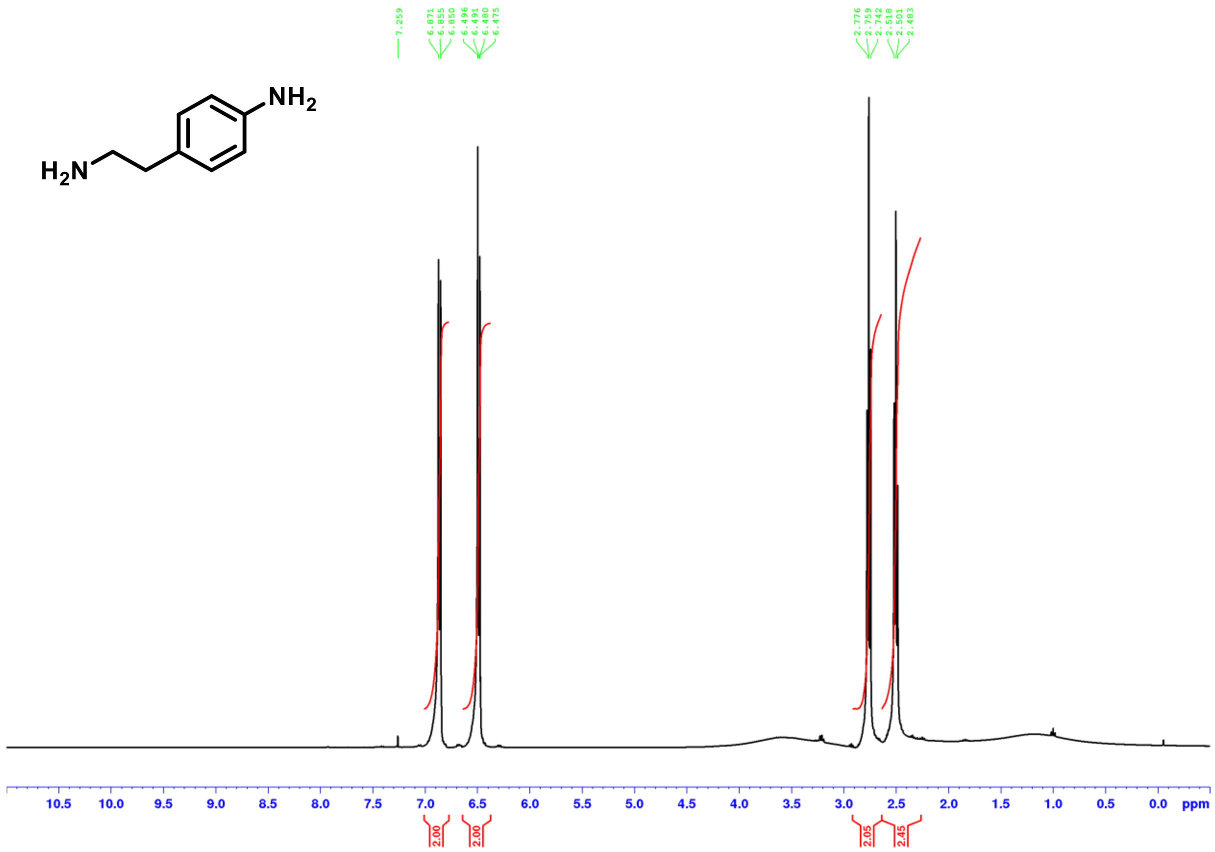

Figure S45. ^{1}H NMR ($CDCl_3$, 400 MHz) spectrum of diamine compound **11h**

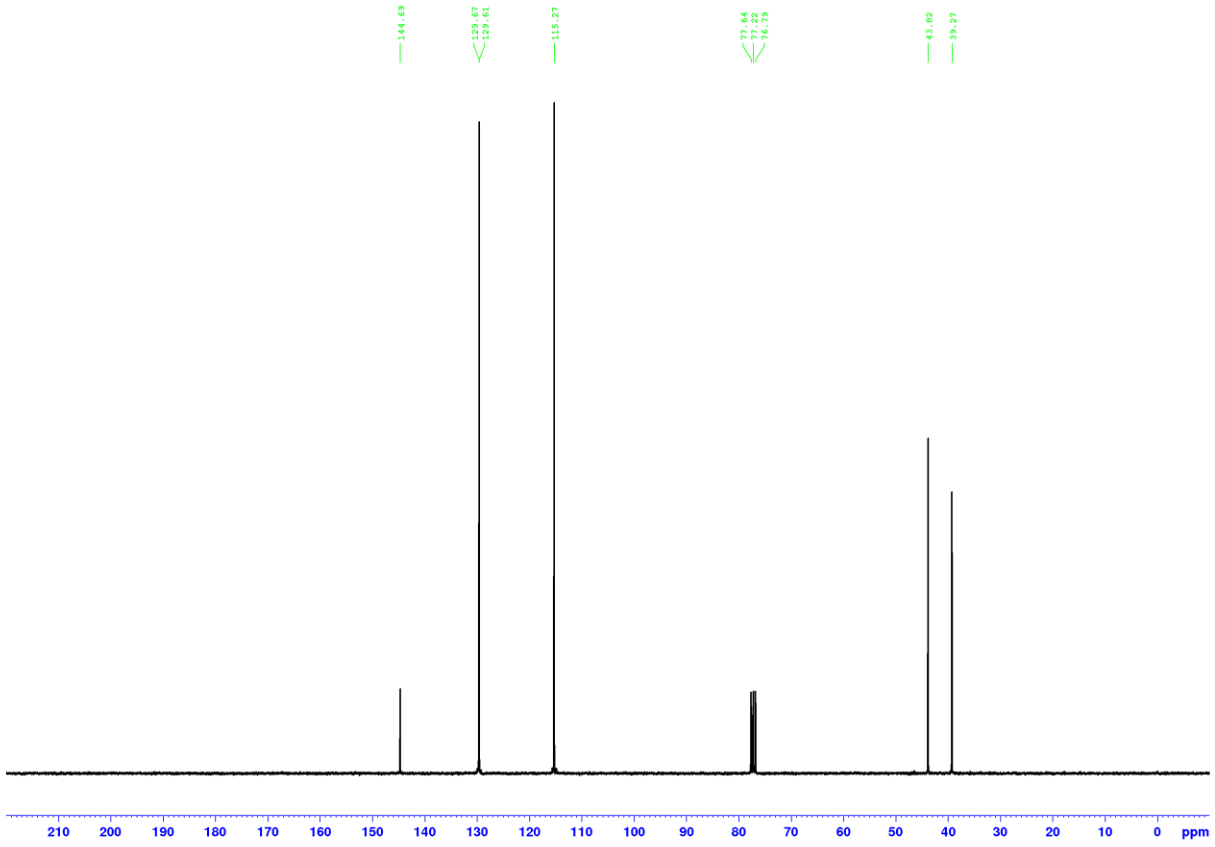

Figure S46. ^{13}C {1H} NMR ($CDCl_3$, 100 MHz) spectrum of diamine compound **11h**

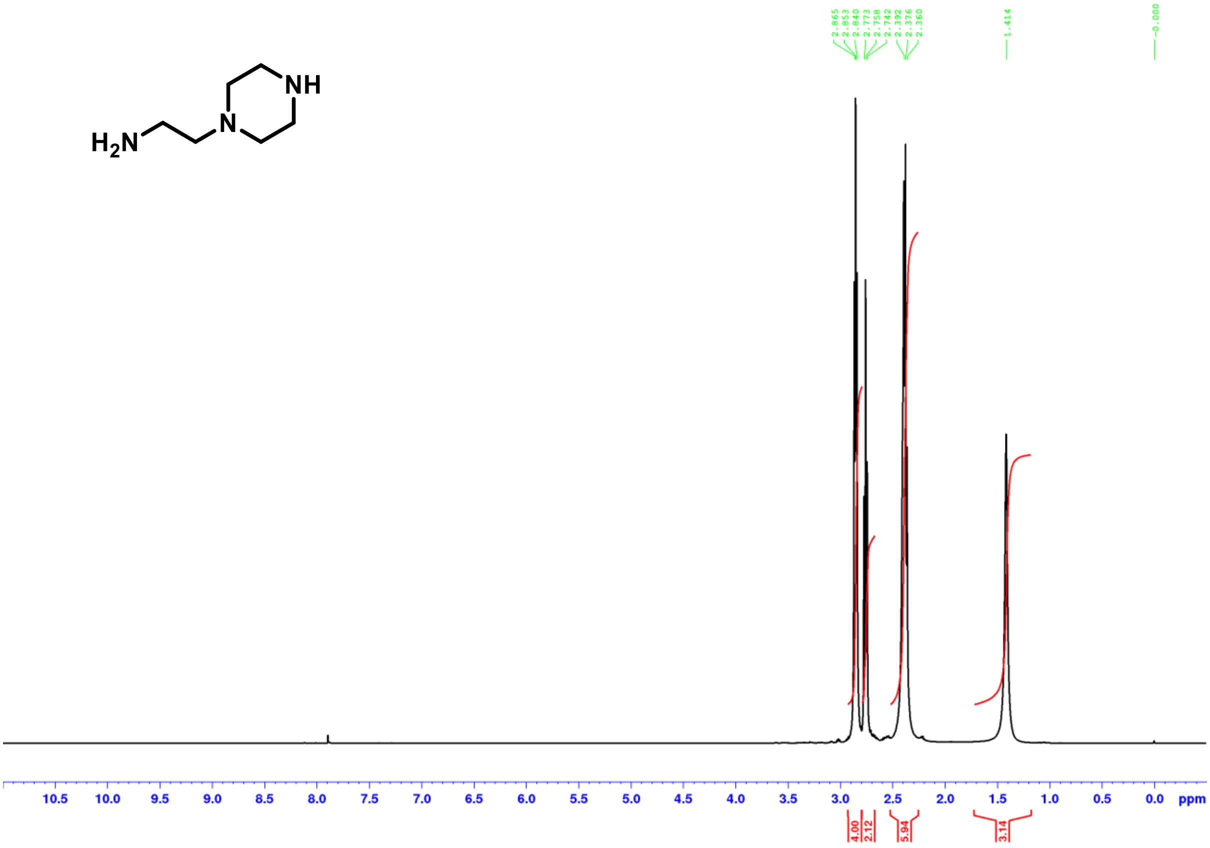

Figure S47. ^{1}H NMR ($CDCl_3$, 400 MHz) spectrum of diamine compound **11i**

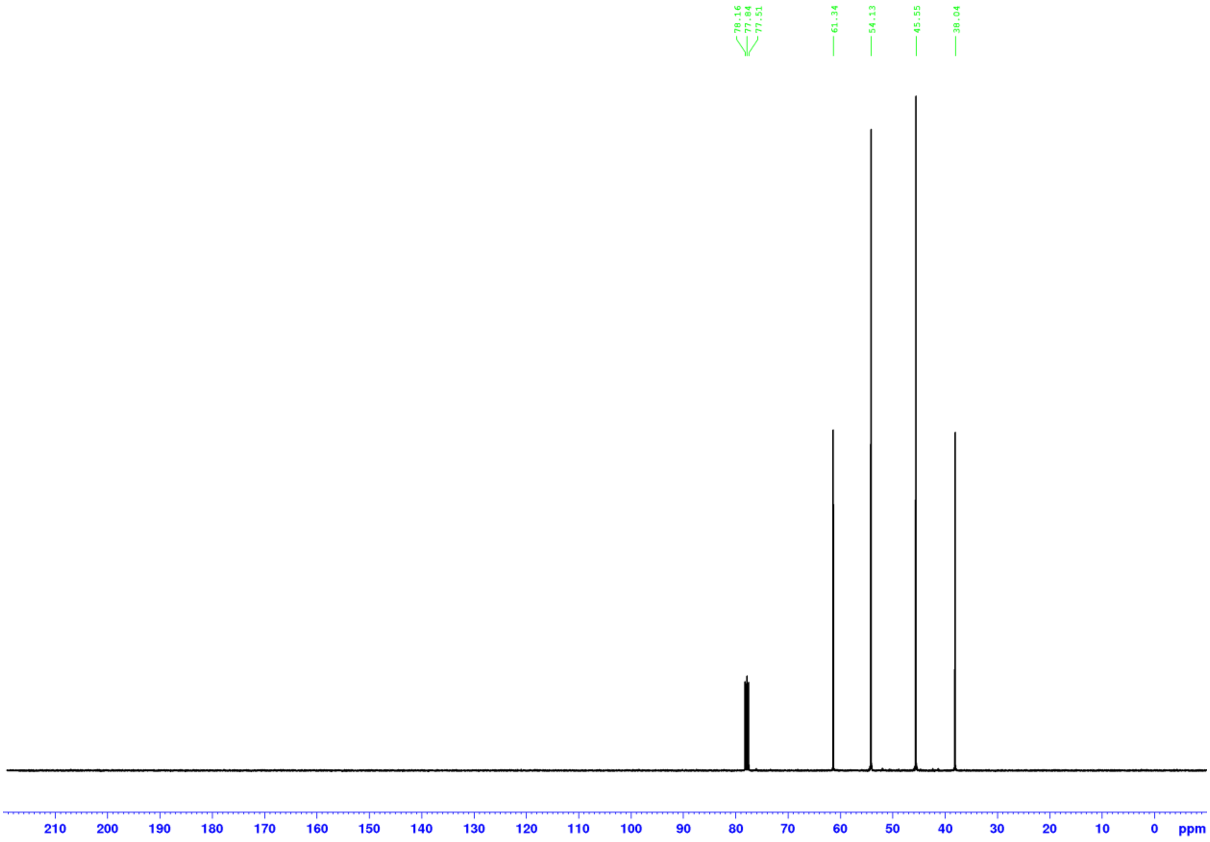

Figure S48. ^{13}C {1H} NMR ($CDCl_3$, 100 MHz) spectrum of diamine compound **11i**

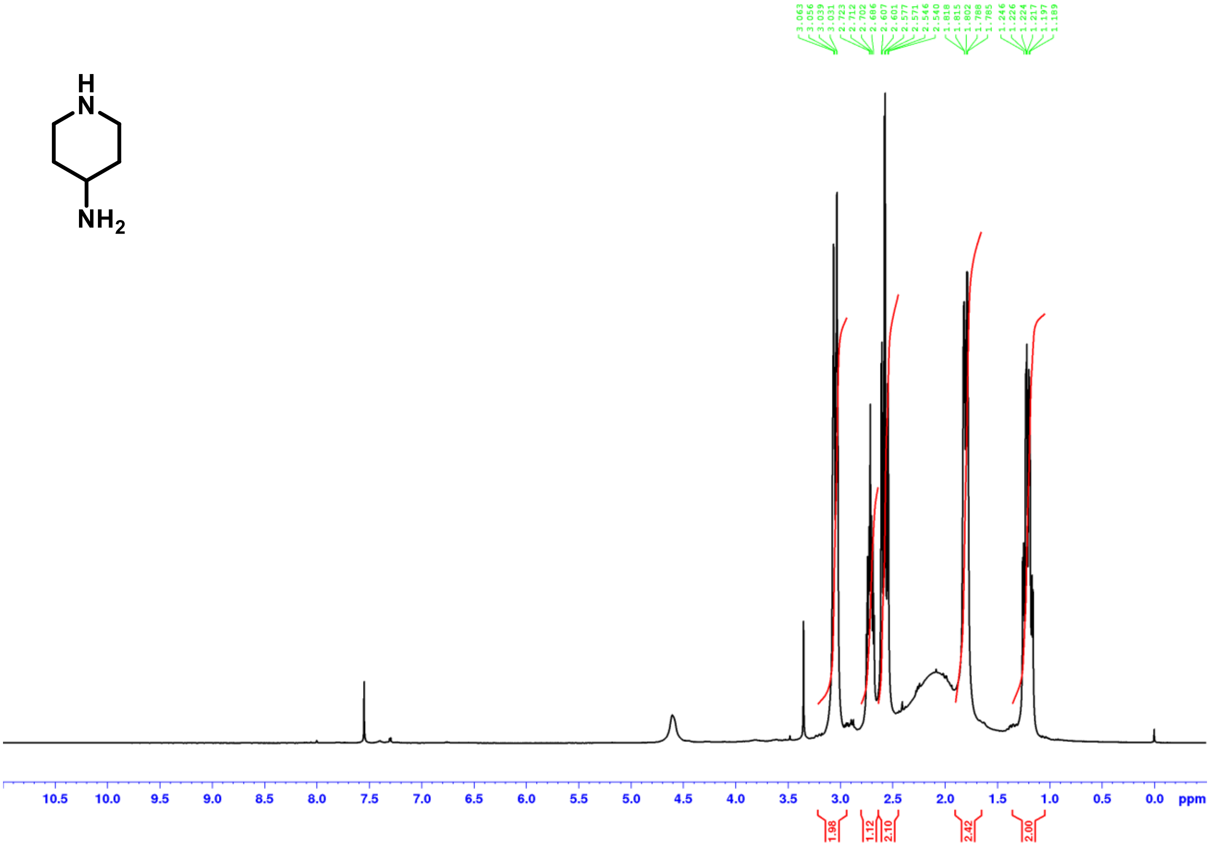

Figure S49. ^{1}H NMR ($CDCl_3$, 400 MHz) spectrum of diamine compound **11j**

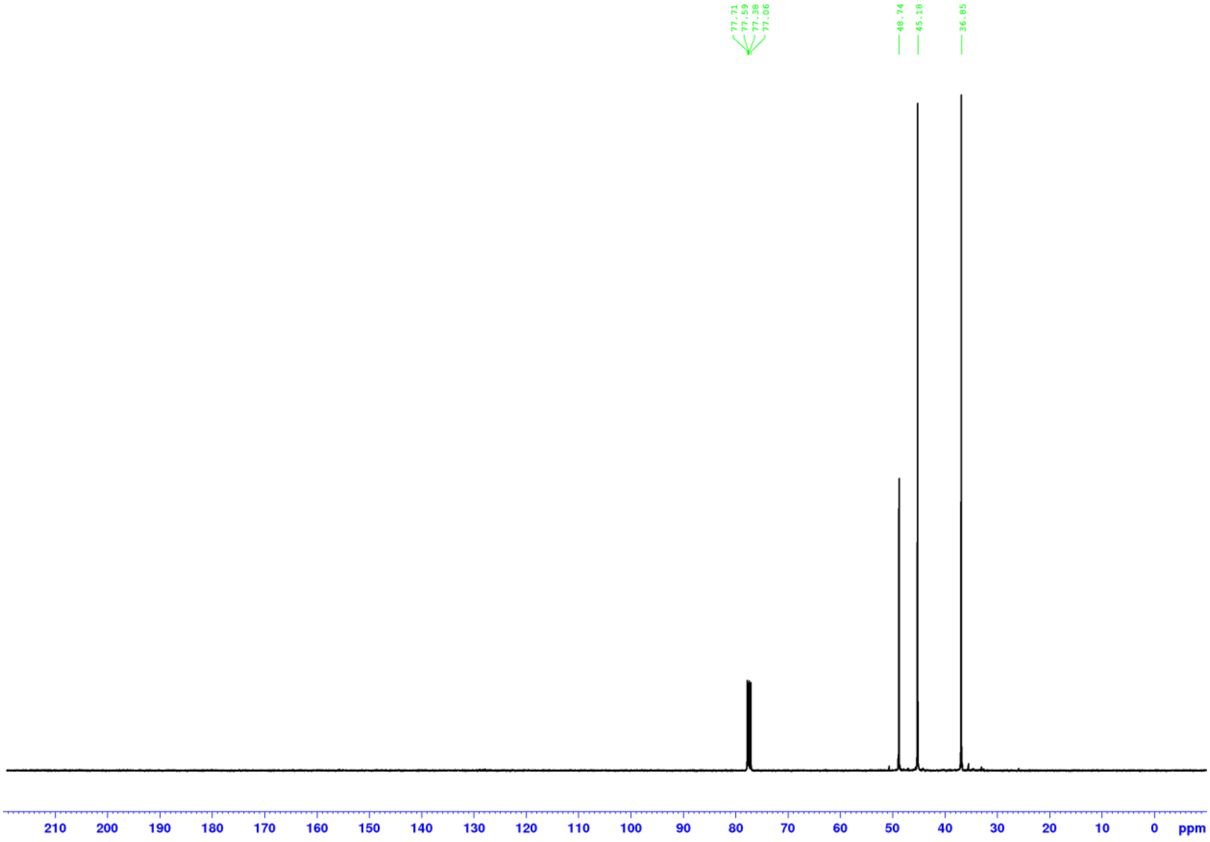

Figure S50. ^{13}C {1H} NMR ($CDCl_3$, 100 MHz) spectrum of diamine compound **11j**

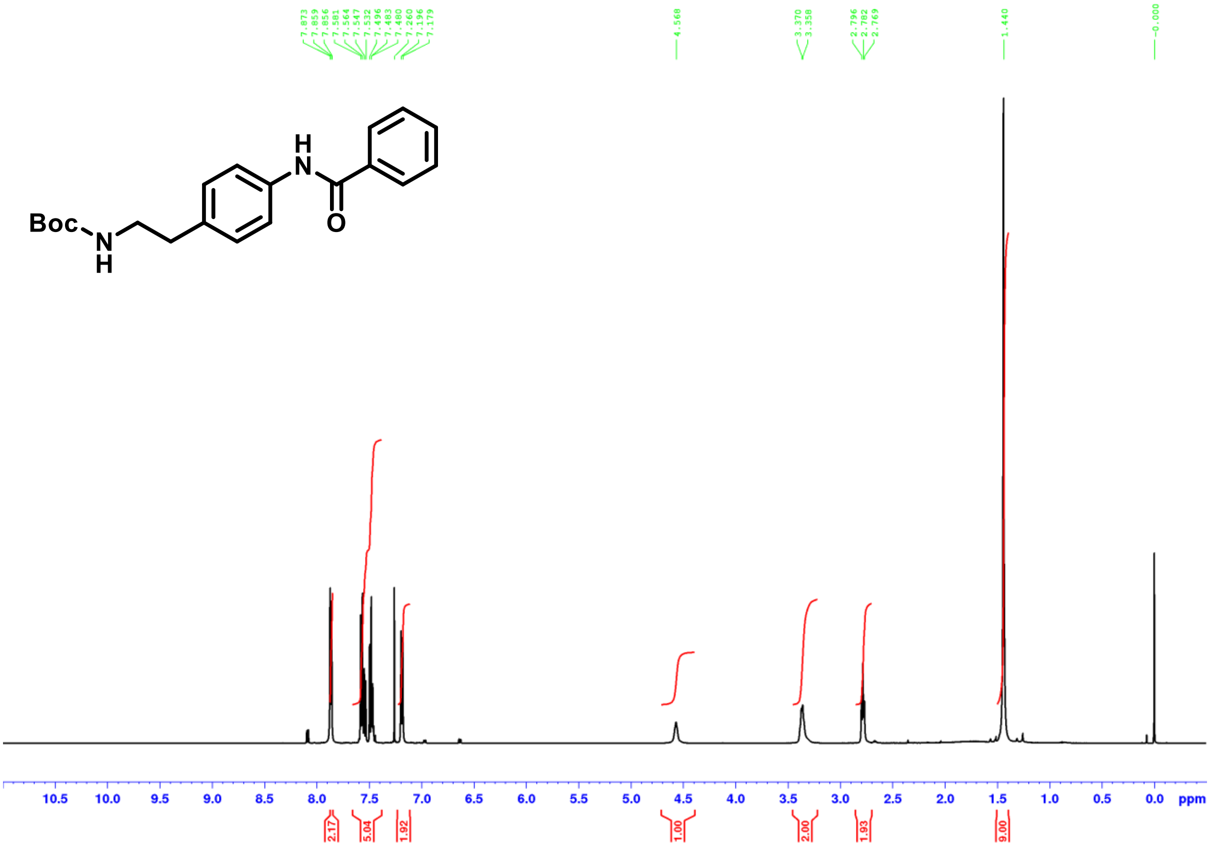

Figure S51. ^{1}H NMR ($CDCl_3$, 400 MHz) spectrum of diamine compound **12**

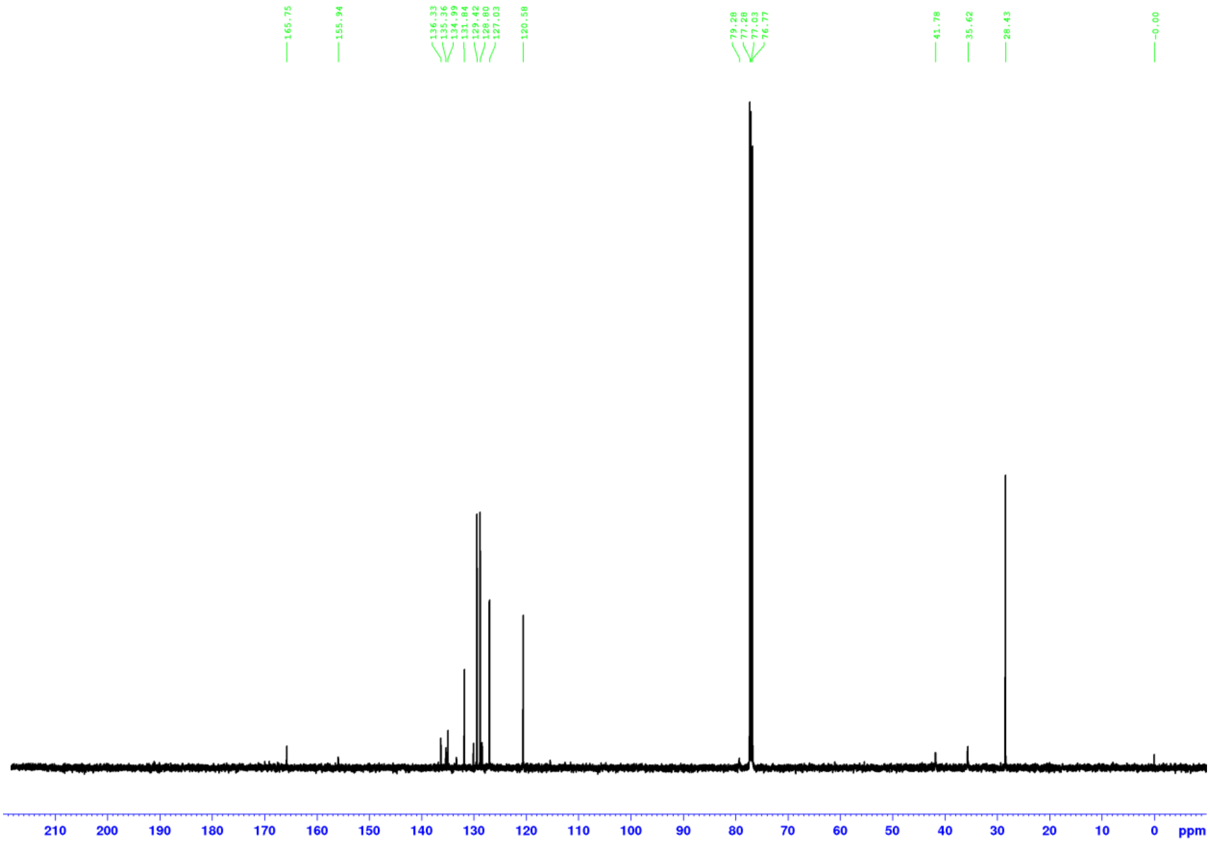

Figure S52. ^{13}C {1H} NMR ($CDCl_3$, 100 MHz) spectrum of diamine compound **12**

References:

(1) Vilaivan, T. A rate enhancement of *tert*-butoxycarbonylation of aromatic amines with Boc_2O in alcoholic solvents. ***Tetrahedron Lett.*** **2006**, *47* (38), 6739–6742.

(2) Basel, Y.; Hassner, A. Di-*tert*-butyl Dicarbonate and 4-(Dimethylamino)pyridine Revisited. Their Reactions with Amines and Alcohols[1]. ***J. Org. Chem.*** **2000**, *65* (20), 6368–6380.

(3) He, X.; Li, M.; Ye, W.; Zhou, W. Discovery of degradable niclosamide derivatives able to specially inhibit small cell lung cancer (SCLC). ***Bioorg. Chem.*** **2021**, *107*, 104574.

(4) Ingale, A. P.; Shinde, S. V.; Thorat, N. M. Sulfated tungstate: A highly efficient, recyclable and ecofriendly catalyst for chemoselective N-*tert* butyloxycarbonylation of amines under the solvent-free conditions. ***Synth. Commun.*** **2021**, *51* (16), 2528–2543.

(5) Choi, G.; Hong, S. H. Selective Monomethylation of Amines with Methanol as the C1 Source. ***Angew. Chem. Int. Ed.*** **2018**, *57* (21), 6166–6170.

(6) Deb, B.; Debnath, S.; Deb, A.; Maiti, D. K.; Majumdar, S. Copper nanoparticles catalyzed N–H functionalization: An efficient solvent-free N-*tert*-butyloxycarbonylation strategy. ***Tetrahedron Lett.*** **2017**, *58* (7), 629–633.

(7) Buchman, M.; Farney, E. P.; Greszler, S. N.; Altenbach, R. J.; Gfesser, G. A.; Voight, E. A. Lithioarene Cycliacylation and Pd-Catalyzed Aminoethylation/Cyclization to Access Electronically Diverse Saturated Isoquinoline Derivatives. ***J. Org. Chem.*** **2022**, *87* (1), 776–789.

(8) Li, Y.; Wong, L. L. Multi-Functional Oxidase Activity of CYP102A1 (P450BM3) in the Oxidation of Quinolines and Tetrahydroquinolines. ***Angew. Chem. Int. Ed.*** **2019**, *58* (28), 9551–9555.

(9) Li, J.; Wang, Z.-X. Nickel-catalyzed C–O bond reduction of aryl and benzyl 2-pyridyl ethers. ***ChemComm.*** **2018**, *54* (17), 2138–2141.

(10) Pandit, S.; Pandey, V. K.; Adhikari, A. S.; Kumar, S.; Maurya, A. K.; Kant, R.; Majumdar, N. Palladium-Catalyzed Dearomative [4 + 2]-Cycloaddition toward Hydrocarbazoles. ***J. Org. Chem.*** **2023,** *88* (1), 97–105.

(11) Zhang, T.; Chen, Z.; Tian, Y.; Han, B.; Zhang, N.; Song, W.; Liu, Z.; Zhao, J.; Liu, J. Kilogram-Scale Synthesis of Osteogenic Growth Peptide (10–14) Using a Fragment Coupling Approach. ***Org. Process Res. Dev.*** **2015**, *19* (9), 1257–1262.

(12) Maheswara Rao, B. L.; Nowshuddin, S.; Jha, A.; Divi, M. K.; Rao, M. N. A. New reagent for the introduction of Boc protecting group to amines: Boc-OASUD. ***Synth. Commun.*** **2017**, *47* (22), 2127–2132.

(13) Dossetter, A. G.; Beeley, H.; Bowyer, J.; Cook, C. R.; Crawford, J. J.; Finlayson, J. E.; Heron, N. M.; Heyes, C.; Highton, A. J.; Hudson, J. A.; et al. (1*R*,2*R*)-*N*-(1-Cyanocyclopropyl)-2-(6-methoxy-1,3,4,5-tetrahydropyrido[4,3-*b*]indole-2-

carbonyl)cyclohexanecarboxamide (AZD4996): A Potent and Highly Selective Cathepsin K Inhibitor for the Treatment of Osteoarthritis. ***J. Med. Chem.*** **2012**, *55* (14), 6363–6374.
(14) Liang, X.-W.; Liu, C.; Zhang, W.; You, S.-L. Asymmetric fluorinative dearomatization of tryptamine derivatives. ***ChemComm.*** **2017**, *53* (40), 5531–5534.
(15) Abe, T.; Yamada, K. Dehydrative Mannich-Type Reaction for the Synthesis of Azepinobisindole Alkaloid Iheyamine A. ***Org. Lett.*** **2018**, *20* (5), 1469–1472.
(16) Hsu, H.-C.; Hou, D.-R. Reduction of 1-pyrrolyl and 1-indolyl carbamates to hemiaminals. ***Tetrahedron Lett.*** **2009**, *50* (51), 7169–7171.
(17) Xie, W.; Jiang, G.; Liu, H.; Hu, J.; Pan, X.; Zhang, H.; Wan, X.; Lai, Y.; Ma, D. Highly Enantioselective Bromocyclization of Tryptamines and Its Application in the Synthesis of (−)-Chimonanthine. ***Angew. Chem. Int. Ed.*** **2013**, *52* (49), 12924–12927.
(18) Peng, H.; Zhou, Y.; Liu, J.; Zhang, H.; Xia, C.; Zhou, X. Synthesis of novel amino-functionalized ionic liquids and their application in carbon dioxide capture. ***RSC Adv.*** **2013**, *3* (19), 6859–6864.
(19) Caiger, L.; Zhao, H.; Constantin, T.; Douglas, J. J.; Leonori, D. The Merger of Aryl Radical-Mediated Halogen-Atom Transfer (XAT) and Copper Catalysis for the Modular Cross-Coupling-Type Functionalization of Alkyl Iodides. ***ACS Catal.*** **2023**, *13* (7), 4985–4991.
